# METTL3 acetylation impedes cancer metastasis via fine-tuning its nuclear and cytosolic functions

**DOI:** 10.1038/s41467-022-34209-5

**Published:** 2022-10-26

**Authors:** Yuanpei Li, Xiaoniu He, Xiao Lu, Zhicheng Gong, Qing Li, Lei Zhang, Ronghui Yang, Chengyi Wu, Jialiang Huang, Jiancheng Ding, Yaohui He, Wen Liu, Ceshi Chen, Bin Cao, Dawang Zhou, Yufeng Shi, Juxiang Chen, Chuangui Wang, Shengping Zhang, Jian Zhang, Jing Ye, Han You

**Affiliations:** 1 State Key Laboratory of Cellular Stress Biology, Innovation Center for Cell Signaling Network, School of Life Sciences, Xiamen University, 361102 Xiamen, China; 2 Wuxi Cancer Institute, Affiliated Hospital of Jiangnan University, 214062 Wuxi, China; 3 Department of Biochemistry and Molecular Biology, Capital Medical University, 100069 Beijing, China; 4 State Key Laboratory of Cellular Stress Biology, School of Pharmaceutical Sciences, Xiamen University, 361102 Xiamen, China; 5 Key Laboratory of Animal Models and Human Disease Mechanisms of Chinese Academy of Sciences & Yunnan Province, Kunming Institute of Zoology, Chinese Academy of Sciences, 650223 Kunming, China; 6 Fujian Provincial Key Laboratory of Reproductive Health Research, School of Medicine, Xiamen University, 361102 Xiamen, China; 7 Tongji University Cancer Center, Shanghai Tenth People’s Hospital of Tongji University, School of Medicine, Tongji University, 200092 Shanghai, China; 8 Department of Neurosurgery, Shanghai Changhai Hospital, Naval Medical University, 200433 Shanghai, China; 9 The Biomedical Translational Research Institute, School of Life Sciences, Shandong University of Technology, 255049 Zibo, China; 10 Translational Medicine Center, Shanghai General Hospital, Shanghai Jiao Tong University School of Medicine, 201620 Shanghai, China; 11 The State Key Laboratory of Cancer Biology, Department of Biochemistry and Molecular Biology, Fourth Military Medical University, 710032 Xi’an, China; 12 Department of Pathology, Xijing Hospital and School of Basic Medicine, Fourth Military Medical University, 710032 Xi’an, China

**Keywords:** Cell signalling, Cancer

## Abstract

The methyltransferase like 3 (METTL3) has been generally recognized as a nuclear protein bearing oncogenic properties. We find predominantly cytoplasmic METTL3 expression inversely correlates with node metastasis in human cancers. It remains unclear if nuclear METTL3 is functionally distinct from cytosolic METTL3 in driving tumorigenesis and, if any, how tumor cells sense oncogenic insults to coordinate METTL3 functions within these intracellular compartments. Here, we report an acetylation-dependent regulation of METTL3 localization that impacts on metastatic dissemination. We identify an IL-6-dependent positive feedback axis to facilitate nuclear METTL3 functions, eliciting breast cancer metastasis. IL-6, whose mRNA transcript is subjected to METTL3-mediated m^6^A modification, promotes METTL3 deacetylation and nuclear translocation, thereby inducing global m^6^A abundance. This deacetylation-mediated nuclear shift of METTL3 can be counterbalanced by SIRT1 inhibition, a process that is further enforced by aspirin treatment, leading to ablated lung metastasis via impaired m^6^A methylation. Intriguingly, acetylation-mimetic METTL3 mutant reconstitution results in enhanced translation and compromised metastatic potential. Our study identifies an acetylation-dependent regulatory mechanism determining the subcellular localization of METTL3, which may provide mechanistic clues for developing therapeutic strategies to combat breast cancer metastasis.

## Abstract (web summary)

METTL3 catalyzes mRNA m^6^A deposition. The authors identify an acetylation-mediated regulation of METTL3 subcellular localization and compartment-specific functions, a process that is fine-tuned by anti-inflammatory and pro-inflammatory signals, which ultimately determine breast cancer metastasis.

## Introduction

A growing body of evidence has suggested the involvement of m^6^A modification in regulating a wide range of biological events, including mRNA turnover, RNA splicing, gene transcription and protein translation^1–13^. Dysregulated m^6^A homeostasis may therefore influence a variety of pathophysiological processes, leading to altered cell fate decisions and human diseases^14–21^. A multiprotein complex, including METTL3, METL14, WTAP, and several additional subunits, has been reported to catalyze m^6^A methylation on thousands of poly(A)^+^ transcripts^22–24^. Structure analysis has revealed that METTL3 functions as the catalytic core, while METTL14 serves as an RNA-binding platform^25^. WTAP targets METTL3 and METTL14 into nuclear speckles^23^.

In addition to catalyzing m^6^A modification, METTL3 has been reported to modulate gene transcription or protein translation via distinct mechanisms^7,18,26–29^. For instances, cytosolic localized METTL3 can facilitate protein translation in an m^6^A-independent manner but requires eIF3h through mRNA looping mechanism^27,28^, while promoter-bound METTL3 can enhance protein translation by relieving ribosome stalling in an m^6^A-dependent manner^26^. These studies implied that the subcellular distribution of METTL3 is a fundamental determinant of its physiological functions. METTL3 localization varies in a cell-type and stress-type-dependent manner^30,31^. However, molecular determinants governing the dynamic subcellular localization of METTL3 and its functional consequences are poorly understood.

The role of METTL3 in tumorigenicity has been extensively studied. A number of studies have shown that METTL3 depletion suppresses cell viability and represses tumorigenesis^22,26–28,32^. Here, we report a prominent role of acetylation in modulating METTL3 localization and its subsequent impact on tumorigenic progression. The identification of a feedback activation loop between IL-6 signaling and nuclear METTL3 function, which can be blunted by aspirin and nicotinamide co-treatment, unravels a critical interplay between inflammatory/oncogenic insults and RNA epigenetic state, which expedites breast cancer metastasis.

## Results

### METTL3 nuclear accumulation correlates with node metastasis and invasiveness of breast cancer cells

**Fig. 1:**
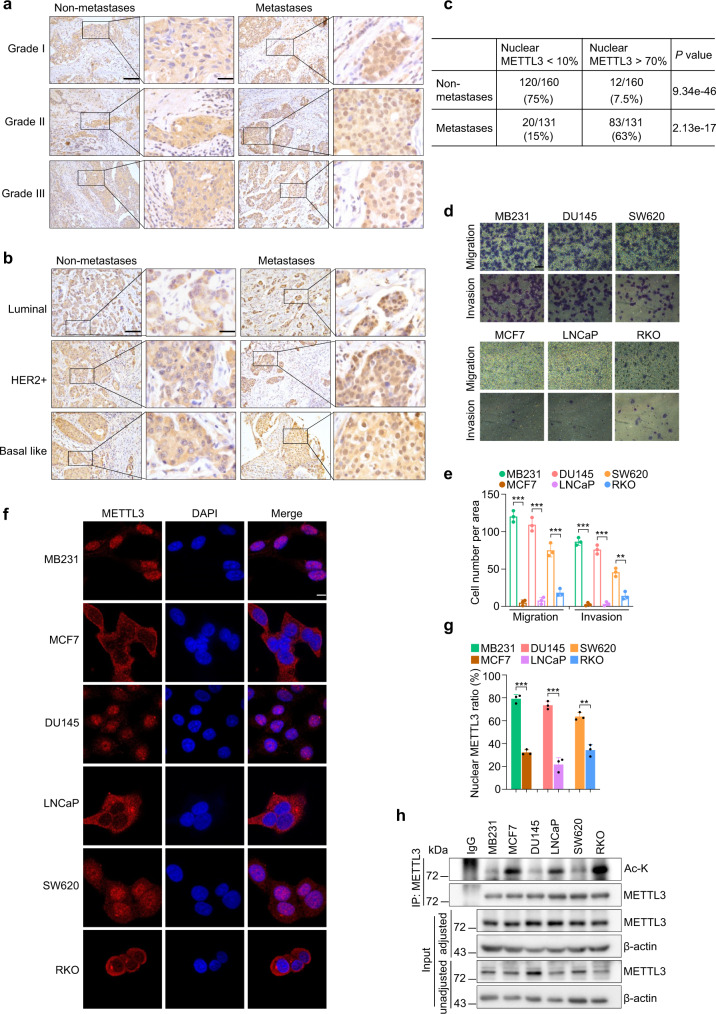
METTL3 nuclear accumulation correlates with node metastasis and invasiveness of breast cancer cells. **a**, **b** Representative images of METTL3 IHC staining in the indicated breast carcinomas. Grade I: non-metastases *n* = 30, metastases *n* = 63; Grade II: non-metastases *n* = 105, metastases *n* = 48; Grade III: non-metastases *n* = 25, metastases *n* = 20. Luminal: non-metastases *n* = 12, metastases *n* = 24; HER2+: non-metastases *n* = 9, metastases *n* = 11; Basal like: non-metastases *n* = 7, metastases *n* = 7. Scale bars, 100 µm for low magnification (10×, left panel), and 25 µm for high magnification (40×, right panel). **c** Table demonstrating the correlation between positive nuclear METTL3 staining and node metastasis. *P* values by two-sided *t* test. **d**, **e** Migration assay (for MDA-MB-231, MCF-7, DU145, and LNCaP cell lines, 4 h; for SW-620 and RKO cell lines, 12 h) and invasion assay (for MDA-MB-231, MCF-7, DU145, and LNCaP cell lines, 8 h; for SW-620 and RKO cell lines, 24 h) were carried out as indicated (*n* = 3 biologically independent experiments). Scale bars, 50 μm. Data are represented as mean ± SD. From left to right **e**: ^***^P = 1.79e-05, ^***^P = 6.43e-05, ^***^P = 6.44e-04, ^***^P = 1.17e-05, ^***^P = 4.21e-05, ^**^P = 0.00199, respectively, by two-sided *t* test. **f**, **g** Representative immunofluorescence for METTL3 (red) and DAPI (blue, cell nuclei) in the indicated cell lines (*n* = 3 biologically independent experiments). Scale bars, 10 μm. Quantification of nuclear METTL3 percentage is presented as mean ± SD. From left to right **g**: ^***^P = 4.83e-05, ^***^P = 2.05e-04, ^**^P = 0.006, respectively, by two-sided *t* test. **h** Lysates of the indicated tumor cell lines were subjected to IP and IB analysis as indicated. Source data are provided as a Source Data file.

Dysregulated METTL3 signaling has been shown to potentiate cancer cell invasion and tumor metastasis^27^. To explore the clinical implications of METTL3 expression in breast cancer, particularly if its expression levels can distinguish breast cancer subtypes and histological grades, we assessed METTL3 expression in a cohort of 291 breast cancer patients. The overall METTL3 staining intensity showed no obvious correlation with the histological grades or subtypes by immunohistochemistry (IHC) staining (Fig. 1a, b and Supplementary Fig. 1a, b). However, when we grouped these clinical samples into non-metastases (*n* = 160) and metastases (*n* = 131) based on lymph node status, approximately 75% of non-metastases samples (120/160) had strong cytoplasmic staining of METTL3 but lacked METTL3 expression in the nucleus (<10% of counted cells showing positive nuclear staining of METTL3) (Fig. 1c). By contrast, in metastases group, around 63% samples (83/131) displayed a strong staining of METTL3 in the nucleus (>70% of counted cells exhibiting positive nuclear staining). Interestingly, in normal breast tissues, only weak staining of METTL3 was detected in luminal epithelial cells, in contrast to its moderate staining intensities in basal myoepithelial cells (Supplementary Fig. 1c). Of note, as shown in Supplementary Fig. 1d, strong nuclear METTL3 expression was detected in both tumor areas and ducts in adjacent regions in TNBC samples with LN metastasis. By contrast, in tumor samples without LN metastasis, ducts in adjacent areas displayed heterogeneous staining patterns, both nuclear-positive and -negative cells were detected. Other subunits of METTL3 complex, namely WTAP, RBM15, KIAA1429 (also known as Virilizer homolog or VIRMA), and METTL14, displayed moderate-to-strong staining intensities in both normal and breast cancer tissues (Supplementary Fig. 1e). Meanwhile, we examined METTL3 localization in paired primary breast cancers and its metastatic lesions. Around 84% (27/32) of these paired samples contained tumor cells with significantly stronger nuclear METTL3 expression, indicative a sustained nuclear localization of METTL3 post metastatic dissemination (Supplementary Fig. 1f). These data demonstrate a potential correlation between nuclear METTL3 levels and the occurrence of node metastasis in breast cancer patients. Similar results were obtained in a cohort of the prostate (*n* = 102) and colon (*n* = 151) cancer patients (Supplementary Fig. 1g–j).

To further test the hypothesis that METTL3 nuclear localization correlates with metastatic tumors and explore the underlying mechanisms, we characterized three pairs of tumor cell lines with distinct migration/invasion potential. MCF-7, LNCaP, and RKO cells displayed very weak migration/invasion capacity, in sharp contrast to MDA-MB-231, DU145, and SW-620 cells which were highly metastatic (Fig. 1d, e). Immunofluorescent staining (IF) and subcellular fractionation assay revealed predominantly cytosolic METTL3 in the non-invasive group, in contrast to its strong nuclear presence in invasive tumor cells (Fig. 1f, g and Supplementary Fig. 1k). Notably, the distribution pattern of METTL14 was similar to that of METTL3 (Supplementary Fig. 1k, l).

Extensive studies have revealed the critical roles of post-translational modifications in determining the subcellular localization of signaling molecules. METTL3 has been shown to undergo SUMOylation, phosphorylation, ubiquitination and methylation^33–35^. However, none of these modifications have been associated with the regulation of METTL3 distribution. We therefore assessed the overall methylation, acetylation or phosphorylation levels of METTL3 between invasive and non-invasive cells. Immunoblotting with the pan-acetylation antibody (Ac-K) following immunoprecipitating METTL3 revealed strong acetylation signals of endogenous METTL3 in non-invasive cells, in contrast to very faint acetylated band in invasive ones (Fig. 1h). By contrast, neither western blotting (WB) with the pan-methylation antibody following immunoprecipitation of METTL3 nor phos-tag gel followed by METTL3 immunoblotting detected any significant differences between the invasive and non-invasive groups (Supplementary Fig. 1m, n). Collectively, these data indicate a potential correlation among the invasiveness of cancer cells, the nuclear accumulation of METTL3, and METTL3 acetylation status.

### Acetylation of METTL3 at K177 disrupts migration and invasion potential of breast cancer cells

**Fig. 2:**
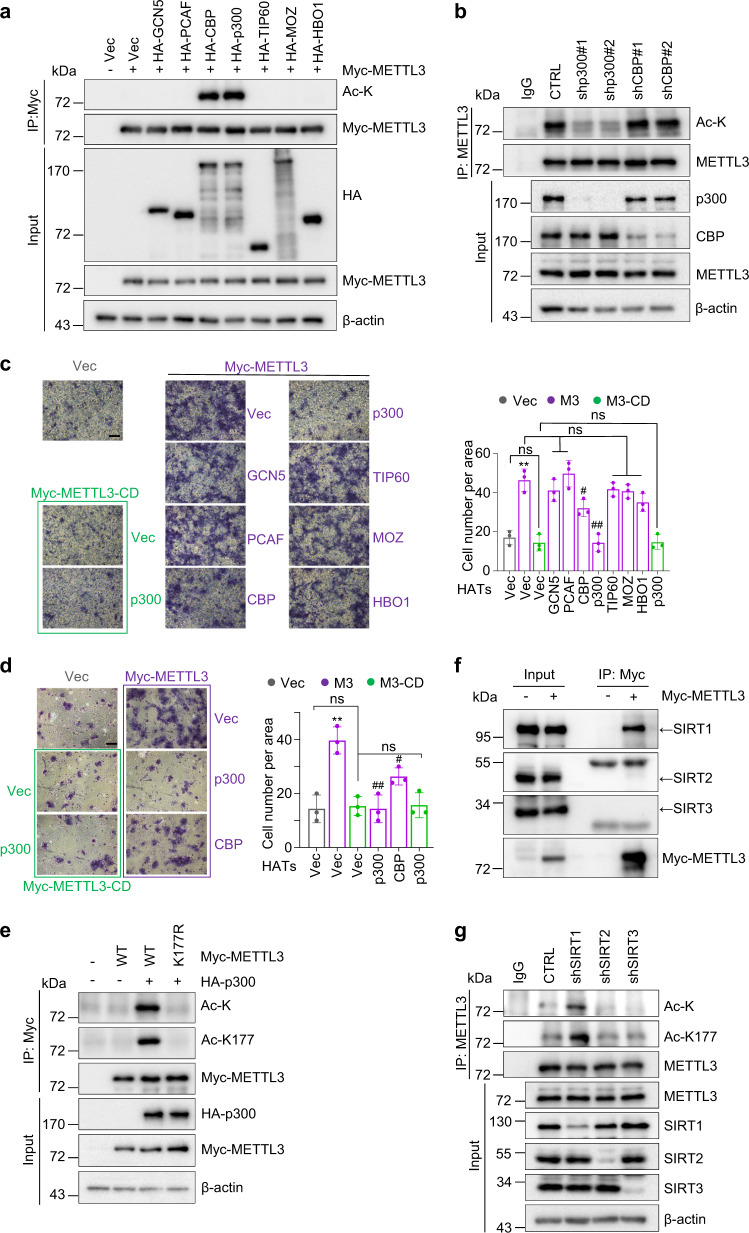
Acetylation of METTL3 at K177 disrupts migration and invasion potential of breast cancer cells. **a** Lysates of HEK293T cells transfected with the indicated constructs were subjected to IP and IB analysis. **b** MCF-7 cells were infected with the indicated lentiviruses. Lysates were subjected to IP and IB analysis. **c**, **d** MCF-7 cells stably expressing Myc-METTL3 or Myc-METTL3-CD were transfected with the indicated HATs expressing constructs, followed by migration and invasion assays (left panel) (*n* = 3 biologically independent experiments). Quantification is presented as mean ± SD (right panel). Scale bars, 50 μm. ^*^Compared to Vec. ^#^Compared to Myc-METTL3. ^**^P = 0.0017, ^#^*P* = 0.0257, ^##^*P* = 0.0016. From left to right **d**: ns *P* = 0.45, ns *P* = 0.31, ns *P* = 0.55, ns *P* = 0.29, ns *P* = 0.22, ns *P* = 0.055, ns *P* = 0.92, respectively, by two-sided *t* test. **e**, **f** Lysates of HEK293T cells transfected with the indicated constructs were subjected to IP and IB analysis. **g** MDA-MB-231 cells were infected with the indicated lentiviruses. Lysates were subjected to IP and IB analysis. Source data are provided as a Source Data file.

To screen for potential acetyltransferases for METTL3, Myc-METTL3 was co-transfected together with a panel of individual histone acetyltransferases (HATs) into HEK293T cells (Fig. 2a). Ectopic expression of p300 or CBP promoted the acetylation of METTL3, as detected by Ac-K antibody. However, the acetylation level of endogenous METTL3 was reduced only upon knockdown of *EP300* (which encodes p300), but not *CREBBP* (which encodes CBP) (Fig. 2b). Furthermore, only WT p300, but not its catalytically inactive forms (S1396R/Y1397R or D1399Y), catalyzed METTL3 acetylation in vitro and in vivo (Supplementary Fig. 2a). These results demonstrate that p300 serves as a physiological acetyltransferase for METTL3.

We next validated the biological consequences of METTL3 acetylation by p300. MCF-7 cells stably expressing wild-type (WT) METTL3 displayed much stronger migration/invasion potency, compared to control cells or cells expressing METTL3-CD (a catalytically inactive form of METTL3) (Fig. 2c, d), suggesting the methyltransferase activity is indispensable for METTL3-induced migration/invasion. Among all HATs tested (Supplementary Fig. 2b), only ectopic p300 completely blunted migration/invasion of METTL3-expressing cells (Fig. 2c, d), demonstrating that p300 counteracts with METTL3 in promoting tumor cell invasiveness via catalyzing its acetylation.

To identify potential acetylation sites on METTL3, we performed stable isotope labeling of amino acids in cell culture (SILAC) and mass spectrometry (MS) analysis. Seven lysine (K) residues of METTL3 were identified as potential acetylation sites: K13, K27, K163, K164, K177, K256, and K263 (Supplementary Fig. 2c). We then substituted each of the seven lysine residues into arginine (R) and found only the K177R mutant failed to be acetylated by p300 (Supplementary Fig. 2d). Consistent with this result, an antibody raised against acetyl-K177 METTL3 specifically recognized the acetylated WT METTL3 in the presence of p300, but not the acetylation-deficient K177R mutant (Fig. 2e and Supplementary Fig. 2e). K177 turns out to be a conserved residue among different species (Supplementary Fig. 2f). Together, these results indicate that K177 is the major acetylation site on METTL3 modified by p300.

We next aimed to identify the potential deacetylase(s) for METTL3. Treating cells expressing HA-p300 with nicotinamide (NAM), an inhibitor of class-III sirtuin deacetylase (SIRT), increased acetylation of ectopic METTL3 (Supplementary Fig. 2g). By contrast, trichostatin A (TSA), a histone deacetylases (HDACs) inhibitor, failed to affect METTL3 acetylation. In vitro deacetylation assay using purified 7 individual sirtuins revealed that METTL3 was only deacetylated by WT-SIRT1, SIRT2, and SIRT3 in the presence of NAD^+^ (Supplementary Fig. 2h), but not by their catalytically inactive mutants (Supplementary Fig. 2i). Consistently, ectopic SIRT1, SIRT2, and SIRT3 displayed robust deacetylation activity towards Myc-METTL3 when co-transfected into HEK293T cells (Supplementary Fig. 2j). However, ectopic METTL3 only interacted with endogenous SIRT1, but not SIRT2 or SIRT3 (Fig. 2f). Furthermore, only SIRT1 knockdown profoundly boosted endogenous METTL3 acetylation (Fig. 2g). Collectively, SIRT1 represents the physiological deacetylase for METTL3.

### Aspirin synergizes with SIRT1 inhibition in inducing K177 acetylation, thereby abrogating METTL3 nuclear translocation

**Fig. 3:**
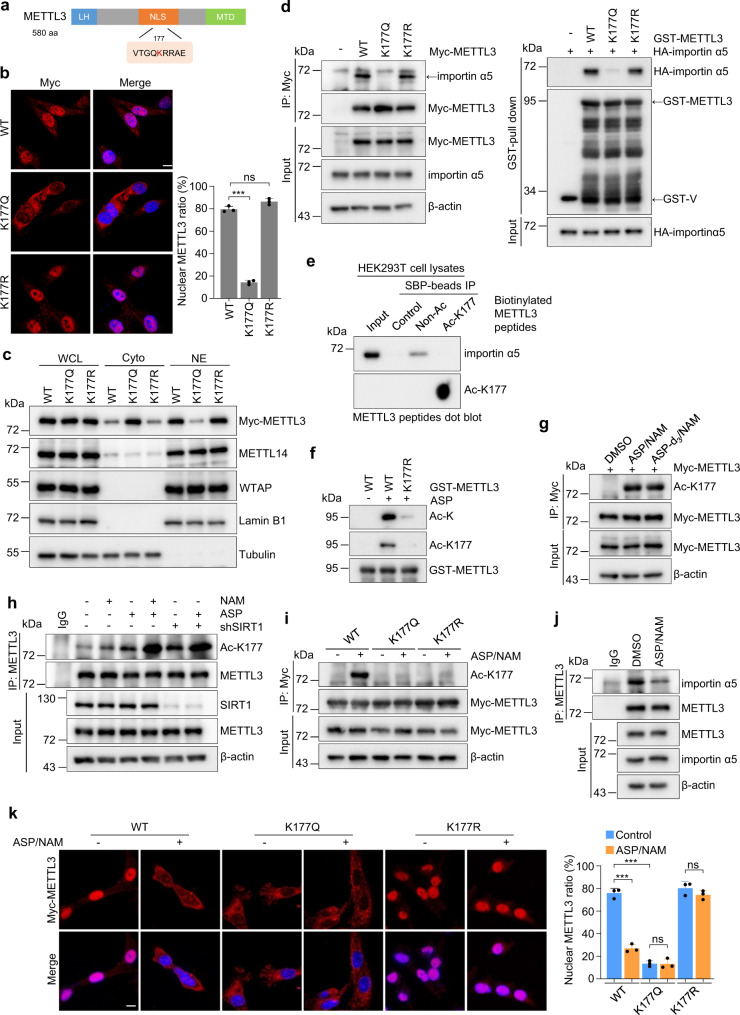
Aspirin synergizes with SIRT1 inhibition in inducing K177 acetylation, thereby abrogating METTL3 nuclear translocation. **a** Schematic representation of METTL3 NLS. **b** Representative immunofluorescence for Myc-METTL3 (red) and DAPI staining (blue, cell nuclei) in METTL3 reconstituting MDA-MB-231 cells (left panel) (*n* = 3 biologically independent experiments). Scale bars, 10 μm. Quantification of nuclear METTL3 percentage is presented as mean ± SD (right panel). ^***^P = 0.001, ns *P* = 0.11, respectively, by two-sided *t* test. **c** IB analysis of whole cell lysate (WCL), cytoplasmic (Cyto) and nuclear (NE) fractions from METTL3 reconstituting MDA-MB-231 cells. **d** Lysates of HEK293T cells transfected with the indicated constructs were subjected to IP and IB analysis (left panel). Lysates of HEK293T cells transfected with HA-importin α5 were incubated with recombinant GST-METTL3. Proteins retained on sepharose were then blotted with the indicated antibodies (right panel). **e** Biotinylated METTL3 peptides containing either acetylated or non-acetylated K177 residue were synthesized and incubated with HEK293T cell lysates. Peptides were then purified with streptavidin sepharose, followed by immunoblotting. Peptides were verified by dot blotting using Ac-K177 antibody. **f** Incubation of the indicated recombinant METTL3 proteins with ASP, followed by IB analysis of the acetylation level of METTL3 with pan-Ac-K or Ac-K177 antibodies. **g** HEK293T cells transfected with Myc-METTL3 were treated with ASP/NAM or isotopically labeled ASP-*d*_*3*_/NAM. Lysates were analyzed by IP and IB analysis as indicated. **h** MDA-MB-231 cells infected with the indicated lentiviruses were treated with DMSO, NAM, ASP, or ASP/NAM. Lysates were analyzed by IP and IB analysis. **i** METTL3 reconstituting MDA-MB-231 cells were treated with ASP/NAM. Lysates were collected and subjected to IP and IB analysis. **j** Lysates from MDA-MB-231 cells treated with ASP/NAM were subjected to IP and IB analysis as indicated. **k** Representative immunofluorescence for Myc-METTL3 (red) and DAPI (blue, cell nuclei) in METTL3 reconstituting MDA-MB-231 cells treated with ASP/NAM (left panel) (*n* = 3 biologically independent experiments). Scale bars, 10 μm. Quantification of nuclear METTL3 percentage is presented as mean ± SD (right panel). From left to right **k**: ^***^P = 1.13e-04, ^***^P = 2.62e-05, ns *P* = 0.95, ns *P* = 0.21, respectively, by two-sided *t* test. In this figure, cells were treated with 5 mM ASP alone or together with 3 mM NAM for 24 h. Source data are provided as a Source Data file.

We noticed that K177 is within the nuclear localization signals (NLSs) of METTL3 (Fig. 3a). It has been suggested that acetylation of lysine residues within an NLS often influences subcellular localization^36,37^. To test this, we performed IF staining and observed enhanced cytoplasmic retention of endogenous METTL3 only in HEK293T cells expressing WT p300 (Supplementary Fig. 3a). To evaluate the direct contribution of K177 acetylation for METTL3 distribution, METTL3-depleted MDA-MB-231 cells were reconstituted with either METTL3^WT^, METTL3^K177Q^ (acetylation-mimetic) or METTL3^K177R^ constructs (Supplementary Fig. 3b). Similar to the distribution pattern of endogenous METTL3, both METTL3^K177R^ and METTL3^WT^ were predominantly nuclear, while METTL3^K177Q^ was largely retained in the cytoplasm (Fig. 3b). Consistent data were obtained in HEK293T cells transiently transfected with GFP-METTL3-WT or GFP-METTL3-K177Q construct (Supplementary Fig. 3c). Expression of K177 mutants did not influence the localization of endogenous METTL14 and WTAP (Fig. 3c). To assess the acetylation levels of METTL3 in different cellular compartments, cytoplasmic and nuclear fraction containing equivalent amount of METTL3 was subjected to immunoprecipitation assay followed by immunoblotting with Ac-K or acetyl-K177 antibody. A great portion of endogenous cytosolic METTL3 was acetylated, whereas acetylation of nuclear METTL3 was barely detectable (Supplementary Fig. 3d), supporting a positive correlation between K177 acetylation and cytoplasmic localization of METTL3.

Since NLS is critical for its recognition by the importin complex, we suspect that the replacement of K177 may abolish METTL3-importin complex formation. Indeed, the K177Q mutant showed very faint interaction with the importin α5 subunit (Fig. 3d), the only importin family member that bound to METTL3 (Supplementary Fig. 3e). METTL3^K177R^ displayed a similar binding affinity with importin α5 both in vitro and in vivo (Fig. 3d). To assess if acetylation of METTL3 may directly affect its recognition by the importin complex, we performed pull-down assays with biotinylated peptides that contain the canonical NLS. Importin α5 only associated with non-acetylated METTL3 in vitro (Fig. 3e). These results indicate that acetylation of METTL3 at K177 within the NLS disrupts its recognition by the importin complex, offering a molecular mechanism for acetylation-dependent regulation of METTL3 localization.

Although SIRT1 was capable of deacetylating METTL3 at K177, we noticed that SIRT1 depletion exerted a relatively mild effect on METTL3 acetylation, which only resulted in a subtle increase of cytosolic METTL3 (Supplementary Fig. 3f). We, therefore, set out to identify intracellular stimuli that could synergize with SIRT1 inhibition in triggering more robust acetylation and the subsequent cytoplasmic retention of METTL3. Aspirin (ASP), a non-steroidal anti-inflammatory drug (NSAID), has been found to directly acetylate the amino group of lysine residue to form an amide bond (N-link)^38^. When recombinant GST-METTL3 was incubated with 5 mM ASP in vitro, only WT METTL3, but not METTL3^K177R^ mutant, displayed a strong acetylation signal (Fig. 3f). Using our site-specific METTL3 acetylation antibodies, we further confirmed that ASP can acetylate recombinant METTL3 at K177 in a dose-dependent manner (Supplementary Fig. 3g). To further ascertain that ASP can directly acetylate METTL3, we used an isotopically labeled ASP (ASP-*d*_*3*_) based on a previous study^39^. Similar to the regular ASP, isotopically labeled ASP can acetylate ectopic METTL3 protein (Fig. 3g). Consistently, ASP-*d*_*3*_ treatment led to a significantly increased METTL3 acetylation with an acetyl-*d*_*3*_ group detected by MS (Supplementary Fig. 3h). These data indicate that ASP treatment can directly induce METTL3 acetylation at K177 in vitro.

To examine if ASP exposure could induce METTL3 acetylation in vivo, we first tested the cytotoxicity of ASP by exposing MDA-MB-231 cells with varying concentrations of ASP. Incubating cells with up to 5 mM ASP (plasma achievable dose range of ASP: 1–5 mM)^40^ for 24 h did not significantly affect cell proliferation (Supplementary Fig. 3i). We next tested if ASP may synergize with SIRT1 inhibition in promoting METTL3 acetylation. Indeed, ASP/NAM co-treatment profoundly increased endogenous METTL3 acetylation, compared to NAM or ASP exposure alone (Fig. 3h). Similar results were obtained in SIRT1-depleted cells treated with ASP. Moreover, in METTL3 reconstituting cells, ASP/NAM induced robust acetylation of METTL3^WT^, but not of METTL3 K177 mutant proteins, suggestive of site-specific acetylation by ASP/NAM (Fig. 3i). Of note, ectopic SIRT1 counteracted ASP/NAM-induced METTL3 acetylation (Supplementary Fig. 3j). Together, these results indicate that ASP could synergize with SIRT1 inhibition in enforcing METTL3 acetylation at K177.

We next assessed if ASP/NAM can influence METTL3 localization. As expected, ASP/NAM treatment markedly abolished endogenous METTL3-importin α5 complex formation (Fig. 3j), reduced nuclear shift of METTL3^WT^ protein, and, consequently, increased the cytosolic pool of METTL3 (Fig. 3k, and Supplementary Fig. 3k). This dynamic redistribution of METTL3 was not observed in METTL3 K177 mutant cells, demonstrating K177-acetylation dependency. We also examined if ASP/NAM exposure may modify other components of METTL3 methyltransferase complex. As shown in Supplementary Fig. 3l, ectopic WTAP or METTL14 failed to show detectable acetylation signals in response to ASP/NAM stimulation. Meanwhile, ASP/NAM failed to change the subcellular localization or expression of the indicated METTL3 subunits and m^6^A demethylases (Fig. 3k and Supplementary Fig. 3m, n). These data highlight the substrate specificity of ASP/NAM in modulating METTL3.

We noticed that the cytosolic/nuclear ratio of METTL14 is always consistent with the ratio of METTL3 across tumor cell lines (Supplementary Fig. 1j, k). This phenomenon reminded us about the reciprocal regulation of protein stability between these two binding partners observed by others^41^. Unfortunately, the underlying molecular mechanisms remain a mystery. Here, it’s rather unexpected that the acute translocation induced by ASP/NAM or K177Q mutation only altered the cytosolic/nuclear ratio of METTL3, without influencing METTL14 accordingly. To explain our observation, we performed the following experiments. (1) We suspect that the regulation of METTL14 turnover by METTL3 might be dose-dependent. To test this, we purified and compared the amount of nuclear METTL3 under different experimental settings. As shown in Supplementary Fig. 3o, levels of nuclear METTL3^K177Q^ in reconstituting cells were about ~20% of levels in METTL3^WT^ cells. A similar result was obtained in MDA-MB-231 cells upon ASP/NAM treatment. Utilizing MDA-MB-231 cells exhibiting different knockdown efficiency of METTL3, we found that the impaired expression of endogenous nuclear METTL14 was only observed when nuclear METTL3 was reduced to 10% of control levels, supporting threshold dependence for METTL3-mediated METTL14 turnover. (2) Given the highly abundant expression of METTL3 in various cancer cell lines, we speculate that there might be significant excess of METTL3 unbound to METTL14, which explains its dose-dependent regulation of METTL14 stability. It’s important to note that METTL14 can bind both METTL3^WT^ and METTL3^K177Q^ with similar binding affinities (Supplementary Fig. 3p). Intriguingly, ASP/NAM-induced alteration of METTL3 abundance failed to change the amount of bound METTL14 in either the cytoplasm or the nucleus (Supplementary Fig. 3q), supportive of the existence of cellular METTL3 pool that is free of METTL14 interaction. In sharp contrast, the reduced nuclear METTL3 abundance profoundly attenuated its association with WTAP, RBM15, KIAA1429, and ZC3H13 (Supplementary Fig. 3r, s). Given ZC3H13-WTAP-KIAA1429 has been identified as an evolutionarily conserved complex^42^, it’s plausible that METTL3 may form distinct complexes with these subunits. Further studies are needed to investigate the assembly kinetics and stoichiometry of the endogenous METTL3-containing complexes, as well as the cooperative binding properties among different subunits.

### ASP/NAM-induced K177 acetylation of METTL3 impedes m^6^A modification

**Fig. 4:**
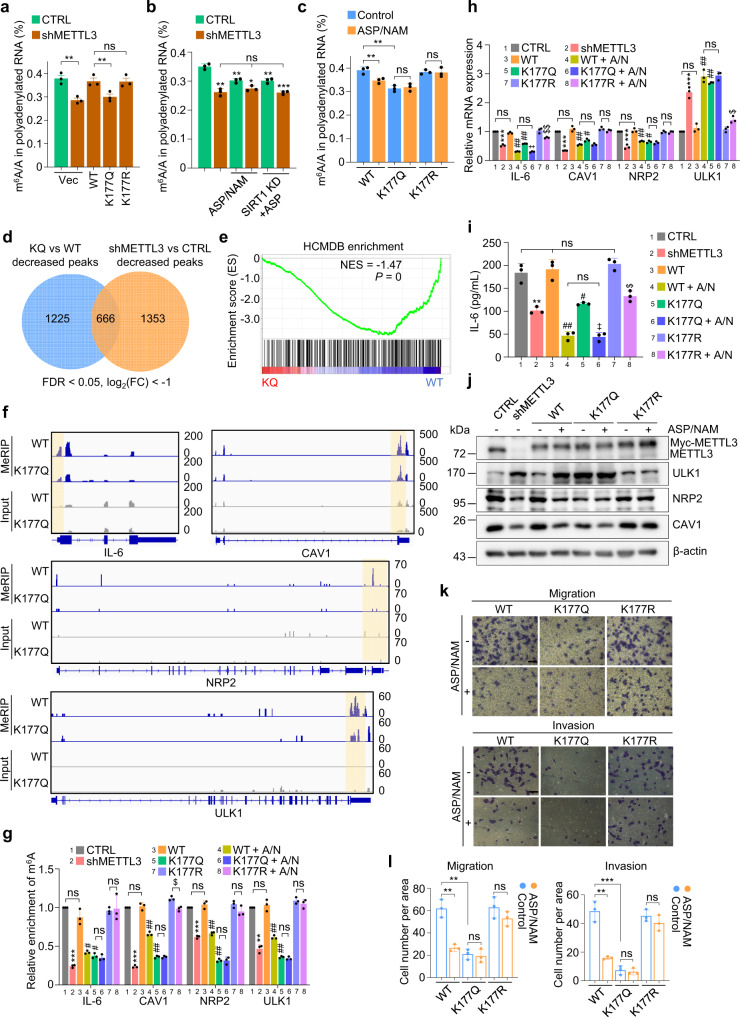
ASP/NAM-induced K177 acetylation of METTL3 impedes m6A modification. **a** LC-MS/MS quantification of the m^6^A/A ratio in polyadenylated RNA isolated from MDA-MB-231 cells infected with the indicated lentiviruses (*n* = 3 biologically independent experiments). From left to right **a**: ^**^P = 0.01, ^**^P = 0.005, ns *P* = 0.24, respectively, by two-sided *t* test. **b** LC-MS/MS quantification of the m^6^A/A ratio in polyadenylated RNA isolated from MDA-MB-231 cells infected with the indicated lentiviruses, or either treated with ASP/NAM or ASP (*n* = 3 biologically independent experiments). From left to right **b**: ^**^P = 0.003, ^**^P = 0.0012, ^*^P = 0.02, ^**^P = 0.0035, ^***^P = 0.0009, ns *P* = 0.40, ns *P* = 0.77, respectively, by two-sided *t* test. **c** LC-MS/MS quantification of the m^6^A/A ratio in polyadenylated RNA isolated from METTL3 reconstituting MDA-MB-231 cells treated with ASP/NAM (*n* = 3 biologically independent experiments). From left to right **c**: ^**^P = 0.0026, ^**^P = 0.0019, ns *P* = 0.54, ns *P* = 0.78, respectively, by two-sided *t* test. **d** Venn diagram showing overlap between differential down-regulated m^6^A peaks in METTL3-deficient and METTL3^K177Q^ reconstituting MDA-MB-231 cells. **e** GSEA of differentially m^6^A-methylated mRNAs in METTL3^WT^ and METTL3^K177Q^ MDA-MB-231 cells compared with pro-metastatic genes associated with breast cancer in HCMDB database. **f** Genomic visualization of the m^6^A-MeRIP-seq normalized signal in METTL3^WT^ and METTL3^K177Q^ MDA-MB-231 cells for the METTL3-dependent m^6^A substrates *IL-6, CAV1, NRP2*, and *ULK1*. Blue, MeRIP; gray, input. **g** m^6^A-MeRIP-qPCR analysis of the indicated m^6^A substrates normalized to input in METTL3 reconstituting MDA-MB-231 cells subjected to the indicated treatment (*n* = 3 biologically independent experiments). From left to right for IL-6: ^***^P = 2.92e-07, ns *P* = 0.1, ^#^*P* = 0.0016, ^#^*P* = 0.0012, ns *P* = 0.46, ns *P* = 0.81, respectively, by two-sided *t* test. From left to right for CAV1: ^***^P = 1.73e-07, ns *P* = 0.57, ^##^*P* = 3.09e-04, ^##^*P* = 2.11e-05, ns *P* = 0.92, ^$^*P* = 0.005, respectively, by two-sided *t* test. From left to right for NRP2: ^***^P = 4.24e-06, ns *P* = 0.25, ^##^*P* = 2.8e-04, ^##^*P* = 2.06e-05, ns *P* = 0.96, ns *P* = 0.11, respectively, by two-sided *t* test. From left to right for ULK1: ^**^P = 5.39e-05, ns *P* = 0.50, ^##^*P* = 7.92e-04, ^##^*P* = 1.09e-04, ns *P* = 0.30, ns *P* = 0.40, respectively, by two-sided *t* test. **h** QRT-PCR quantification of the indicated mRNAs in METTL3 reconstituting MDA-MB-231 cells (*n* = 3 biologically independent experiments). From left to right for IL-6: ^***^P = 2.07e-05, ns *P* = 0.0503, ^##^*P* = 3.42e-06, ^##^*P* = 3.01e-05, ns *P* = 0.56, ^‡^*P* = 2.58e-05, ^$$^*P* = 0.0082, respectively, by two-sided *t* test. From left to right for CAV1: ^***^P = 4.21e-10, ns *P* = 0.15, ^##^*P* = 5.32e-04, ^#^*P* = 0.0028, ns *P* = 0.79, ns *P* = 0.17, respectively, by two-sided *t* test. From left to right for NRP2: ^***^P = 2.55e-04, ns *P* = 0.42, ^##^*P* = 8.47e-04, ^#^*P* = 0.0011, ns *P* = 0.16, ns *P* = 0.85, respectively, by two-sided *t* test. From left to right for ULK1: ^***^P = 2.24e-04, ns *P* = 0.21, ^##^*P* = 2.76e-04, ^##^*P* = 4.80e-05, ns *P* = 0.85, ^$^*P* = 0.018, respectively, by two-sided *t* test. **i** Measurement of IL-6 levels by ELISA in METTL3 reconstituting MDA-MB-231 cells subjected to the indicated treatment (*n* = 3 biologically independent experiments). From left to right **i**: ^**^P = 0.0027, ns *P* = 0.67, ^##^*P* = 4.03e-04, ^#^*P* = 0.0036, ns *P* = 0.77, ^‡^*P* = 2.0e-04, ^$^*P* = 0.0022, respectively, by two-sided *t* test. For *P* values in **g**, **h**, and **i**, ^*^compared to CTRL; ^#^compared to METTL3^WT^; ^‡^compared to METTL3^K177Q^; ^$^Compared to METTL3^K177R^. **j** IB analysis of the indicated proteins in METTL3 reconstituting MDA-MB-231 cells. **k, l** Migration and invasion assays were conducted using METTL3 reconstituting MDA-MB-231 cells treated with ASP/NAM (**k**) (*n* = 3 biologically independent experiments). Scale bars, 50 μm. Cells were treated with 5 mM ASP and/or 3 mM NAM for 72 h. Quantification is presented in (**l**). From left to right for migration: ^**^P = 0.002, ^**^P = 0.0015, ns *P* = 0.72, ns *P* = 0.21, respectively, by two-sided *t* test. From left to right for invasion: ^**^P = 0.0014, ^***^P = 0.0008, ns *P* = 0.67, ns *P* = 0.28, respectively, by two-sided *t* test. All data are represented as mean ± SD. All *P* values were calculated by Student’s *t* test. Source data are provided as a Source Data file.

The impaired nuclear retention of METTL3 resulting from its enhanced acetylation prompted us to examine the influence of METTL3 acetylation on m^6^A deposition. Loss of METTL3 in MDA-MB-231 cells caused significantly reduced m^6^A abundance measured by LC-MS/MS, which can be rescued by METTL3^WT^ and METTL3^K177R^ reconstitution, but not by METTL3^K177Q^ complementation (Fig. 4a). In vitro methylation assay utilizing recombinant METTL3 proteins purified from a baculovirus expression system revealed comparable methyltransferase activities between WT and K177 mutant METTL3 proteins (Supplementary Fig. 4a), indicating that K177 mutations do not affect the catalytic function of METTL3. This was further supported by our data showing METTL3^K177Q/CD^ reconstituting cells displayed compromised m^6^A modification similar to that of METTL3^CD^ cells (Supplementary Fig. 4b).

We next assessed if ASP/NAM may regulate m^6^A dynamics in vivo. Treating MDA-MB-231 cells with ASP/NAM profoundly impaired m^6^A levels in a METTL3-dependent manner, albeit to a lesser extent compared to METTL3 depletion (Fig. 4b). Notably, ASP/NAM-mediated m^6^A reduction occurred in additional tumor cell lines following ASP/NAM treatment (Supplementary Fig. 4c), which correlated with enhanced METTL3 acetylation (Supplementary Fig. 4d). Importantly, as shown in Fig. 4c, ASP/NAM only reduced m^6^A levels in METTL3^WT^, but not in K177 mutant reconstituting cells. Of note, the basal m^6^A abundance in METTL3^K177Q^ cells was significantly lower, compared to METTL3^WT^ cells. Similar results were obtained using reconstituting cells treated with ASP and SIRT1 depletion (Supplementary Fig. 4e). We noticed that prolonged exposure (72 h) with 5 mM ASP reduced cell viability in METTL3^WT^ cells. However, similar proliferation-inhibition phenotypes were observed in METTL3^K177Q^ cells, excluding the possibility that ASP/NAM-mediated m^6^A deduction was secondary to the impaired cell survival rate (Supplementary Fig. 4f).

To gain further insights into how acetylation of METTL3 may affect the m^6^A-modified transcripts and the subsequent impact on global transcriptome, we mapped the m^6^A methylome in METTL3 reconstituting cells as well as in METTL3-deficient cells. Differential m^6^A peaks were consistent between two biological replicates, indicating good reproducibility (Supplementary Fig. 4g). Comparison of m^6^A methylome between METTL3^WT^ and METTL3^K177Q^ reconstituting cells revealed the following findings: (1) Significant global alteration of methylation sites due to K177Q mutation (termed as KQ-m^6^A signature) (Supplementary Fig. 4h). The identified m^6^A peaks in our study were characterized by the canonical GGACU motifs (Supplementary Fig. 4i). (2) Gene Ontology (GO) analysis of the differential m^6^A-MeRIP candidates enriched pathways involved in chromatin modification, RNA splicing, DNA damage, cell cycle, and autophagy (Supplementary Fig. 4j), consistent with a critical role of m^6^A-mediated nuclear biological activities and highlighted generally recognized cellular events associated with tumorigenesis.

We next compared our KQ-m^6^A signature to m^6^A-MeRIP-seq data obtained from METTL3 knockdown cells. As expected, METTL3 depletion resulted in profoundly reduced m^6^A peaks, which we termed as M3-KD (METTL3 knockdown)-m^6^A signature (Supplementary Fig. 4k). Notably, the general m^6^A distribution, which were enriched near the start and stop codons, showed no major differences among the indicated samples (Supplementary Fig. 4l). When compared KQ-m^6^A with M3-KD-m^6^A signature, we found a significant overlap of reduced m^6^A peaks (Fig. 4d). The substrate and peak differences observed between these two groups could be attributed to the fact that, unlike METTL3 depletion, which simply blunted both nuclear and cytosolic functions of METTL3, K177Q only attenuated its nuclear functions but enhanced the cytosolic functions (see “Discussion”). Given the crucial role of METTL3 in metastasis, which relied primarily on its methyltransferase activity, we suspect that METTL3^K177Q^ might be incompetent at promoting migration/invasion. Indeed, unlike METTL3^WT^ and METTL3^K177R^ reconstitution, which exerted complete rescue effects on defective migration/invasion due to METTL3 loss, METTL3^K177Q^ failed to restore these phenotypes (Supplementary Fig. 4m, n), demonstrating this acetylation-mimetic mutation blocked the nuclear function of METTL3, leading to compromised invasiveness of breast cancer cells.

To dissect potential mechanistic clues explaining the attenuated invasive phenotype in METTL3^K177Q^ reconstituting cells, we focused on genes known to modulate metastasis and their transcripts showing differential m^6^A methylation in our m^6^A MeRIP-seq analysis. To this end, we utilized gene hits from Human Cancer Metastasis Database (HCMDB)^43^ (Supplementary Table 1), and compared them with KQ-m^6^A signature genes for GSEA. The 487 known metastasis-promoting gene hits from HCMDB were significantly enriched in METTL3^WT^ cells, but not in METTL3^K177Q^ cells (Fig. 4e). We selected m^6^A-methylayed poly(A)^+^ transcripts with reduced m^6^A peaks from KQ-m^6^A signature dataset, and further filtered them based on RNA-seq results. The gene hits showed reduced expression in KQ cells (FC > 1.5) were overlapped with HCMDB dataset. Using these criteria, K177Q mutation resulted in a significantly reduced expression of 26 metastasis-promoting transcripts (Supplementary Table 2), including *IL-6*, *NRP2*, and *CAV1*, three well-known metastasis-promoting genes (Fig. 4f). Since m^6^A methylation can also decrease RNA stability, we examined KQ-m^6^A signature genes that exhibited less m^6^A methylation but with elevated transcript expression. *ULK1*, a well-known metastasis-repressing gene, was up-regulated in KQ cells (Fig. 4f). Validating m^6^A methylation of these selected transcripts revealed significant decrease of m^6^A levels in both METTL3^K177Q^ and METTL3-depleted cells (Fig. 4g). Their RNA and protein expression levels were further verified accordingly (Fig. 4h–j). As shown in Supplementary Fig. 4o, K177Q-induced change in mRNA stability of the selected genes displayed similar pattern to their altered transcript abundance. We went on elucidating mechanisms underlying the altered stabilities of the indicated transcripts. Silencing YTHDF2 induced ULK1 expression, whereas IGF2BP3 depletion displayed robust suppression of both IL-6 and NRP2. IGF2BP1 knockdown repressed CAV1 expression (Supplementary Fig. 4p, q). Further evaluation of their mRNA turnover rates revealed consistent alteration pattern (Supplementary Fig. 4r). By manipulating their expression levels in METTL3^K177Q^ cells, we evaluated and confirmed the functional importance of these selected mRNA substrates in mediating METTL3-dependent invasiveness (Supplementary Fig. 4s–u).

We noticed that in METTL3^WT^ cells, ASP/NAM treatment led to profoundly reduced m^6^A methylation on the selected transcripts, resulting in the downregulation of metastasis-promoting genes and upregulation of ULK1, respectively (Fig. 4g–j). By contrast, K177R and K177Q cells conferred significant resistance to ASP/NAM-mediated suppression of m^6^A methylation, and changes of selected targets at both transcription and translation levels. Similar results were obtained in METTL3^CD^ reconstituting cells, indicating the enzymatic activity of METTL3 is indispensable (Supplementary Fig. 4v–x). As shown in Supplementary Fig. 4y, z, aa, ab, compared to METTL3 depletion alone, ASP/NAM treatment in METTL3-deficient cells failed to further alter either m^6^A deposition, or mRNA/protein levels of the indicated genes, suggesting METTL3 dependency. Interestingly, K177 mutations rendered reconstituting cells partially sensitive to ASP/NAM-triggered inhibition of IL-6 expression (Fig. 4h, i). Similarly, METTL3^CD^ cells and METTL3-deficient cells, which showed significantly reduced basal IL-6 levels (Supplementary Fig. 4v, x, z, aa), still partially responded to ASP/NAM-mediated repression of IL-6 expression. It’s plausible that ASP/NAM can regulate IL-6 expression independently of METTL3 signaling via alternative mechanisms. For instances, a previous study reported ASP-mediated suppression of *IL-6* transcription via NF-κB^44^. Nevertheless, ASP/NAM-mediated repression of migration/invasion only occurred in METTL3^WT^ cells, but not in K177 mutant cells or METTL3-deficient cells (Fig. 4k, l and Supplementary Fig. 4ac). Taken all together, METTL3 acetylation triggered by ASP/NAM treatment elicited a suppressive effect on invasiveness of tumor cells via impacting on both pro-metastasis and anti-metastasis pathways in an m^6^A-dependent manner.

### Acetylation-mimetic METTL3^K177Q^ reconstitution enhances protein translation

**Fig. 5:**
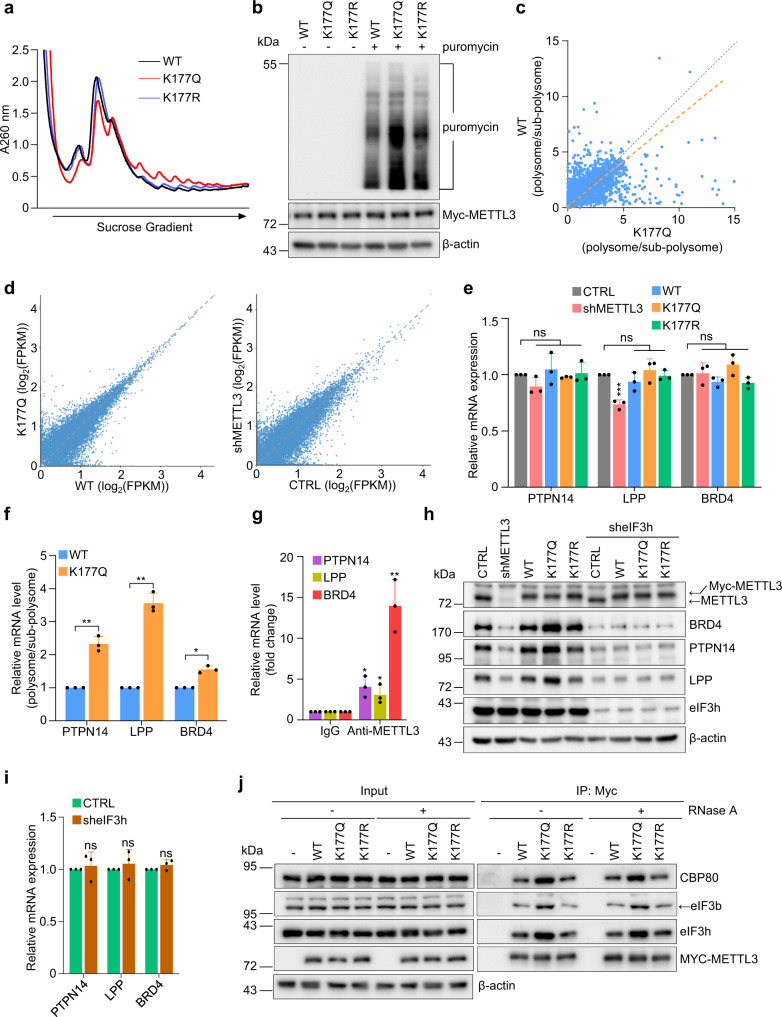
Acetylation-mimetic METTL3K177Q reconstitution enhances protein translation. **a** Polysome fractionation analysis in METTL3 reconstituting MDA-MB-231 cells. Absorbance was continuously measured at 260 nm. Representative profile of two independently performed experiments with similar results. **b** SUnSET assays show enhanced protein production in METTL3^K177Q^ reconstituting MDA-MB-231 cells. Cell lysates were extracted and probed the indicated antibodies. **c** Scatter plot of translation efficiency. The read number from the indicated reconstituting cells was calculated by the ratio of the polysome fraction to the read number in the sub-polysome fraction (40/60/80 S). **d** Scatter plot of RNA-seq data. The average read number from METTL3^WT^ versus METTL3^K177Q^ MDA-MB-231 cells (left panel), and average read number from CTRL versus METTL3-deficient MDA-MB-231 cells (right panel) are shown. **e** Relative levels of the indicated mRNAs in METTL3 reconstituting MDA-MB-231 cells measured by QRT-PCR analysis (*n* = 3 biologically independent experiments). From left to right for PTPN14: ns *P* = 0.084, ns *P* = 0.45, ns *P* = 0.75, respectively, by two-sided *t* test. From left to right for LPP: ^***^P = 0.0004, ns *P* = 0.24, ns *P* = 0.38, respectively, by two-sided *t* test. From left to right for BRD4: ns *P* = 0.81, ns *P* = 0.06, ns *P* = 0.80, respectively, by two-sided *t* test. **f** QRT-PCR quantification of the indicated mRNAs in polysome fractions presented as the ratio to the relative mRNA levels in sub-polysome fractions (*n* = 3 biologically independent experiments). From left to right: ^**^P = 0.0099, ^**^P = 0.0047, ^*^P = 0.012, respectively, by two-sided *t* test. **g** QRT-PCR quantification of METTL3-associated mRNAs in MDA-MB-231 cells (*n* = 3 biologically independent experiments). From left to right: ^*^P = 0.015, ^*^P = 0.037, ^**^P = 0.0022, respectively, by two-sided *t* test. **h** Lysates of METTL3 reconstituting MDA-MB-231 cells infected with the indicated lentiviruses were subjected to IB analysis. **i** Relative mRNA levels of the indicated mRNAs in MDA-MB-231 cells infected with the indicated lentiviruses were analyzed by QRT-PCR (*n* = 3 biologically independent experiments). From left to right: ns *P* = 0.68, ns *P* = 0.53, ns *P* = 0.21, respectively, by two-sided *t* test. **j** Lysates of METTL3 reconstituting MDA-MB-231 cells were treated with RNase A, followed by IP and IB analysis. All data are represented as mean ± SD. All *P* values were calculated by Student’s *t* test. Source data are provided as a Source Data file.

It has been suggested that cytosolic METTL3 can regulate mRNA translation independent of its methyltransferase function, but relying on mRNA circularization via interacting with eIF3h^28^. Using sucrose gradient centrifugation, we observed an increase in the abundance of 80 S ribosomes as well as 40 S and 60 S subunits, and a decrease in polysomes in MDA-MB-231 cells depleted of METTL3 relative to control cells (Supplementary Fig. 5a). By contrast, polysome profiles in METTL3^K177Q^ cells showed an increase in polysome peak with concomitant reductions in 40 S, 60 S, and 80 S ribosome fractions, indicating METTL3^K177Q^ may potentiate mRNA translation (Fig. 5a).

We next measured protein synthesis in MDA-MB-231 reconstituting cells using surface sensing of translation (SUnSET), a method for monitoring global protein synthesis through detection of puromycin-labeled neosynthesized proteins^45^. Compared to METTL3^WT^ or METTL3^K177R^, METTL3^K177Q^ reconstitution remarkably enhanced global protein synthesis (Fig. 5b), whereas METTL3 deficiency profoundly suppressed this process (Supplementary Fig. 5b), further supporting a translation-promoting role of METTL3 ^K177Q^. It is worth pointing out that METTL3^K177Q/CD^ behaved similarly to METTL3^K177Q^ in boosting protein translation, suggesting cytosolic METTL3-triggered translation is independent of its catalytic function (Supplementary Fig. 5c).

To characterize the impact of K177 acetylation on translatomes, we conducted polysome-profiling experiments on extracts from METTL3^WT^ and METTL3^K177Q^ reconstituting cells (Supplementary Fig. 5d). A substantial amount of efficiently translated mRNAs (*n* = 882) from METTL3^K177Q^ cells was increased by at least 1.5-fold, compared to METTL3^WT^ cells (Fig. 5c). Given the overall steady-state mRNA abundance between these two groups displayed negligible differences (Fig. 5d), these data implied an increased translational efficiency in cells bearing K177Q mutation. 882 gene hits were further compared with previously reported METTL3 photoactivatable ribonucleotide-enhanced crosslinking and immunoprecipitation (PAR-CLIP) data from Hela cells to obtain bona fide translational substrates of METTL3 across human tumor cell lines^22^. A total of 158 transcripts were identified as METTL3-bound and -regulated at the translational level (Supplementary Fig. 5e). To avoid the possibility that the enhanced translation of these 158 genes might be secondary to their increased mRNA abundance, we then filtered them by excluding genes showing >1.0-fold induction based on RNA-seq results. Among the remaining 60 candidates (Supplementary Table 3), we validated 3 transcripts: *PTPN14* and *LPP* (LIM domain containing preferred translocation partner in lipoma), two genes that have been reported as metastatic suppressors^46,47^, and BRD4 (bromodomain-containing protein 4), which has been previously found as a METTL3-dependent translational substrate^28^.

m^6^A deposition on these mRNAs showed no significant alterations between K177Q and WT cells (Supplementary Fig. 5f), suggesting m^6^A modification is dispensable for their translation process. QRT-PCR analysis of the selected targets revealed that METTL3^K177Q^ reconstitution had an either a very subtle or undetectable effect on their mRNA abundance (Fig. 5e), but strongly promoted mRNA translation (Fig. 5f). To ensure that METTL3 is bound to polysomes, mRNA ribonucleoprotein complexes (mRNPs) containing endogenous METTL3 were purified from MDA-MB-231 cells and the association of METTL3 with these substrate mRNAs were confirmed by QRT-PCR (Fig. 5g). Consistently, reduced expression of BRD4, PTPN14 and LPP protein levels were detected when METTL3 was depleted, which can be restored by either METTL3^WT^ or METTL3 K177 mutants reconstitution (Fig. 5h). Of note, protein expression of these candidate genes reached much higher levels in METTL3^K177Q^ cells, demonstrating METTL3^K177Q^ can facilitate translational efficiency. To further investigate their potential contribution in mediating KQ-dependent suppression of migration/invasion, we depleted PTPN14 or LPP in our reconstituting cells. As shown in Supplementary Fig. 5g–i, in METTL3^WT^ cells, silencing PTPN14 or LPP further enhanced migration/invasion, consistent with their metastasis-repressing role. Remarkably, silencing either one significantly yet partially restored migration/invasion potential in METTL3^KQ^ cells.

It has been suggested that the METTL3-eIF3h interaction is required for enhanced translation^28^. Indeed, immunoblotting results showed strongly reduced endogenous protein expression of candidate genes upon knockdown of eIF3h in all reconstituting cells, without affecting their mRNA abundance (Fig. 5h, i). Since METTL3^K177Q^ cells displayed increased cytosolic localization, we speculated that the association between METTL3^K177Q^ and the translation initiation factors should be increased. As expected, greater levels of endogenous CBP80, eIF3b and eIF3h were detected following immunoprecipitation of Myc-METTL3^K177Q^ from reconstituting cells (Fig. 5j). Notably, WT and mutant METTL3 recombinant proteins exhibited similar binding affinity with eIF3h in vitro (Supplementary Fig. 5j). Taken together, the acetylation-mimetic K177Q mutation, by accumulating in the cytosol, may facilitate protein translation via forming complex with the translation initiation complex.

### IL-6 induces a feedback activation of METTL3 nuclear functions via promoting its deacetylation

**Fig. 6:**
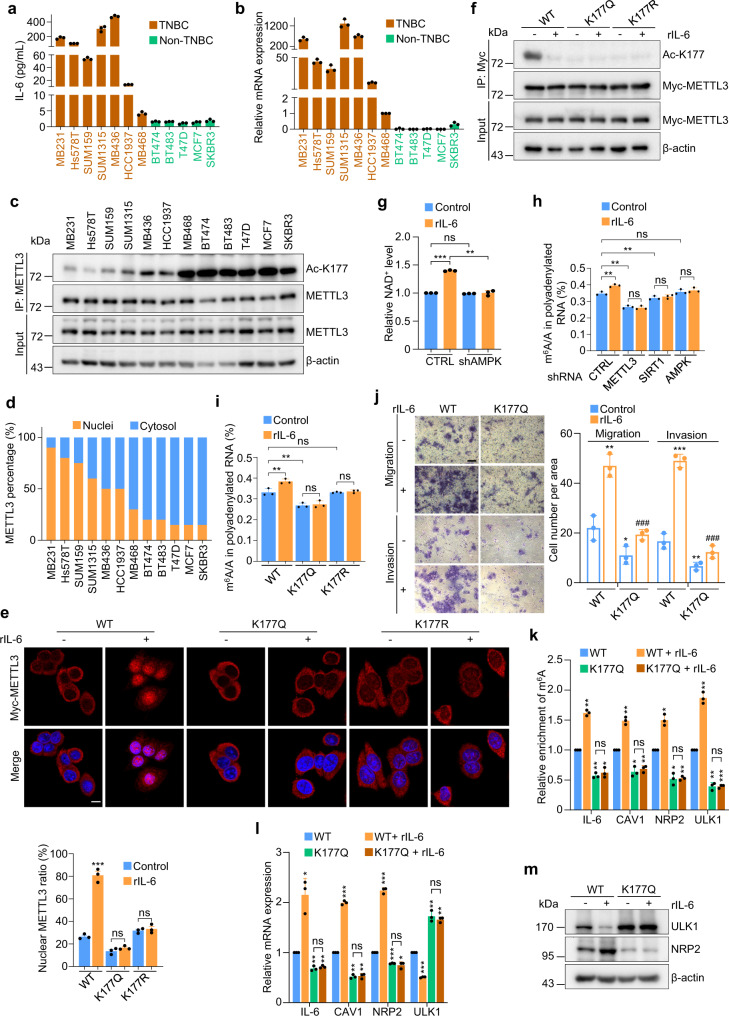
IL-6 induces a feedback activation of METTL3 nuclear functions via promoting its deacetylation. **a** IL-6 levels from the supernatants of the indicated breast cancer cell lines were measured by ELISA (*n* = 3 biologically independent experiments). **b** QRT-PCR quantification of IL-6 mRNA levels in the indicated breast cancer cell lines (*n* = 3 biologically independent experiments). **c** IP and IB analysis of acetyl-K177 METTL3 levels in the indicated breast cancer cell lines. **d** Based on results from Supplemental Fig. 6a, METTL3 protein levels in each fraction were quantified, the percentage of nuclear or cytosolic METTL3 to total METTL3 levels in WCL was calculated. **e** Representative immunofluorescence for Myc-METTL3 (red) and DAPI (blue, cell nuclei) in METTL3 reconstituting MCF-7 cells treated with rIL-6 (upper panel) (*n* = 3 biologically independent experiments). Scale bars, 10 μm. Quantification of nuclear METTL3 percentage is presented as mean ± SD (lower panel). From left to right: ^***^P = 0.0001, ns *P* = 0.19, ns *P* = 0.59, respectively, by two-sided *t* test. **f** Lysates of METTL3 reconstituting MCF-7 cells treated with rIL-6 for 12 h were subjected to IP and IB analysis. **g** Relative NAD^+^ levels were measured by HPLC-MS in MCF-7 cells infected with the indicated lentiviruses and treated with rIL-6 for 1 h (*n* = 3 biologically independent experiments). From left to right: ^***^P = 0.0003, ns *P* = 0.0543, ^**^P = 0.0068, respectively, by two-sided *t* test. **h** LC-MS/MS quantification of the m^6^A/A ratio in polyadenylated RNA isolated from the indicated MCF-7 cells with rIL-6 treatment for 24 h (*n* = 3 biologically independent experiments). From left to right: ^**^P = 0.0059, ^**^P = 0.0019, ns *P* = 0.29, ^**^P = 0.0035, ns *P* = 0.20, ns *P* = 0.085, ns *P* = 0.061, respectively, by two-sided *t* test. **i** LC-MS/MS quantification of the m^6^A/A ratio in polyadenylated RNA isolated from METTL3 reconstituting MCF-7 cells treated with rIL-6 for 24 hours (*n* = 3 biologically independent experiments). From left to right: ^**^P = 0.0035, ^**^P = 0.0029, ns *P* = 0.33, ns *P* = 0.97, ns *P* = 0.26, respectively, by two-sided *t* test. **j** Migration and invasion assays were conducted in METTL3 reconstituting MCF-7 cells treated with rIL-6 (left panel) (*n* = 3 biologically independent experiments). Scale bars, 50 μm. Quantification is presented on the right panel. From left to right: ^**^P = 0.0031, ^*^P = 0.037, ^###^*P* = 0.0007, ^***^P = 0.0002, ^**^P = 0.0071, ^###^*P* = 6.41e-05, respectively, by two-sided *t* test. ^*^compared to WT. ^#^compared to WT + rIL-6. **k** m^6^A-MeRIP-qPCR analysis of the indicated m^6^A substrates normalized to input in METTL3 reconstituting MCF-7 cells subjected to rIL-6 treatment for 24 h (*n* = 3 biologically independent experiments). From left to right: ^**^P = 0.0019, ^**^P = 0.0034, ^*^P = 0.0156, ns *P* = 0.23, ^**^P = 0.0053, ^*^P = 0.017, ^**^P = 0.01, ns *P* = 0.22, ^*^P = 0.012, ^*^P = 0.013, ^**^P = 0.0017, ns *P* = 0.80, ^**^P = 0.004, ^**^P = 0.0035, ^***^P = 0.0007, ns *P* = 0.95, respectively, by two-sided *t* test. **l** QRT-PCR quantification of the indicated mRNAs in METTL3 reconstituting MCF-7 cells treated with rIL-6 for 24 h (*n* = 3 biologically independent experiments). From left to right: ^*^P = 0.026, ^**^P = 0.0073, ^**^P = 0.0028, ns *P* = 0.16, ^***^P = 0.0005, ^**^P = 0.0022, ^**^P = 0.0037, ns *P* = 0.60, ^***^P = 0.0007, ^***^P = 0.0006, ^*^P = 0.0205, ns *P* = 0.43, ^***^P = 0.0002, ^**^P = 0.0072, ^**^P = 0.0021, ns *P* = 0.40, respectively, by two-sided *t* test. **m** Lysates of METTL3 reconstituting MCF-7 cells treated with rIL-6 for 24 h were subjected to IB analysis. All data are represented as mean ± SD. All *P* values were calculated by Student’s *t* test. Source data are provided as a Source Data file.

IL-6 is an essential player in both oncogenic and immune responses. The finding that IL-6 mRNA underwent m^6^A modification prompted us to examine if IL-6 production positively correlated with nuclear METTL3 abundance in a panel of human breast cancer cell lines. 6 out of 12 breast cancer cell lines had IL-6 at concentrations between 13–500 pg/mL in their supernatants detected by ELISA (Fig. 6a). In sharp contrast, the secreted IL-6 concentrations in MB468, BT-474, BT-483, T47D, MCF-7, and SKBR3 cell lines were less than 3 pg/mL. Consistently, differential expression of *IL-6* transcripts was observed across breast cancer lines (Fig. 6b). Intriguingly, an inverse correlation between acetyl-K177 level and IL-6 expression was found among these cell lines (Fig. 6c). We next performed fractionation experiment, and normalized nuclear or cytosolic METTL3 to its levels in whole cell lysate (WCL), followed by calculating the relative nucleus/cytosol (N/C) ratio (Supplementary Fig. 6a). It turned out IL-6-high cells had N/C METTL3 ≥ 1, whereas IL-6-low cells showed predominant cytosolic METTL3 (C/N METTL3 ≥ 2) (Fig. 6d).

The above evidence prompted us to test if IL-6 may in fact promote the nuclear enrichment of METTL3. We performed METTL3 depletion and reconstitution in MCF-7 cell line (Supplementary Fig. 6b), which was IL-6-low, and showed over 80% cytoplasmic METTL3. Consistent with endogenous METTL3 localization pattern, METTL3^WT^ and METTL3^K177R^ localized predominantly in the cytoplasm, only a small portion was detected in the nucleus (Supplementary Fig. 6c). In sharp contrast, METTL3^K177Q^ was exclusively in the cytoplasm. Recombinant IL-6 (rIL-6) treatment elicited a significant translocation of METTL3 from the cytoplasm to the nucleus in METTL3^WT^ cells (Fig. 6e and Supplementary Fig. 6d), accompanied by a profound reduction of acetyl-METTL3 levels (Fig. 6f). However, in K177 mutant reconstituting cells, rIL-6 exposure failed to change the subcellular localization of K177Q or K177R proteins. It is important to note that acute rIL-6 treatment had no obvious effect on WTAP and METTL14 subcellular localization. By depleting IL-6R, we further confirmed that the redistribution of endogenous METTL3 was strictly dependent on intact IL-6R signaling (Supplementary Fig. 6e). Translocation of endogenous METTL3 upon rIL-6 exposure was also observed in RKO and LNCaP cells exhibiting predominant cytosolic METTL3 (Supplementary Fig. 6f).

IL-6 has been previously shown to activate AMPK at Thr172^48,49^. AMPK can promote SIRT1 activation via increasing cellular NAD^+^ levels^50^. Given the cellular SIRT1 predominantly localized in the cytosol (Supplementary Fig. 1k), we reasoned that IL-6 may activate SIRT1 via AMPK-mediated upregulation of NAD^+^, leading to deacetylation and translocation of cytosolic METTL3. Indeed, NAD^+^ levels were significantly increased following 1 h of rIL-6 treatment in an AMPK-dependent manner (Fig. 6g). Accordingly, cellular m^6^A levels remarkably increased upon rIL-6 treatment (Fig. 6h). However, in METTL3-depleted cells, rIL-6 exposure failed to induce m^6^A. Likewise, SIRT1 or AMPK deficiency compromised rIL-6-mediated deacetylation and, nuclear translocation of METTL3, which in turn compromised m^6^A accumulation (Fig. 6h and Supplementary Fig. 6g, h), demonstrating the importance of AMPK-SIRT1 axis in mediating IL-6-dependent regulation of METTL3 signaling. In line with these data, K177Q or K177R reconstituting cells conferred resistance to rIL-6-induced m^6^A upregulation (Fig. 6i), further emphasizing that IL-6-induced deacetylation at K177 is pivotal for its impact on m^6^A modification.

IL-6 is known to promote metastasis via multiple signaling pathways. We were curious if IL-6-mediated METTL3 acetylation contributes to tumor invasiveness. As shown in Supplementary Fig. 6i, METTL3 depletion profoundly blunted migration/invasion of MCF-7 cells treated with rIL-6, indicative of METTL3 dependency. Strikingly, METTL3^WT^ cells exhibited significantly enhanced migration/invasion upon rIL-6 exposure (Fig. 6j), whereas K177Q cells conferred drastic resistance to rIL-6 administration, suggesting IL-6-mediated METTL3 deacetylation at K177 contributes to IL-6-induced invasiveness.

We next examined the impact of IL-6 exposure on m^6^A-modified mRNA transcripts from our m^6^A-MeRIP-seq analysis. Significantly enhanced m^6^A methylation within *IL-6, NRP2, CAV1,* and *ULK1* genes upon rIL-6 treatment only occurred in METTL3^WT^ cells, but not in METTL3^K177Q^ cells (Fig. 6k). QRT-PCR analysis revealed downregulation of *ULK1* and upregulation of *IL-6*, *NRP2,* and *CAV1* following rIL-6 stimulation in METTL3^WT^ cells, but not in K177Q cells (Fig. 6l). Protein abundances of ULK1, NRP2 were consistent with their RNA expression patterns (Fig. 6m). Consistent with the previous report, CAV1 protein levels is undetectable in MCF-7 cells^51^. Meanwhile, METTL3 silencing significantly reversed rIL-6-mediated m^6^A modification (Supplementary Fig. 6j), alteration of the indicated mRNA/protein expression (Supplementary Fig. 6k, l). Similar negative results were obtained in METTL3^CD^ reconstituting cells treated with rIL-6, highlighting the dependence of METTL3 catalytic activity (Supplementary Fig. 6m, n). These data suggest that rIL-6-mediated changes of expression in these selected genes are dependent on METTL3. We also checked the functions of NRP2 and ULK1 in mediating METTL3 deacetylation-induced metastasis in MCF-7 cells treated with rIL-6. As shown in Supplementary Fig. 6o–q, silencing NRP2 or ectopic expressing ULK1 significantly yet partially reversed rIL-6-triggered migration/invasion phenotypes, suggesting a crucial role of these m^6^A targets in mediating rIL-6-induced invasiveness via deacetylating METTL3. Collectively, our data revealed that IL-6, via eliciting a positive feedback activation of nuclear METTL3 function, may exploit RNA modification machinery to impact on global transcriptome, promoting oncogenic progression.

### ASP/NAM suppresses tumor metastasis via promoting METTL3 acetylation and fine-tuning the nuclear and cytosolic functions of METTL3

**Fig. 7:**
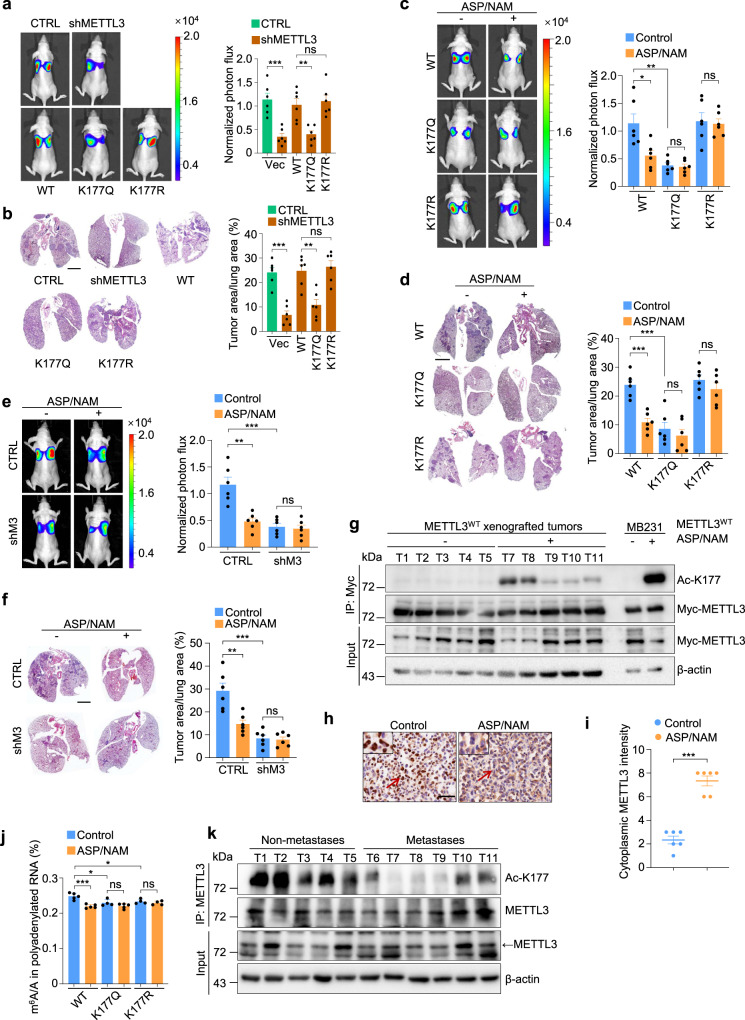
ASP/NAM suppresses tumor metastasis via promoting METTL3 acetylation and fine-tuning the nuclear and cytosolic functions of METTL3. **a** LM2 cells infected with the indicated lentiviruses were injected into the mammary fat pads of nude mice. Representative bioluminescent images (BLI) of mice with spontaneous lung metastasis (left panel), and quantification of BLI (right panel) are shown. *n* = 6 mice per group. From left to right: ^***^P = 0.0004, ^**^P = 0.002, ns *P* = 0.69, respectively, by two-sided *t* test. **b** Representative H&E staining (left panel) and quantification (right panel) analysis of lung metastasis in **a**. *n* = 6 mice per group. Scale bars, 2 mm. From left to right: ^***^P = 5.94e-05, ^**^P = 0.0019, ns *P* = 0.64, respectively, by two-sided *t* test. **c** METTL3 reconstituting LM2 cells were injected into the mammary fat pads of nude mice. Representative BLI of mice with spontaneous lung metastasis following daily treatment with ASP/NAM for 30 days (left panel), and quantification of BLI (right panel) are shown. *n* = 6 mice per group. From left to right: ^*^P = 0.0142, ^**^P = 0.0018, ns *P* = 0.73, ns *P* = 0.79, respectively, by two-sided *t* test. **d** Representative H&E staining (left panel) and quantification (right panel) analysis of lung metastasis in **c**. *n* = 6 mice per group. Scale bars, 2 mm. From left to right: ^***^P = 0.0001, ^***^P = 0.0003, ns *P* = 0.47, ns *P* = 0.29, respectively, by two-sided *t* test. **e** LM2 cells infected with the indicated lentiviruses were injected into the mammary fat pads of nude mice. Representative bioluminescent images (BLI) of mice with spontaneous lung metastasis following daily treatment with ASP/NAM for 30 days (left panel), and quantification of BLI (right panel) is shown. *n* = 6 mice per group. From left to right: ^**^P = 0.0012, ^***^P = 0.0005, ns *P* = 0.70, respectively, by two-sided *t* test. **f** Representative H&E staining (left panel) and quantification (right panel) analysis of lung metastasis in **e**. *n* = 6 mice per group. Scale bars, 2 mm. From left to right: ^**^P = 0.0034, ^***^P = 0.0002, ns *P* = 0.80, respectively, by two-sided *t* test. **g** IP and IB analysis of acetyl-K177 METTL3 levels in xenografted tumors. **h**, **i** Representative IHC staining (**h**) and quantification of cytoplasmic METTL3 (**i**) in xenografted tumors. Scale bars, 25 μm. *n* = 6 mice per group. ^***^P = 3.07e-06 by two-sided *t* test. **j** LC-MS/MS quantification of the m^6^A/A ratio in polyadenylated RNA isolated from xenografted tumors. *n* = 5 mice in WT, WT + A/N and KQ + A/N group, *n* = 4 mice in per other groups. From left to right: ^***^P = 0.0003, ^*^P = 0.020, ns *P* = 0.13, ^*^P = 0.031, ns *P* = 0.34, respectively, by two-sided *t* test. **k** IP and IB analysis of acetyl-K177 METTL3 levels in tissue lysates extracted from fresh human breast cancer samples. All data are represented as mean ± SEM. All *P* values were calculated by Student’s *t* test. Source data are provided as a Source Data file.

To investigate the contribution of METTL3 acetylation in controlling cancer metastasis in vivo, we generated LM2 METTL3 reconstituting cells (LM2, a derivative of MDA-MB-231 cells with strong lung metastatic potential) (Supplementary Fig. 7a, b). Upon fat pad-injection of LM2 cells, we found METTL3 silencing markedly suppressed lung metastases (Fig. 7a, b), which can be significantly restored by METTL3^WT^ or METTL3^K177R^ reconstitution, but not by METTL3^K177Q^ complementation. We further demonstrated that METTL3-mediated metastasis relied on intact IL-6R signaling (Supplementary Fig. 7c). Notably, METTL3 depletion or reconstitution showed no detectable effects on overall tumor volume (Supplementary Fig. 7d).

To explore the therapeutic potential of ASP/NAM in preventing lung metastases in vivo, mice xenografted with METTL3 reconstituting cells were treated with ASP (50 mg/kg) and NAM (50 mg/kg) by daily intraperitoneal injection for 30 days, followed by quantitative bioluminescence imaging analyses. ASP/NAM administration significantly blunted lung metastases in mice receiving METTL3^WT^ cells (Fig. 7c, d), but showed no effect on metastatic properties in mice bearing METTL3^K177Q^ or METTL3^K177R^ xenografts. Similar results were obtained in ASP/shSIRT1-treated groups (Supplementary Fig. 7e, f). Of interest, ASP/NAM displayed no major influence on tumor volume (Supplementary Fig. 7g), and ASP or NAM alone did not change lung metastasis rate (Supplementary Fig. 7h). Also, ASP/NAM treatment failed to further suppress metastasis in vivo in the absence of METTL3, supporting the notion that the anti-metastasis role of ASP/NAM is METTL3-dependent (Fig. 7e, f).

We next investigated if ASP/NAM co-treatment can induce METTL3 acetylation and subsequent m^6^A-associated alterations in tumor xenografts. K177-acetylation was observed in METTL3^WT^ xenografted tumor samples treated with ASP/NAM. Similar data were obtained in ASP/shSIRT1-treated tumors (Fig. 7g and Supplementary Fig. 7i), albeit to a lesser extent compared to acetyl-K177 signals in ASP/NAM- or ASP/shSIRT1-treated MDA-MB-231 cells, likely reflecting differential concentrations of ASP/NAM or ASP administrated in vitro and in vivo. Nevertheless, this enhanced METTL3 acetylation was accompanied by increased staining of cytosolic METTL3 (Fig. 7h, i and Supplementary Fig. 7j, k). Accordingly, ASP/NAM or ASP/shSIRT1 significantly reduced METTL3-mediated global m^6^A abundance only in mice receiving METTL3^WT^ LM2 cells (Fig. 7j and Supplementary Fig. 7l, m), abolished m^6^A deposition on our selected gene candidates (Supplementary Fig. 7n–p**)**, and altered their RNA/protein expression (Supplementary Fig. 7q–v). Meanwhile, protein levels of BRD4, PTPN14, and LPP exhibited consistently enhanced expression in METTL3^K177Q^ xenografted tumors, compared to tumors derived from METTL3^WT^-LM2 transplantation (Supplementary Fig. 7w).

To further validate the correlation between METTL3 acetylation and tumor metastasis, we assessed acetyl-METTL3 levels by immunoprecipitating METTL3 from protein lysates extracted from fresh breast carcinoma samples. Strong K177 acetylation signals were observed in three out of five in situ breast cancer tissues, whereas tumor tissues with node metastasis showed undetectable or largely reduced acetyl-METTL3 signals (Fig. 7k), suggestive of an inverse correlation between METTL3 acetylation and breast cancer metastasis in the clinical samples.

**Fig. 8:**
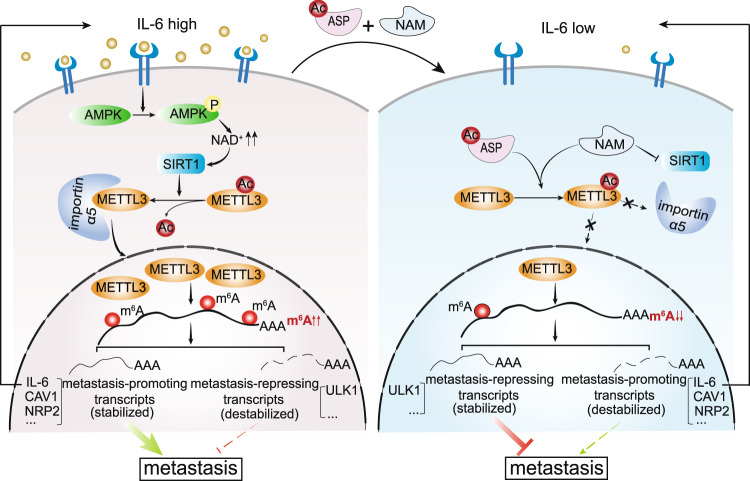
METTL3 acetylation impedes cancer metastasis via fine-tuning its nuclear and cytosolic functions. Proposed model for pathophysiological stimuli-mediated METTL3 acetylation/deacetylation and its functional implications.

Altogether, we propose that acetyl-K177-METTL3 level, and/or the percentage of nuclear METTL3-positive tumor cells, may serve as prognostic factors predicting metastasis potential in breast cancer patients. Furthermore, the tight connection between METTL3 acetylation and node metastasis indicates that targeting METTL3 deacetylation by ASP/NAM may provide therapeutic advantages in breast cancer patients with node metastasis (Fig. 8).

### Detection of cytosolic METTL3 by different antibodies

Given METTL3 has been considered as a bona fide nuclear protein, the widespread cytoplasmic localization of METTL3 is intriguing yet puzzling. In fact, the pathophysiological importance of cytosolic METTL3 functions has not been explored so far, likely owing to its cytoplasmic localization has been rarely reported. We carefully went through related publications and found only a few published literatures clearly showed cytoplasmic localization of METTL3^24,52–54^. A noticeable difference among these reports is the METTL3 antibody used to perform IF staining or immunoblotting. Anti-METTL3 antibody from Abcam (Cat# ab195352, hereinafter termed anti-M3-A) has been the most widely used one so far. In our study, an anti-METTL3 antibody from Proteintech was utilized (Cat# 15073-1-AP, hereinafter termed anti-M3-P). Previously reported discrepancies regarding cellular distribution (i.e., SIRT1^55^) or conformation-dependent antibody recognition (i.e., p53^56^) issues caught our attention, which prompted us to investigate the differences between anti-M3-A and anti-M3-P in detecting cytosolic METTL3.

We first verified the specificities of both A and P antibodies using METTL3-depleted cells by WB and IF staining (Supplementary Fig. 8a–f). Then, comparison of A and P antibodies was conducted utilizing the following experimental conditions to detect cytosolic METTL3. (i) Translocation of endogenous M3 upon ASP/NAM or rIL-6 treatment (Supplementary Fig. 8a–l). Anti-M3-P was capable of detecting cytoplasmic METTL3 by both IF and WB. Anti-M3-A only recognized cytosolic METTL3 by WB analysis, but not by IF staining. (ii) ASP/NAM-induced translocation of reconstituted Myc-METTL3^WT^. In this experiment, we compared the ability of anti-M3-A, anti-M3-P and anti-Myc antibodies in detecting the translocation of reconstituted METTL3^WT^ upon ASP/NAM treatment. As shown in Supplementary Fig. 8m, n, consistent IF staining results were obtained between anti-Myc and M3-P antibodies, showing nuclear to cytoplasmic translocation of METTL3. Anti-M3-A failed to detect cytosolic METTL3^WT^ signals by IF staining. All three antibodies detected cytoplasmic METTL3 by WB with similar efficiency. (iii) WTAP depletion-induced nuclear to cytosolic shuttling of endogenous METTL3 (Supplementary Fig. 8o, p). Again, unlike anti-M3-P, anti-M3-A is incapable of detecting cytoplasmic METTL3 by IF staining. We conclude that both M3-A and M3-P antibodies can recognize specific METTL3 signals, both in the nucleus and in the cytosol, by WB under denaturing conditions. However, when it comes to IF staining, which in general does not denature proteins, the M3-A appears to be much less effective or even defective in recognizing cytoplasmic METTL3, compared to the M3-P antibody. Both antibodies can recognize nuclear METTL3 with comparable efficiency.

Epitope recognizing process can be influenced by many factors, including whether proteins are linearized, sample preparation like formalin fixation, and the dynamic switching of binding partners. Growing body of evidence has demonstrated that METTL3 can form distinct protein complexes in different cellular compartments. For instance, METTL3 has been shown to associate with WTAP only in the nucleus under basal conditions. These distinct binding partners may cause conformational differences between nuclear and cytosolic METTL3, which ultimately influences its antibody recognition by assays like IF staining. A plausible explanation to the failed detection of cytosolic METTL3 by M3-A antibody might be attributed to the inaccessibility of epitope recognized by this particular antibody under native conditions. The anti-M3-P antibody was generated using METTL3 fusion protein (aa229-aa580) as immunogen. Unfortunately, the immunogen information for M3-A antibody from the Abcam website is not available. Nevertheless, we believe cytosolic METTL3, with varying abundance across mammalian cell lines, is physiologically relevant. We were curious if cytosolic localization of METTL3 could be detected in normal tissues and therefore examined METTL3 distribution in E15.5 mouse embryos. As shown in Supplementary Fig. 8q, METTL3 is found in both the nucleus and cytosol in most developing organs, suggestive of potential physiological roles of cytoplasmic METTL3 during development.

## Discussion

The multifaceted role of METTL3, in both m^6^A-dependent and -independent manners, is primarily determined by its dynamic distribution in different cellular compartments. However, the PTMs that can influence the localization of METTL3 are completely unknown. Here, we demonstrate that METTL3 acetylation represents a key post-translation modification determining METTL3 translocation between the nucleus and the cytosol. We further identified the physiological stimuli that can either drive or hamper METTL3 nuclear entry. IL-6-induced deacetylation facilitates nuclear shift of METTL3 via AMPK/SIRT1 axis, whereas ASP/NAM-mediated acetylation attenuates its cytoplasm to nucleus translocation.

Diverse signaling networks have been reported to modulate IL-6 expression via different mechanisms. It’s intriguing that cell-type-dependent variations in IL-6 abundance may dictate METTL3 subcellular localization. Given a prominent role of IL-6 in driving metastasis through autocrine and paracrine networks, aberrantly high IL-6 levels, either within the tumor microenvironment (TME) or within tumor cells, may therefore drive a distinct transcriptional signature enriched for genes involved in promoting metastasis via m^6^A-dependent mechanisms. Meanwhile, IL-6 is also a well-known senescence-associated secretory phenotype (SASP) gene, and a recent study has reported that genome-wide redistribution of METTL3 and METTL14 can drive the SASP via transcriptional regulation in an m^6^A-independent manner^41^. These data highlight a versatile role of METTL3, by operating distinct modes of action, in regulating stress responses.

Given the critical role of METTL3 in a broad range of cellular processes, acetylation-mediated cytosolic retention of METTL3 may have dual impacts on METTL3 functions. On one hand, acetylation disables METTL3 nuclear entry, therefore blunting its nuclear functions, leading to reduced m^6^A modification, impaired protein synthesis specifically engaging promoter-bound METTL3-mediated translation^26^, and repressed gene transcription via both m^6^A-dependent^7^ and -independent pathways^41^. On the other hand, increased cytosolic pool of METTL3 may exert a positive effect on translation via either m^6^A-dependent^12,57^ or m^6^A-independent mechanisms^27,28^. In our study, acetyl-mimetic K177Q mutant displayed enhanced protein translation via increased METTL3-eIF3h complex formation. Since METTL3-mediated translation relies on both nuclear and cytosolic pools of METTL3, this may explain why K177Q only exhibited partial effect on protein translation from our polysome-profiling data. Meanwhile, METTL3 has been shown to promote translation of a large subset of oncogenic mRNAs, thereby promoting growth, survival and invasion of cancer cells^27,28^. In our study, increased translation of both oncogenic and tumor suppressor mRNAs was found in K177Q reconstituting cells, demonstrating the nuclear and cytosolic functions of METTL3 may act in concert with cell/tissue type in determining cell fate. Of note, we did not further pursue the influence of ASP/NAM or IL-6 treatment on translation, since they both have active crosstalks with signaling networks regulating protein synthesis, for instances, mTOR pathway^40,58,59^. Nevertheless, the dynamic switch between acetylation and deacetylation triggered by distinct stress stimuli may evolve to enable METTL3 to sense environmental cues and oncogenic insults, thereby eliciting either rapid or prolonged actions, via coordinating mRNA modification and the transcriptional/translational machineries.

We propose that K177 acetylation may compromise the nuclear functions of METTL3. However, the substrate and peak differences between KQ-m^6^A-signature and M3-KD-m^6^A signature, which likely reflect context-specific deposition of m^6^A on RNA substrate, suggesting K177 acetylation may have alternative functions via either direct or indirect mechanisms. The following clues may provide mechanistic hints explaining how context-dependent signaling may influence m^6^A modification. First, threshold-dependency for METTL3 and METTL14 has been reported when catalyzing m^6^A methylation^60^. Second, functional compensation may exist between METTL3 and other yet-to-be-identified N6-methyladenosine RNA methyltransferases, when cellular METTL3 is acutely depleted. Thirdly, a subset of m^6^A sites was found to be dynamically regulated in a stress-dependent manner^6,61–63^. These lines of evidence may also explain why the number of differential m^6^A peaks caused by METTL3 depletion varied among different experimental settings.

Emerging evidence has suggested pharmacological inhibition of METTL3 might be a promising therapeutic approach for cancer treatment^64^. Unlike the enzymatic inhibitor, ASP/NAM may specifically target incipient metastatic cells endowed with traits needed to generate metastases, for instances, hypo-acetylated METTL3. Daily ASP intake has been associated with reduced risk of metastases, but the underlying mechanism is poorly understood. Nicotinamide, the amide form of vitamin B3, has a good safety profile and has been actively tested in clinical trials as a treatment for a wide range of diseases^65,66^. It will be interesting to investigate the efficacy of ASP/NAM, together with chemotherapy, immunotherapy or targeted therapy, in metastasis prevention and treatment.

## Methods

### Study approval

All mouse experiments were approved and followed guidelines established by the Institutional Animal Care and Use Committee of Xiamen University (XMULAC 20170331). These mouse experiments were in strict accordance with good animal practice as defined by the Xiamen University Laboratory Animal Center. All studies with clinical specimens were carried out according to the Declaration of Helsinki and performed in compliance with government policies, and the “Guidance of the Ministry of Science and Technology (MOST) for the Review and Approval of Human Genetic Resources.” The use of clinical specimens was approved by the Institutional Ethics Committee of Xijing Hospital of Fourth Military Medical University.

### Clinical specimens

Breast, prostate and colon cancer tissue microarrays and the corresponding normal tissue microarrays were obtained from patients who had undergone surgery at the Xijing Hospital. In all, 11 breast tumor tissue samples for protein extraction were freshly frozen in dry ice and stored at −80 °C until used. Patients provided written informed consent. The clinicopathological characteristics, including details of human research participants (such as age, gender, etc.) are summarized in Supplementary Table 6.

### Cell culture

HEK293T (CRL-3216), MDA-MB-231 (HTB-26), MCF-7 (HTB-22), Hs578T (HTB-126), MDA-MB-436 (HTB-130), MDA-MB-468 (HTB-132), HCC1937 (CRL-2336), BT-474 (HTB-20), BT-483 (HTB-121), SKBR3 (HTB-30), T47D (HTB-133), DU145 (HTB-81), LNCaP (CRL-1740), PC3 (CRL-1435), 22RV1 (CRL-2505), SW-620 (CCL-227), and RKO (CRL-2577) cell lines were originally obtained from ATCC; LM2 cell line was a gift from Dr. Guohong Hu (Institute of Health Sciences, Shanghai, China); SUM-159 cell line was a gift from Dr. Suling Liu (Cancer Institute, Fudan University, Shanghai, China); OVCAR4 and KURAMOCHI cell line were a gift from Jing Tan (Sun Yat-sen University Cancer Center, Guangzhou, China). HEK293T, MDA-MB-231, MCF-7, LM2, SW-620, RKO, Hs578T and MDA-MB-468 cell lines were maintained in DMEM (Gibco) supplemented with 10% FBS. MDA-MB-436 cells were maintained in DMEM/F12 supplemented with 10% FBS. HCC1937, OVCAR4, KURAMOCHI, DU145, LNCaP, PC3, and 22RV1 cells were cultured in RPMI1640 supplemented with 10% FBS. T47D and BT-483 cells were maintained in RPMI1640 supplemented with 10% FBS and insulin. SUM-159 cells were cultured in F12 supplemented with 10% FBS, insulin and hydrocortisone. SUM-1315 cells were cultured in F12 supplemented with 10% FBS, insulin and EGF. BT-474 cells were maintained in Hybri-Care medium supplemented with 10% FBS. SKBR3 cells were cultured in McCoy’s 5A medium supplemented with 10% FBS. All cells were maintained at 37 °C in a saturated humidity atmosphere containing 95% air and 5% CO_2_. For aspirin/NAM treatment, cells were treated for the indicated time. For rIL-6 treatment, cells were washed with phosphate-buffered saline (PBS), cultured in DMEM with 0.05% FBS for 24 h, and then treated with 100 ng/mL rIL-6 for the indicated time. For ActD treatment, cells were exposed to 5 or 10 μg/mL ActD for the indicated time.

### Plasmid constructs

cDNAs encoding GCN5, PCAF, CBP, p300, TIP60, MOZ, HBO1, SIRT1–7, METTL3, METTL14, and importins were amplified and cloned into pcDNA3.1. cDNA encoding WTAP was amplified and cloned into LVEX08-3×Flag vector. cDNA encoding eIF3h was amplified and cloned into pGEX-4T-1. cDNAs encoding CAV1, IL-6 and NRP2 were amplified and cloned into pLvx-Flag vector. pGEX-4T-1-METTL3 and pEGFP-C3-METTL3 were subcloned from pcDNA3.1-METTL3. pET-21b-METTL14 was subcloned from pcDNA3.1-METTL14.To generate lentiviral expression constructs, cDNA encoding METTL3 was amplified and cloned into pLV-EGFP vector. To generate baculovirus expression constructs, cDNA encoding METTL3 was amplified and cloned into pFastBac. All METTL3 mutant constructs were generated by a PCR-based site-directed mutagenesis method using wild-type METTL3 construct as a template.

### Reagents and antibodies

Acetyl-CoA (A2181), β-Nicotinamide adenine dunucleotide (NAD^+^) (N1511), NAM (N0636), MTT (M2128), TRIzol (T9424), alkaline phosphatase (P5931) and nuclease P1 (N8630) were purchased from Sigma. TSA (HY-15144), Myc peptide (HY-P0312), adenosine (HY-B0228) and guanosine (HY-N0097) were purchased from MCE. RevertAid Reverse Transcriptase (EP0442), Dynabeads purification kit (61006), RNA fragmentation reagents (AM8740), and puromycin (A1113803) were purchased from Thermo Fisher Scientific. Luciferin (E160E), RNasin (N2515), and NAD/NADPH-Glo Assay (G9072) were purchased from Promega. Myc-agarose (GNI4510-MC), Flag-agarose (GNI4510-FG) and HA-agarose (GNI4510-HA) were purchased from GNI. Cycloheximide (94271) was purchased from Amresco. Protease inhibitors (04693132001) and RNase A (RNASEA-RO) were purchased from Roche. DNase I (M0303) was purchased from NEB. N6-methyladenosine (PR3732) was purchased from Berry & Associates. Aspirin (50-78-2) was purchased from MERYER. aspirin-*d*_*3*_ (A874689) was purchased from MACKLIN. rIL-6 (200-06) was purchased from PEPROTECH. Chamber matrigel invasion well (354480) was purchased from BD. Human IL-6 Quantikine ELISA kit (D6050) was purchased from R&D. Transwell (3422) and Matrigel Matrix (356231) were purchased from Corning. ChamQ Universal SYBR qPCR Master Mix was purchased from Vazyme. Phos-tag™ Acrylamide (AAL-107) was purchased from Wako. UltraSensitive SP rabbit (KIT-9707), MaxVison DAB (KIT-0014) and antigen retrieval solution (MVS-0100) were purchased from Maxim. Biotinylated METTL3 peptides containing either acetylated or non-acetylated K177 residue were synthesized by Sangon Biotech. *d*_*3*_-SAM and *d*_*3*_-N^6^-methyladenosine were synthesized by EFEBIO.

The antibody that specifically recognizes acetylated METTL3 was raised by immunizing rabbits with synthetic peptides “EVAGTVTGQ(Ac-K)RRAEQDS” for Ac-K177-METTL3 (dilution 1:1000). Anti-acetylated Lys (9441, 1:1000), anti-p300 (86377, 1:1000), anti-BRD4 (13440, 1:1000), anti-Myc-tag (mouse, 2276, Clone No. 9B11, 1:10000 for WB; 1:800 for IF), anti-AMPKα (2532, 1:1000) and anti-phospho-AMPKα-T172 (2535, 1:1000) were obtained from Cell Signaling Technology. Anti-METTL3 (15073-1-AP, 1:1000 for WB; 1:100 for IF; 1:500 for IHC) for western blotting and IF, anti-METTL14 (26158-1-AP, 1:1000 for WB; 1:100 for IF; 1:500 for IHC), anti-WTAP (10200-1-AP, 1:1000 for WB; 1:100 for IF; 1:500 for IHC), anti-SIRT1 (13161-1-AP, 1:3000), anti-SIRT2 (19655-1-AP, 1:3000), anti-SIRT3 (10099-1-AP, 1:3000), anti-importin α5 (18137-1-AP, 1:5000), anti-eIF3h (11310-1-AP, 1:1000), anti-eIF3b (10319-1-AP, 1:1000), anti-CBP80 (10349-1-AP, 1:1000), anti-YTHDF2 (24744-1-AP, 1:1000), anti-GFP (66002-1-lg, Clone No. 1E10H7, 1:10000 for WB), anti-Myc (16286-1-AP, 1:10000), anti-Flag (20543-1-AP, 1:30000), anti-GST (10000-0-AP, 1:30000), anti-ACTIN (66009-1-lg, Clone No. 2D4H5, 1:5000), anti-Tubulin (66240-1-AP, Clone No. 1D4A4, 1:5000) and anti-Lamin B1 (66095-1-lg, Clone No. 3C10G12, 1:5000) were obtained from Proteintech Group. Anti-METTL3 (MA5-27527, 1 μg/mg proteins for IP) for immunoprecipitation was obtained from Thermo Fisher Scientific. Anti-HA (12158167001, Clone No. 3F10, 1:1000) was obtained from Roche. Anti-CBP (A1334, 1:1000), anti-IL6R (A1570, 1:500), anti-IGF2BP1 (A1517, 1:1000), anti-IGF2BP3 (A6099, 1:1000), anti-KIAA1429 (A16201, 1:1000 for WB; 1:100 for IF; 1:500 for IHC), anti-RBM15 (A4936, 1:1000 for WB; 1:100 for IF; 1:500 for IHC), anti-PTPN14 (A12093, 1:500), anti-LPP (A19226, 1:30000), anti-CAV1 (A19006, 1:30000) and anti-NRP2 (A2581, 1:500) were obtained from Abclonal. Anti-GAPDH (sc32233, Clone No. 6C5, 1:10000) was obtained from Santa Cruz Biotechnology. Anti-puromycin (MABE343, Clone No. 12D10, 1:10000) was obtained from Millipore. Anti-m^6^A (202003, 2 μg/μg RNA for MeRIP) was obtained from Synaptic Systems. Anti-ULK1 (A7481, 1:1000) was obtained from Sigma. Anti-pan methylated lysine (Abm40195, 1:1000) was obtained from Abbkine. Anti-METTL3 (ab195352, 1:2000 for WB; 1:100 for IF; 1:500 for IHC) was obtained from Abcam. Anti-Zc3h13 (A300-748A, 1:1000 for WB; 1:100 for IF; 1:100 for IHC) was obtained from Bethyl.

### RNA interference

The lentivirus-based vector pLV-H1-EF1α (Biosettia) was used for the expression of shRNAs. shRNA sequences for target genes as follows.

shMETTL3#1, 5’-GGGCCCAAGTGCAAGAATTCT-3’;

shMETTL3#2, 5’-GCTGCACTTCAGACGAATT-3’;

shp300#1, 5’-ATACTCAGCCGGAGGATATTT-3’;

shp300#2, 5’-CTTCACAATTCCGAGACA-3’;

shCBP#1, 5’-GCATGAATGCTAACTTTAACC-3’;

shCBP#2, 5’-GCCAGTGAATCGCATGCAAGT-3’;

shSIRT1, 5’-GACACTGTGGCAGATTGTTATTAAT-3’;

shSIRT2, 5’-GCATGGACTTTGACTCCAAGA-3’;

shSIRT3, 5’-GGAAACTGGGAAGCTTGATGG-3’;

sheIF3h, 5’-GATAGATGGCCTTGTGGTA-3’;

shAMPKα, 5’-GCGTGTACGAAGGAAGAATCC-3’.

shULK1, 5’-GGTACCTCCAGAGCAACATGA-3’.

shYTHDF2, 5’-GGTAGCACAGAAGTTGCAAGC-3’.

shIGF2BP1, 5’-GCCTTAAAGGATGGTTCATTT-3’.

shIGF2BP3, 5’-GCAAAGGATTCGGAAACTTCA-3’.

shIL6R, 5’-GCTCTTGGTGAGGAAGTTTCA-3’.

shPTPN14, 5’-GGATGGATTTGGACAGGAAAT-3’.

shLPP, 5’-GCCTATCATCCTCACTGTTTC-3’.

shNRP2, 5’-GCAAGTTCAAAGTCTCCTACA-3’.

Lentiviruses were generated according to the manufacturer’s protocol. The viruses were used to infect cells in the presence of protamine sulfate (8 μg/mL).

### Immunoblotting and immunoprecipitation

Cells were lysed with RIPA buffer (50 mM Tris pH 8.0, 150 mM NaCl, 1% Triton, 0.1% SDS, 1 mM EDTA, 1% sodium deoxycholic acid, 1 mM Na_3_VO_4_, 10 mM NaF) with protease inhibitor and PMSF (phenylmethylsulfonyl fluoride). Lysates were separated by SDS-PAGE and transferred to polyvinylidene fluoride membranes. The membranes were incubated with the indicated antibodies. For immunoprecipitation, cell lysis was carried out with lysis buffer (50 mM Tris pH 7.4, 150 mM NaCl, 1% Triton, 40 mM β-glycerophosphate, 2 mM EDTA, 1 mM Na_3_VO_4_, 10 mM NaF) supplemented with protease inhibitor and 5 mM NAM. Lysates were then immunoprecipitated with Myc-agarose, Flag-agarose or HA-agarose, or anti-METTL3 antibody (Thermo Fisher Scientific). Immune complexes were then washed five times with NETN buffer (50 mM Tris-HCl pH 8.0, 120 mM NaCl, 1 mM EDTA, 0.5% NP-40). Proteins were eluted in an SDS sample buffer and subjected to immunoblotting analysis. Uncropped scans of western blots presented in the main figures and supplementary figures are provided in Source Data.

### Quantitative RT-PCR

Total RNA was isolated from samples with TRIzol reagents following the manufacturer’s instructions. cDNA was prepared with the RevertAid Reverse Transcriptase. Quantitative PCR was performed using the StepOne-Plus real-time PCR system (Applied Biosystems Inc., Foster City, CA). Primers used for QRT-PCR in this study are listed in Supplementary Table 4.

### Cell labeling by SILAC and LC-MS/MS analysis

To identify acetylation site(s) of METTL3, HEK293T cells were grown in SILAC DMEM (Thermo Fisher Scientific) supplemented with L-lysine/arginine (Sigma) (light label) or l-lysine/arginine-U-^13^C6 (Cambridge Isotope Laboratories) (heavy label) together with 10% dialyzed FBS, l-glutamine, and penicillin/streptomycin for 2 weeks (light labeled: arginine 84 mg/L and lysine 146 mg/L; heavy labeled: arginine 87.2 mg/L and lysine 152.8 mg/L). Cells were cultured at 37 °C in a humidified atmosphere containing 5% CO_2_. Light and heavily labeled cells were transfected with Myc-METTL3 alone or Myc-METTL3 plus HA-p300, respectively. Cells were then harvested, centrifuged (5 min, 500×*g*), rinsed twice with ice-cold phosphate-buffered saline (PBS) and stored at −80 °C briefly. Cells were lysed, followed by immunoprecipitation with Myc-agarose, washed and then eluted with Myc peptides. Elutes were subjected to solution digestion and LC-MS/MS analysis following the protocol described below.

For recognition of METTL3 acetylation site(s) with HA-p300 transfection or aspirin/NAM treatment, mass spectrometry was performed on a nanoscale EASY-nLC 1200UHPLC system (Thermo Fisher Scientific) connected to an Orbitrap Fusion Lumos equipped with a nanoelectrospray source (Thermo Fisher Scientific). Mobile phase A contained 0.1% formic acid (v/v) in water; mobile phase B contained 0.1% formic acid in 80% acetonitrile (ACN). The peptides were dissolved in 0.1% formic acid (FA) with 2% acetonitrile and separated on an RP-HPLC analytical column (75 μm × 25 cm) packed with 2 μm C18 beads (Thermo Fisher Scientific) using a linear gradient ranging from 8% to 30% ACN in 90 min, followed by a linear increase to 44% B in 20 min at a flow rate of 300 nL/min. The Orbitrap Fusion Lumos acquired data in a data-dependent manner alternating between full-scan MS and MS2 scans. The spray voltage was set at 2.2 kV and the temperature of ion transfer capillary was 300 °C. The MS spectra (350–1500 *m/z*) were collected with 120,000 resolutions, AGC of 4 × 10^5^, and 50 ms maximal injection time. Selected ions were sequentially fragmented in a 3 second (s) cycle by HCD with 30% normalized collision energy, specified isolated windows 1.6 *m/z*, and 30,000 resolutions. AGC of 5 × 10^4^ and 150 ms maximal injection time were used. Dynamic exclusion was set to 15 s. Unassigned ions or those with a charge of 1+ and >7+ were rejected for MS/MS. MS and MS/MS data were acquired using the Xcalibur software (Thermo Fisher Scientific, v4.0) and analyzed using the Proteome Discoverer (PD, version 2.2).

### Migration/invasion assays

For in vitro migration assay, an 8 μm pore size Boyden chamber was used. Cells (200 μL, 1 × 10^5^) in 0.2% serum-containing medium were plated in the upper chamber, and 600 μL 10% FBS was added to medium in the lower chamber as a chemoattractant. For invasion assay, an 8-μm pore size BD Matrigel Invasion Chamber was used. Unless otherwise indicated, migration/invasion was performed as follows: after 4 h (MDA-MB-231) or 72 h (MCF-7) for migration assay and 8 h (MDA-MB-231) or 144 h (MCF-7) for invasion assay, cells on the upper side of the filter were removed and cells that remained adherent to the underside of membranes were fixed in formaldehyde, followed by staining with crystal violet. The number of migrated cells was counted using a microscope. Four contiguous fields of each sample were examined using a 20× objective to obtain a representative number of cells that migrated/invaded across the membrane.

### In vitro acetylation and deacetylation assays

GST fusion constructs were expressed in BL21 *Escherichia coli* cells, and crude bacterial lysates were prepared by sonication in cold NETN buffer in the presence of the protease inhibitor mixture. Glutathione-Sepharose beads were used for the purification of GST-METTL3. For the acetylation reaction, immunopurified HA-tagged HATs (expressed in HEK293T cells) were incubated with GST-METTL3 in reaction buffer containing 20 mM Tris-HCl (pH 8.0), 20% glycerol, 100 mM KCl, 1 mM dithiothreitol (DTT) and 0.2 mM EDTA in the presence of absence of 100 μM acetyl-CoA. After incubation for 1 h at 30 °C, the reaction was stopped by SDS sample buffer. Samples were then subjected to SDS-PAGE and western blotting. For the deacetylation reaction, HEK293T cells transfected with Flag-tagged SIRTs were lysed, and then subjected to immunoprecipitation with Flag antibody-conjugated beads. The beads were then washed three times with lysis buffer and twice with 25 mM Tris-HCl (pH 7.5) buffer. The Flag-tagged SIRTs were then eluted with 3 × Flag peptide in 25 mM Tris-HCl (pH 7.5) buffer containing 150 mM NaCl and protease inhibitors. The acetylated METTL3 (prepared by immunoprecipitating the Myc-tagged METTL3 co-expressed with HA-p300 in HEK293T cells) was then incubated with immunopurified Flag-tagged SIRTs in reaction buffer containing 50 mM Tris-HCl (pH 9.0), 2 mM MgCl_2_, 50 mM NaCl, 0.5 mM DTT and 0.2 mM PMSF with or without 1.5 mM NAD^+^. After incubation for 1 h at 30 °C, the reaction was stopped by the addition of SDS sample buffer. Samples were then subjected to SDS-PAGE and western blotting.

### Cell viability assays

The MTT assay was used to measure cell viability. In total, 5000 Cells were seeded in 96-well plates, treated with aspirin, and assayed using MTT solution for 4 h. After the formazan crystals were dissolved with dimethyl sulfoxide, the absorbance was measured at 490 nm of wavelength to determine the cell viability with a microplate reader.

### Subcellular fractionation

Cells were lysed in hypotonic buffer (10 mM HEPES, pH7.5, 10 mM KCl, 1.5 mM MgCl, 1 mM DTT, 1× Protease Inhibitor Cocktail (Roche) and 1 mM PMSF) on ice for 15 min. NP-40 was then added to a final concentration of 0.25% for another 5 min. Samples were centrifuged for 5 min at 425×*g* at 4 °C, and the supernatant was saved as cytoplasmic fraction. The nuclear pellet obtained from the low speed centrifugation was washed with hypotonic buffer three times and re-suspended in hypertonic buffer (20 mM HEPES pH 7.6, 420 mM NaCl, 1.5 mM MgCl_2_, 0.5 mM DTT, 25% glycerol, protease inhibitor and 0.5 mM PMSF), followed by incubation on ice for 30 min. Samples were centrifuged for 5 min at 20,000×*g*, supernatants were saved as nuclear fractions. Samples were then subjected to SDS-PAGE and western blotting.

### Immunofluorescence staining

Cells grown on glass coverslips were washed three times with PBS, fixed with 4% paraformaldehyde for 10 min, and permeabilized with PBS containing 0.5% Triton X-100 for 10 min. Cells were then blocked with 1% BSA for 1 h, followed by incubating with the indicated antibody overnight at 4 °C. Cells were incubated with DAPI and goat anti-rabbit or mouse secondary antibodies (Alexa Flour 568 or 488, dilution 1:200) for 1 h at room temperature. Images were obtained using a Zeiss LSM 780 confocal microscope with ZEN 2010 software (Carl Zeiss GmbH, Jena, Germany). At least 50 cells were randomly selected and quantified for each group. The signal-positive area for METTL3 intensity levels was quantified using Image J (version 1.52p), and normalized over the signal-positive area for DAPI intensity levels, setting the same intensity threshold among different cell lines.

### GST pull-down assays

Around 10 μg of the appropriate GST fusion proteins were mixed with cell lysates. The binding reaction was mixed at 4 °C for 2 h. The beads were washed 5 times with NETN buffer (50 mM Tris-HCl pH 8.0, 120 mM NaCl, 1 mM EDTA, 0.5% NP-40). SDS loading buffer was added in the samples and boiled for 10 min. Samples were then subjected to SDS-PAGE and western blotting.

### RNA m^6^A quantification by LC-MS/MS

RNA m^6^A quantification by LC-MS/MS was performed as described previously^22^. In brief, total RNAs were isolated using TRIzol reagent according to the manufacturer’s instructions. Polyadenylated RNAs were extracted using Dynabeads mRNA purification kit (Thermo Fisher Scientific). mRNA concentration was measured by Qubit. 200 ng mRNA was digested by nuclease P1 (1 U) in 20 μL buffer containing 25 mM NaCl, 2.5 mM ZnCl_2_ at 37 °C for 2 h, followed by the addition of NH_4_HCO_3_ (1 M, 2.2 μl) and alkaline phosphatase (1 U, Sigma). After an additional incubation at 37 °C for 2 h, the solution was centrifuged at 20,000×*g* for 10 min at 4 °C, and 10 μL of the solution was injected into LC-MS/MS. Quantification was performed by comparison with the standard curve obtained from pure nucleoside standards. The ratio of m^6^A to A was calculated based on the calculated concentrations.

### In vitro m^6^A methyltransferase activity

In vitro methyltransferase activity assay was performed as described previously^22^. The reaction mixture which containing the following components: 0.15 nmol annealed RNA probe, 0.15 nmol recombinant METTL3, 0.8 mM *d*_*3*_-SAM, 80 mM KCl, 1.5 mM MgCl_2_, 0.2 U/μL RNasin, 10 mM DTT, 4% glycerol and 15 mM HEPES (pH 7.9) was incubated at 16 °C for 12 h. The resultant RNA was obtained by phenol/chloroform (pH 4.3–4.7) extraction and ethanol precipitation. After digestion by nuclease P1 and alkaline phosphatase, the amount of *d*_*3*_-m^6^A was measured by LC-MS/MS analysis. The nucleosides were quantified by using the nucleoside-to-base ion mass transitions of 285 to 153 (*d*_*3*_-m^6^A) and 284 to 152 (G). G served as an internal control to calculate the amount of RNA probe in each reaction mixture. RNA probe: 5’-ACGAGUCCUGGACUGAAACGGACUUGU-3’. The probe contains the consensus sequence of GGACU in the stem and loop with a stem-loop secondary structure in the presence of isotope-labeled cofactor *d*_*3*_-SAM. Recombinant METTL3 was expressed in insect cells with His-tag utilizing Bac-to-Bac baculovirus expression system based on previously published protocol^67^.

### MeRIP-seq and MeRIP-qPCR

MeRIP-seq was performed as previously described^6^. Briefly, total RNAs were extracted with TRIzol. Poly(A)^+^ RNAs were purified using Dynabeads mRNA purification kit, followed by fragmentation and immunoprecipitation with anti-m^6^A antibody (Synaptic Systems) in IP buffer (10 mM Tris-HCl, pH 7.4, 150 mM NaCl, 0.1% NP-40) for 2 h at 4 °C. The m^6^A-IP mixture was then incubated with Dynabeads protein A (Thermo Fisher Scientific) for an additional 2 h at 4 °C. The bound RNA was eluted by competition with N^6^-methyladenosine (Berry & Associates) and purified using an RNA cleanup kit (Zymo Research). The purified RNA fragments from m^6^A MeRIP and input were used for library construction and sequenced with Illumina NovaSeq6000 (Illumina) (conducted by Epibiotek Co.,Ltd, Guangzhou, China).

For MeRIP-qPCR, m^6^A-IP was performed as above. Fragmented purified mRNA was used in MeRIP-qPCR. Primer sequences are listed in Supplementary Table 5.

### MeRIP-seq data processing

MeRIP and input sequencing data were sent to Cutadapt (v2.5) to remove low-quality reads and adapter sequence contaminants under default parameters. The remaining reads were then aligned to human Ensemble genome GRCh38 using Hisat2 aligner (v2.1.0)^68^ under parameters: “--rna-strandness RF”. m^6^A peaks were identified using exomePeak R package (v2.13.2)　with PEAK_CUTOFF_PVALUE = 0.05, PEAK_CUTOFF_FDR = NA, FRAGMENT_LENGTH = 200 parameters. Differential m^6^A peaks were identified using exomePeak R package (v2.13.2) with FDR < 0.05. For differentially expressed genes analysis, the input samples from the m^6^A RNA immunoprecipitation were used for transcriptional profiling analysis. The expression of gene transcripts was quantified using featurecount (v1.6.3), and differential gene expression analysis was conducted using DESeq2 R package (1.18.1)^69^. Differentially expressed genes were identified as those with FC ≥ 2 and with a FDR ≤ 0.05. For MeRIP-seq data overlap analysis, overlap between METTL3^WT^-dependent m^6^A peaks and METTL3^K177Q^-dependent m^6^A peaks was identified by intersectBed v2.30.0 in bedtools toolset. For motif search, Homer (v4.10.4) was used to search for the enriched motif in the m^6^A peak region where *P* value <0.05 peaks were used for motif discovery. For m^6^A peak distribution, m^6^A peak distribution on the metagene was plotted by the R package Guitar (v1.16.0)^70^. Gene ontology (GO) analysis was performed using clusterProfiler and illustrated by ggplot2 package v3.3.0 in R. The gene ontology biological process terms with p value ≤ 1e^−2^ were included^71^. GSEA was conducted by java GSEA Desktop Application v4.0.3^72^ with default settings and considered gene set containing reported pro-metastatic genes associated with breast cancer selected from HCMDB. The Gene list is shown in Supplementary Table 1.

### Enzyme-linked immunosorbent assays (ELISA)

Supernatants were harvested and assayed for detection of IL-6 by ELISA kit according to the manufacturer’s protocol.

### Polysome fractionation and RNA-seq

Cells (six 150-mm dishes) were treated with 100 µg/mL cycloheximide for 5 min at 37 °C. Cells were then lysed with RNasin and layered onto 10–50% sucrose gradient tube and centrifuged at 154,000×*g* using SW41Ti rotor in Beckman Coulter (Optima L-80 ultracentrifuge) for 2 h at 4 °C. Gradients were fractionated and monitored at absorbance 260 nm (Biocomp). Collected fractions were pulled into sub-polysome fraction and polysome fraction, followed by RNA-seq. Poly(A)-selected mRNAs were purified and used for library construction using NEBNext® Ultra™ Directional RNA Library ep Kit for Illumina®, and sequenced with Illumina NovaSeq6000.

### SUnSET assays

For SUnSET assays^45^, cells were seeded in dishes 36 h prior to serum starvation. For serum stimulation, cells were maintained in regular media containing 10% FBS for 2 h. Puromycin pulses were performed by incubating the cells with 10 mg/mL puromycin for 15 min at 37 °C. Cells were then washed with cold PBS and lysed in RIPA buffer supplemented with PMSF and protease inhibitor. 5–10 μg of the whole cell lysates were assayed by western blotting using the anti-puromycin antibody.

### METTL3 RIP qPCR

The METTL3 RIP protocol was provided by Dr. Shuibin Lin (The First Affiliated Hospital of Sun Yat-sen University, China). In brief, MDA-MB-231 cells were crosslinked by UV, and then lysed in NETN lysis buffer (100 mM NaCl, 20 mM Tris-HCl (pH 8.0), 0.5 mM EDTA, 0.5% (v/v) NP-40). Lysates were incubated with anti-METTL3 (Proteintech, 4 μg) overnight at 4 °C. After washing, lysates containing antibody-protein-RNA complex were incubated with protein A/G sepharose at 4 °C for 1 h. The beads were washed, followed by suspending with NETN buffer containing proteinase K. The beads were then incubated at 45 °C for 45 min to reverse crosslinking. RNAs were obtained by TRIzol/chloroform extraction and ethanol precipitation. Precipitated RNAs were reverse transcribed using the RevertAid Reverse Transcriptase following the manufacturer’s instruction.

### Animal studies

All mice were maintained under specific pathogen-free conditions with a 12 h light/12 h dark cycle, at 22 ± 2 °C with humidity of 50 ± 5%, and were fed with a standard mouse chow diet at the Xiamen University Laboratory Animal Center. Six-week-old female Balb/c nude mice were used for all studies. For metastasis formation, cells were harvested, washed twice in PBS, counted, and then re-suspended in a 1:1 solution of PBS and Matrigel Matrix. Mice were anesthetized, a small incision was made to reveal the No.4 mammary fat pad, and luciferase-labeled cells (10^6^ for LM2 cells) were injected directly into the mammary fat pad. For aspirin and NAM injection, stock solution of aspirin, NAM or aspirin/NAM mixture was further diluted (final concentration for aspirin, 50 mg/kg; NAM, 50 mg/kg) in PBS containing 30% (v/v) PEG300 (S6704, Selleckchem) before intraperitoneal (i.p.) injection. After daily administration for 30 days, tumor volume was assessed by caliper measurements using the following formula: π (width^2^ × length)/6 (mm^3^). Tumor volume of mice did not exceed 1500 mm^3^ permitted by the Institutional Animal Care and Use Committee. 7 days after primary tumors were removed, mice were monitored by bioluminescent imaging for the development of metastases.

### Bioluminescent imaging and analysis

As previously described^73^, mice were anesthetized and injected with 1.5 mg of d-luciferin (15 mg/mL in PBS). Imaging was completed between 2 and 5 min after injection using a Xenogen IVIS Lumina system coupled to Living Image acquisition and analysis software (Xenogen). Images were analyzed with Living Image software ver. 3.0. Bioluminescent flux (photons/s/cm^2^/steradian) was determined for the mouse in a prone position.

### Histological assays

Haematoxylin and eosin staining was performed as previously described^43^. Briefly, the lungs were fixed in 4% (v/v) paraformaldehyde for 24 h, embedded in paraffin, and sectioned at 5 μm. The slides were dried at 45 °C for 12 h, deparaffinized with 100% xylene and a graduated series of ethanol from 100 to 50% ethanol, and then stained with hematoxylin and eosin (H&E) followed by washing with water. The slides were subjected to imaging using a Leica Aperio Versa 200. The human breast cancer tumor array was deparaffinized in 100% xylene and a graduated series of ethanol from 100 to 50% ethanol. After antigen retrieval was performed, all sections were blocked at room temperature in avidin/biotin blocking buffer (Maxim) and then 3% BSA for 30 min. Staining with antibodies was conducted overnight at 4 °C. Sections were rinsed twice in PBS, and protein staining was performed using a diaminobenzidine substrate kit (Maxim). Samples were counterstained with hematoxylin. The overall proportion of tumor cells with positive nuclear staining of METTL3 was evaluated as follows: an immunoreactive score (IRS) system was utilized for semi-quantitation of immunohistochemistry staining. IRS gives a range of 0–12 as a product of multiplication between proportion score (0–4) and staining intensity score (0–3). A proportion score represents the estimated percentage of positive-staining cells (0: no positive cells; 1: <10% of positive cells; 2: 10%–50% positive cells; 3: 51–80% positive cells; 4: >80% positive cells). An intensity score represents the average intensity of staining (0: no staining; 1: yellow, 2: claybank; and 3: tawny). Each sample was evaluated by three persons in a blinded manner and the mean score was considered as the final IRS.

For Fig. 1a, we measured the immunostaining score of METTL3 in 10 randomly selected fields at ×40 magnifications of tumor tissues. Staining of nuclear METTL3 was scored as 1 to 4 according to the percentage of nuclear-positive cells, as follows: 1, if <10% of tumor cells had nuclear staining; 2, if 11%–50%; 3, if 51%–70%; 4 if >70%. Samples showed moderate-to-strong nuclear staining intensity of METTL3 were scored. All slides were independently analyzed by two blinded observers. The images were obtained using an upright microscope Leica DM4B.

### Measurement of NAD^+^

We performed HPLC-MS for measurement of NAD^+^ as previously described^74^. Cell pellets were re-suspended in methanol-acetonitrile solution and followed by sonication. This solution was quick-freeze in liquid nitrogen and then thawed at room temperature, followed by sonication. After three times of freeze-thaw, the supernatant was taken and evaporated to dryness using a vacuum concentrator. The dry powder was reconstituted with acetonitrile solution and then sonicated. The supernatant was injected into HPLC-MS. The following transition was used for monitoring NAD^+^: 662.0/540.1. The retention time of NAD^+^ was 10.3 min.

### Statistics and reproducibility

Data are presented as mean ± SEM or mean ± SD, as indicated, of at least three independent experiments or biological replicates. Statistical significance was determined by two-tailed Student’s *t* test using Prism 8.0.2 software (GraphPad Software) unless otherwise indicated. *P* < 0.05 was considered significant. All data shown are representative of three independent experiments with similar results unless otherwise indicated.

### Reporting summary

Further information on research design is available in the Nature Research Reporting Summary linked to this article.

## Supplementary information

Supplementary Information

Reporting Summary

## Data Availability

The MeRIP-seq and RNA seq data generated in this study have been deposited in the Gene Expression Omnibus database under accession code GSE183017. The mass spectrometry proteomics raw data and processed data generated in this study have been deposited in the ProteomeXchange Consortium via the iProX partner repository with the dataset identifier PXD028177. The processed MeRIP-seq and RNA seq data are available in the Source Data. All data are available within the Article, Supplementary Information or Source Data file. Source data are provided with this paper.

## Source data

Source Data
