# Supplementary material for: METTL3 acetylation impedes cancer metastasis via fine-tuning its nuclear and cytosolic functions
Source: Nat Commun. 2022 Oct 26;13:6350. doi: 10.1038/s41467-022-34209-5 (PMC9605963; doi:10.1038/s41467-022-34209-5)
Supplement: Supplementary file 1 — Supplementary Information [file 41467_2022_34209_MOESM1_ESM.pdf]

Supplementary Information File

METTL3 acetylation impedes cancer metastasis via fine-tuning its nuclear and cytosolic functions

Yuanpei Li[1,*], Xiaoniu He[1,*], Xiao Lu[1], Zhicheng Gong[2], Qing Li[1], Lei Zhang[1], Ronghui Yang[3], Chengyi Wu[1], Jialiang Huang[1], Jiancheng Ding[4], Yaohui He[4], Wen Liu[4], Ceshi Chen[5], Bin Cao[6], Dawang Zhou[1], Yufeng Shi[7], Juxiang Chen[8], Chuangui Wang[9], Shengping Zhang[10], Jian Zhang[11], Jing Ye[12], Han You[1#]

[*]These authors contributed equally: Yuanpei Li, Xiaoniu He.

[#]Corresponding Author. e-mail: hyou@xmu.edu.cn

This PDF file includes:
Supplementary Figures 1 to 8
Supplementary Tables 1 to 6

Supplementary Figure 1

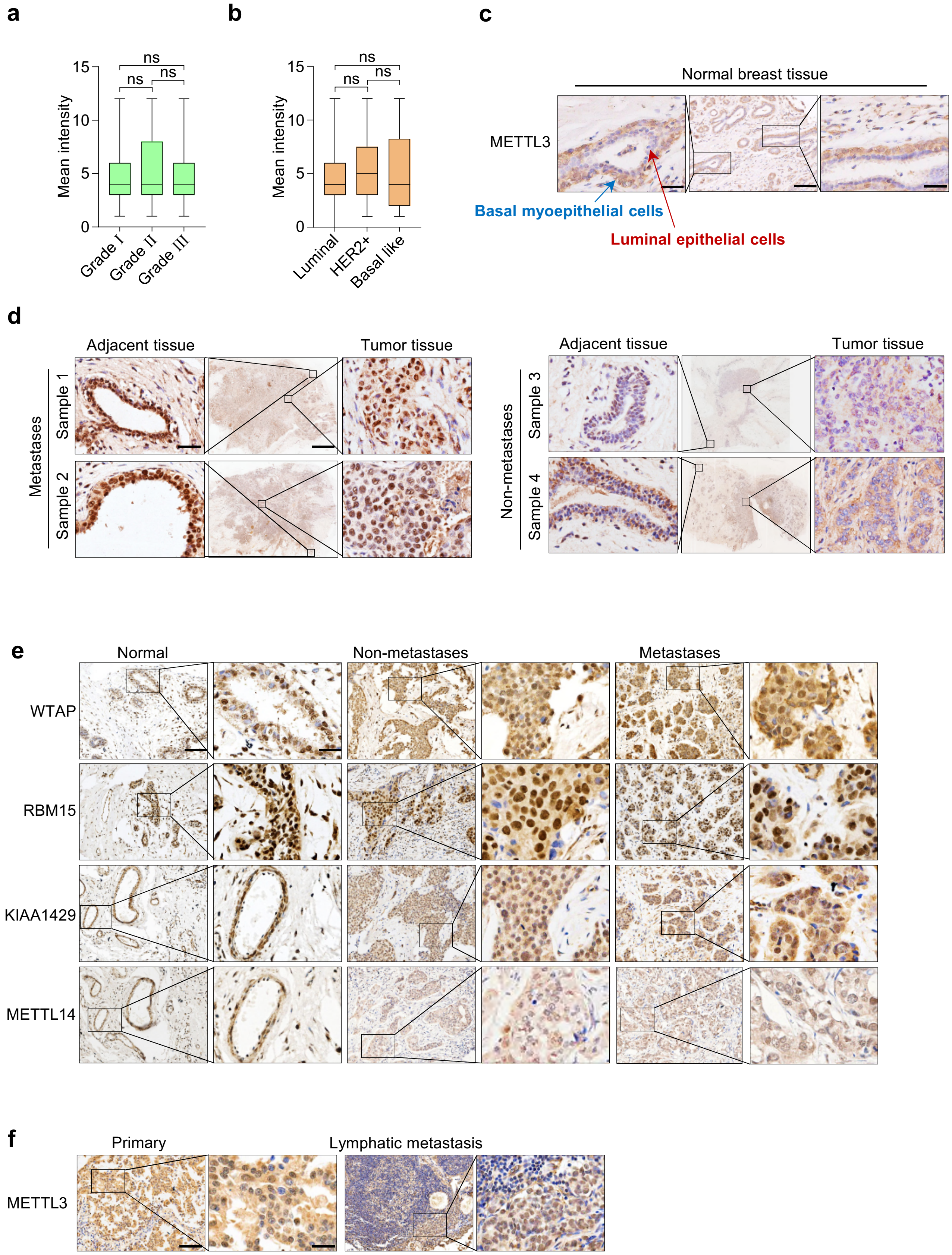

	Primary	Lymphatic metastasis
Nuclear METTL3 >70%	(84%) 27/32	(84%) 27/32

g

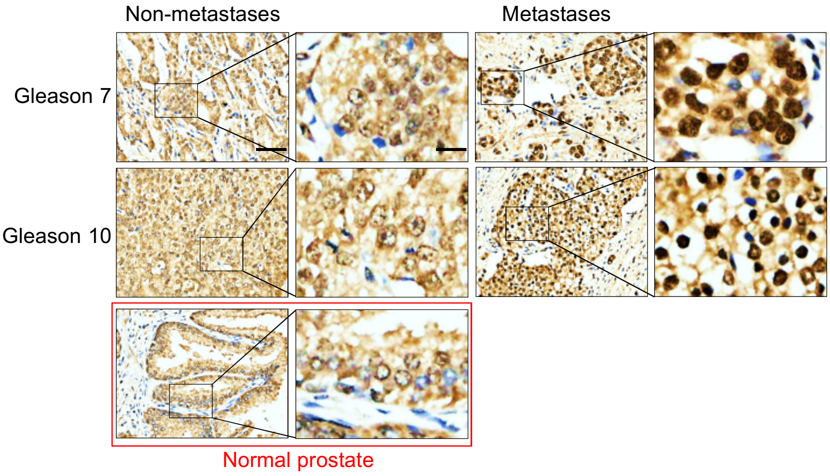

h

	Nuclear METTL3 <10%		Nuclear METTL3 >70%		*P*
Gleason score	G 6-7	G 8-10	G 6-7	G 8-10	
Non-metastases	(73%) 29/40	(65%) 30/46	(15%) 6/40	(11%) 5/46	4.40e-16
Metastases	(25%) 2/8	(13%) 1/8	(63%) 5/8	(75%) 6/8	0.0033

i

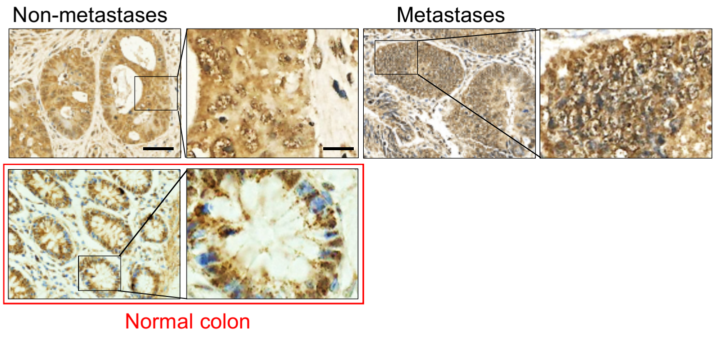

j

	Nuclear METTL3 <10%	Nuclear METTL3 >70%	*P*
Non-metastases	(68%) 41/60	(15%) 9/60	1.80e-10
Metastases	(19%) 17/91	(69%) 63/91	2.12e-13

k

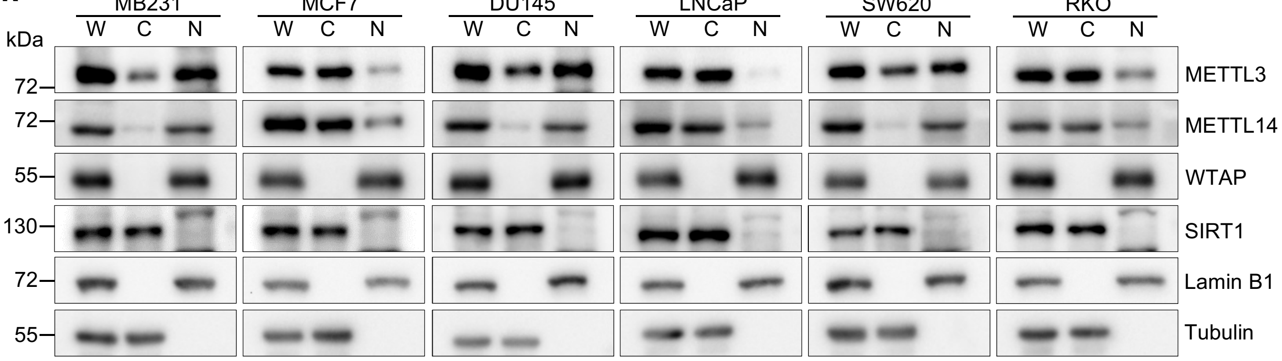

l

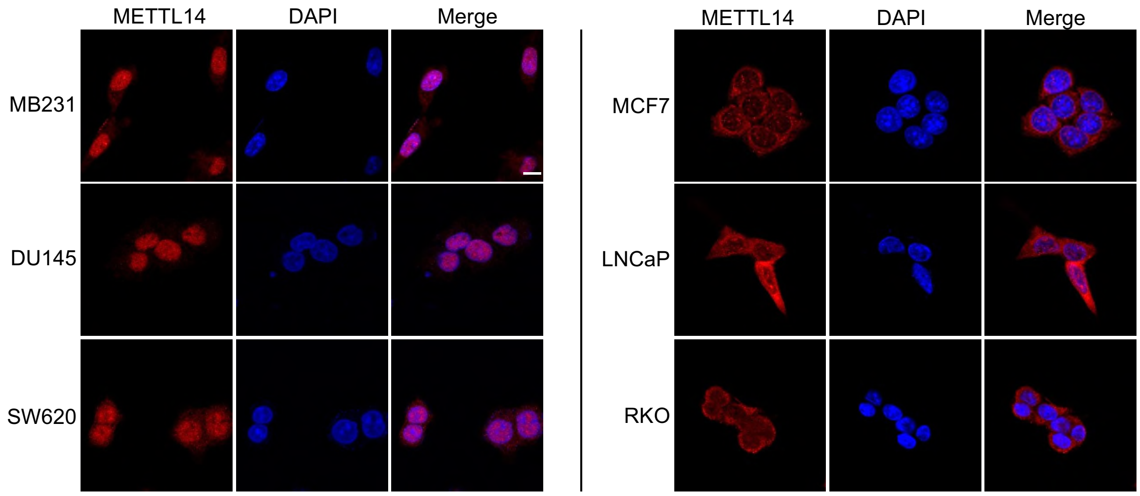

m

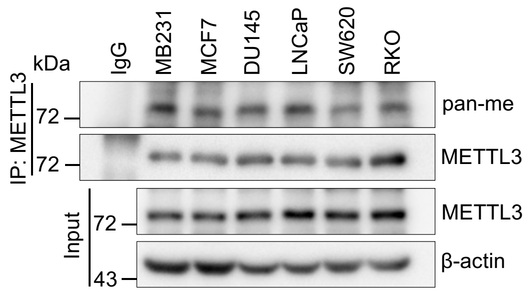

n

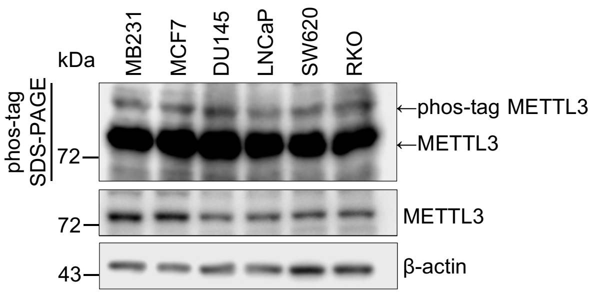

Supplementary Fig. 1 METTL3 nuclear accumulation correlates with node metastasis and invasiveness of human cancers. a,b, Quantification of METTL3 mean intensity in Figure 1 **a,b**. Boxplots are represented as first and third quartiles with a median in the center. Whiskers are defined as 1.5 times the interquartile range. Grade I: non-metastases n = 30, metastases n = 63; Grade II: non-metastases n = 105, metastases n = 48; Grade III: non-metastases n = 25, metastases n = 20. Luminal: non-metastases n = 12, metastases n = 24; HER2+: non-metastases n = 9, metastases n = 11; Basal like: non-metastases n = 7, metastases n = 7. From down to up **a**: ns P = 0.11, ns P = 0.39, ns P = 0.73, respectively, by two-sided t-test. From down to up **b**: ns P = 0.39, ns P = 0.91, ns P = 0.41, respectively, by two-sided t-test. **c,** Representative images of METTL3 IHC staining in normal breast tissues. Scale bars, 100 µm for low magnification (10 ×), and 25 µm for high magnification (40 ×). Normal breast tissues: n = 41. **d,** Representative images of METTL3 IHC staining in the indicated breast carcinomas and adjacent normal tissues. Scale bars, 1 mm for low magnification (1 ×) and 25 µm for high magnification (40 ×). Non-metastases n = 30, metastases n = 30. **e,** Representative images of WTAP, RBM15, KIAA1429 and METTL14 IHC staining in the indicated breast carcinomas. Scale bars, 100 µm for low magnification (10 ×, left panel), and 25 µm for high magnification (40 ×, right panel). Normal breast tissues: n = 41, non-metastases n = 160, metastases n = 131. **f,** Representative images and quantification of METTL3 IHC staining in paired primary and lymphatic metastatic breast cancer samples. Scale bars, 100 µm for low magnification (10 ×, left panel), and 25 µm for high magnification (40 ×, right panel). Quantification of METTL3 mean intensity on the lower panel. **g,h,** Representative images (**g**) and quantification (**h**) of METTL3 IHC staining in

the indicated prostate carcinomas. Scale bars, 50 μm for low magnification (20 ×, left panel), and 25 μm for high magnification (40 ×, right panel). Normal breast tissues: *n* = 43, the other sample numbers are shown in **h**. *P* values by two-sided *t*-test. **i,j,** Representative images (**i**) and quantification (**j**) of METTL3 IHC staining in the indicated colon carcinomas. Scale bars, 50 μm for low magnification (20 ×, left panel), and 25 μm for high magnification (40 ×, right panel). Normal breast tissues: *n* = 89, the other sample numbers are shown in **j**. *P* values by two-sided *t*-test. **k,** IB analysis of whole cell lysate (W), cytoplasmic (C) and nuclear (N) fractions of the indicated tumor cell lines. **l,** Representative immunofluorescence for METTL14 (red) and DAPI (blue, cell nuclei) in the indicated cell lines (*n* = 3 independent experiments). Scale bars, 10 μm. **m,** Lysates of the indicated tumor cell lines were subjected to IP and IB analysis. **n,** Lysates of the indicated tumor cell lines were subjected to SDS-PAGE or phospho-tag SDS-PAGE. Source data are provided as a Source Data file.

Supplementary Figure 2

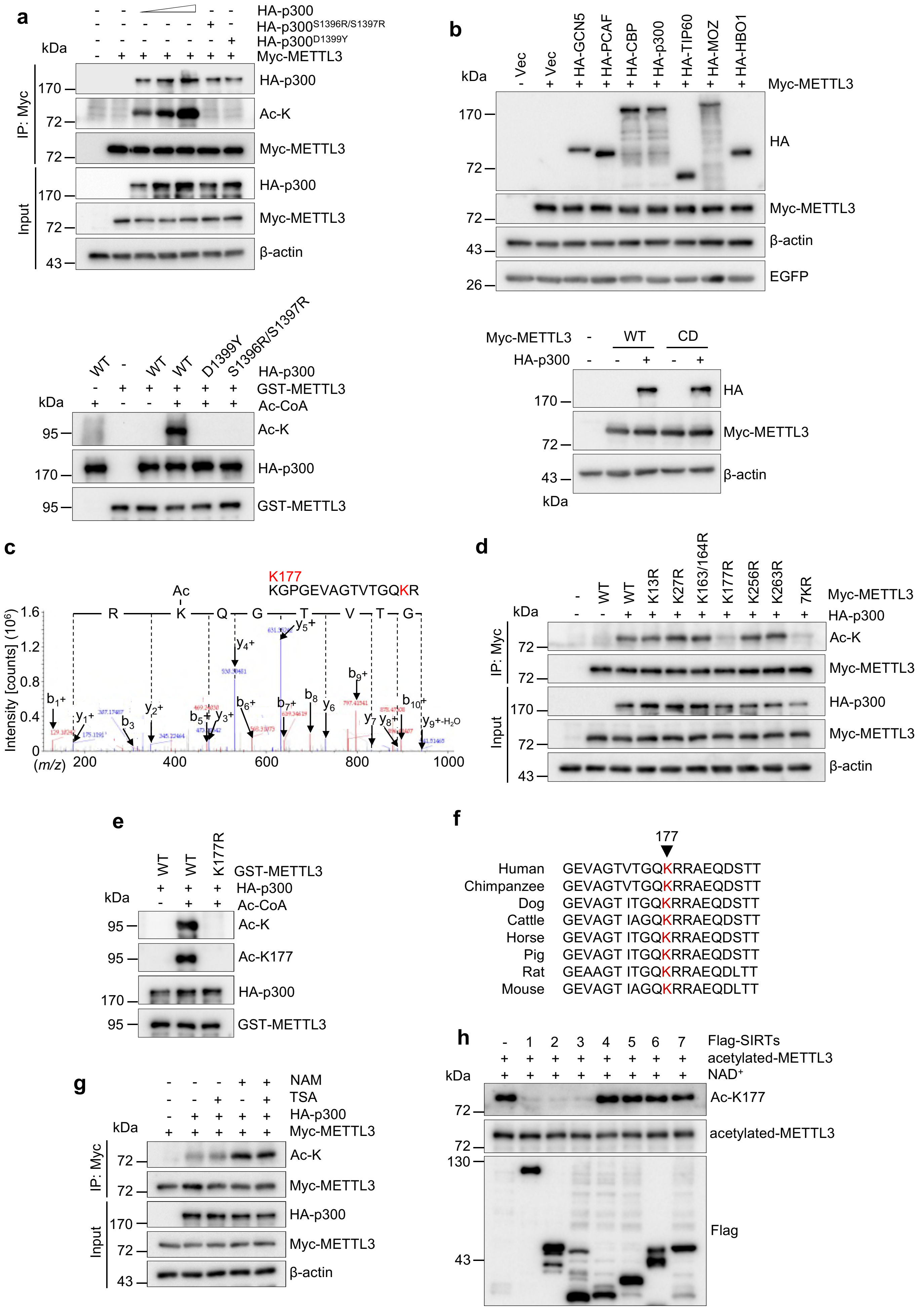

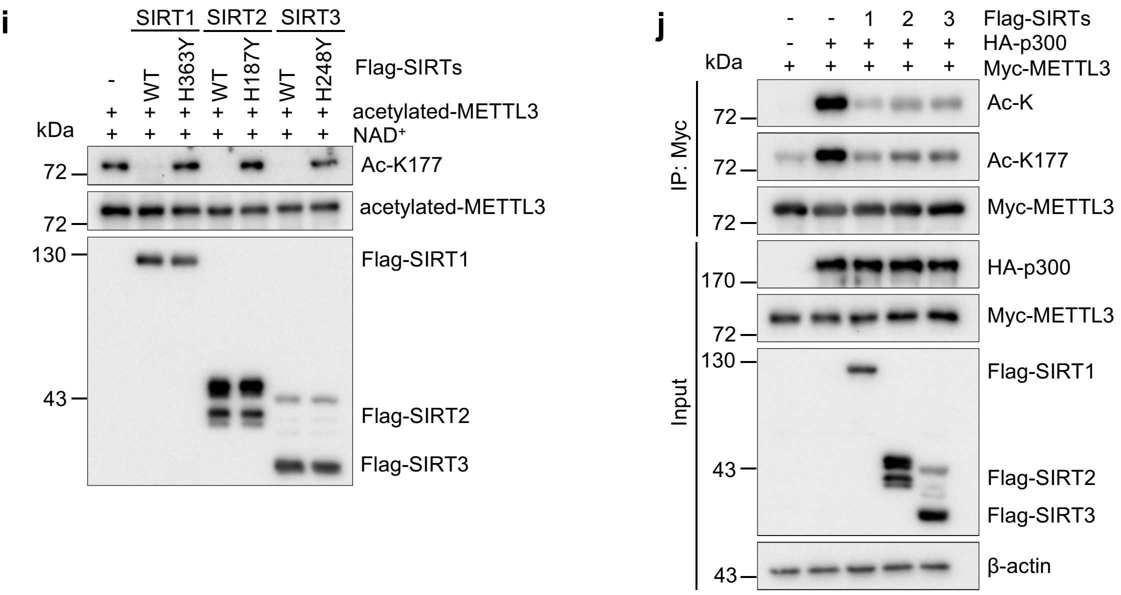

Supplementary Fig. 2 p300-mediated acetylation of METTL3 at K177 is antagonized via SIRT1-dependent deacetylation process. a, Lysates of HEK293T cells transfected with the indicated constructs were subjected to IP and IB analysis (upper panel). Bacterially expressed METTL3 was incubated with p300 or its inactive mutant (expressed and immunoprecipitated from HEK293T cells) in the presence or absence of acetyl-CoA (Ac-CoA) and immunoblotted (lower panel). **b,** MCF-7 cells stably expressing Myc-METTL3 were transfected with the indicated constructs encoding acetyltransferases, followed by IB analysis. **c,** Identification of K177 acetylation on METTL3 by SILAC (stable isotope labeling by amino acids in cell culture) analysis. The spectrum of the acetylated peptide KGPGEVAGTVTGQK(Ac)R is shown. The partial amino acid sequence (from left to right, C-terminal to N-terminal) deduced from y ions is shown on the spectrum. The mass difference between y3 and y2 ions is the mass of the acetyl-modified lysine residue. **d,** Lysates of HEK293T cells transfected with the indicated constructs were subjected to IP and IB analysis. **e,** Bacterially expressed METTL3 or its mutant was incubated with p300 in the presence or absence of Ac-CoA and immunoblotted. **f,** Sequences alignment of the residues flanking Lys177 across different species. Arrowhead points to the Lys177 residue corresponding to human METTL3. **g,** HEK293T cells transfected with the indicated constructs were treated with trichostatin A (TSA, 2 μM) or nicotinamide (NAM, 3 mM) for 6 h, followed by IP and IB analysis. **h,i,** Acetylated-METTL3 was incubated with Flag-tagged SIRTs or the inactive mutant (expressed and immunoprecipitated from HEK293T cells) in the presence of NAD^+ for 1 h, followed by immunoblotting. **j,** Lysates of HEK293T cells transfected with the indicated constructs were subjected to IP and IB analysis. Source data are provided as a Source Data file.

Supplementary Figure 3

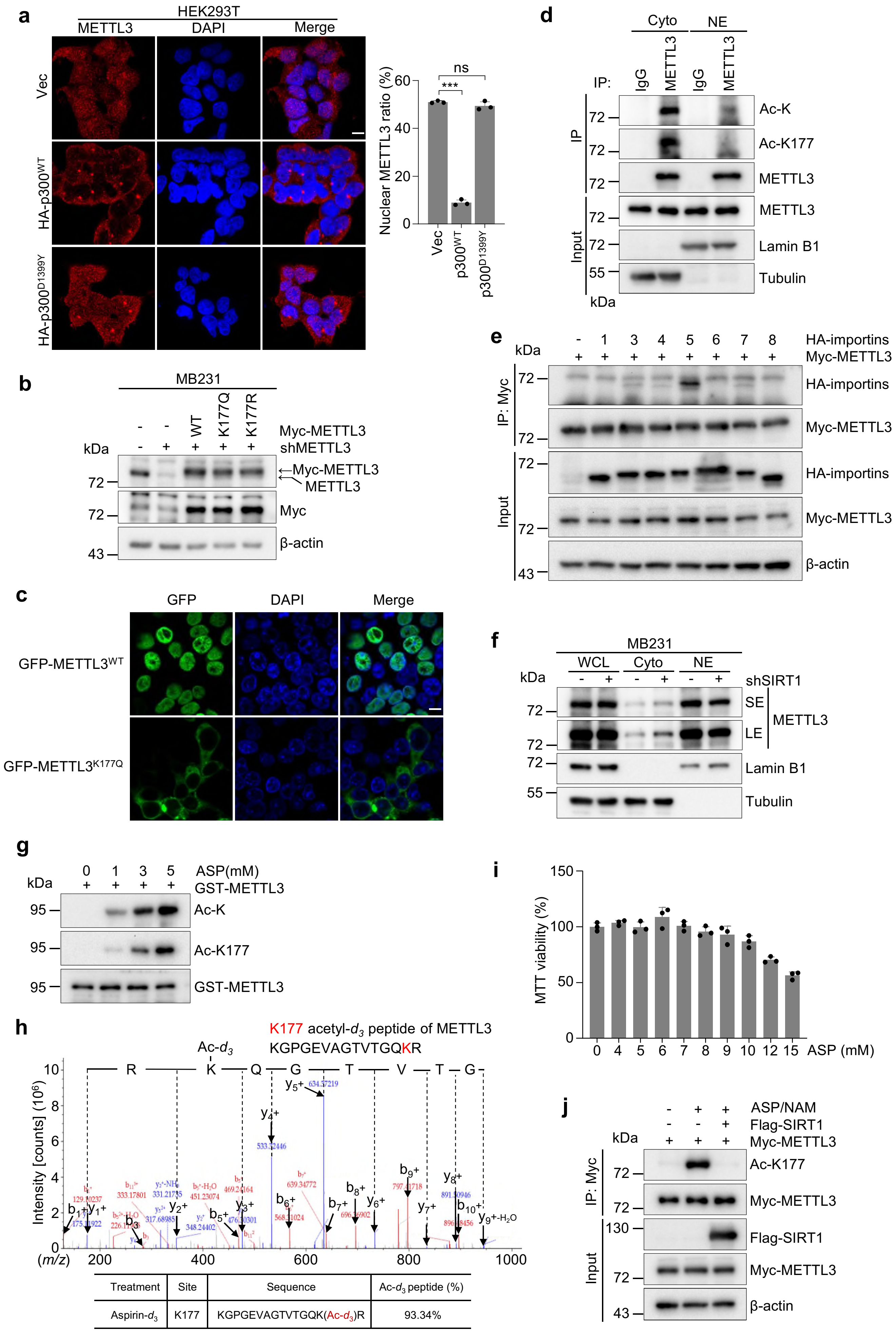

Treatment	Site	Sequence	Ac-d_3 peptide (%)
Aspirin-d_3	K177	KGPGEVAGTVTGQK(Ac-d_3)R	93.34%

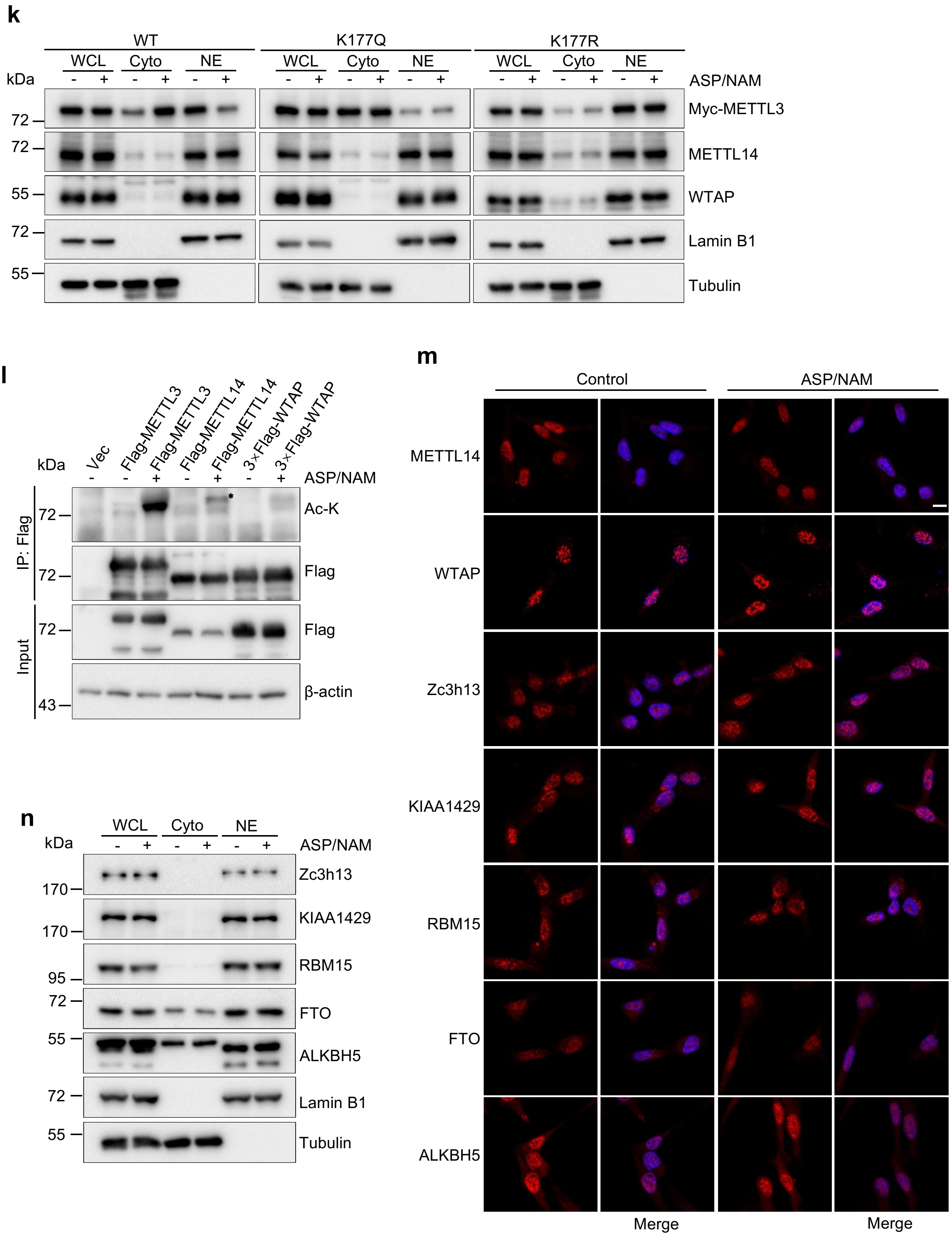

k
WT
K177Q
K177R
WCL
Cyto
NE
kDa
ASP/NAM
Myc-METTL3
METTL14
WTAP
Lamin B1
Tubulin
72
72
55
72
55
l
kDa
Vec
Flag-METTL3
Flag-METTL3
Flag-METTL14
Flag-METTL14
3×Flag-WTAP
3×Flag-WTAP
ASP/NAM
IP: Flag
Input
Ac-K
Flag
Flag
β-actin
72
72
72
43
m
Control
ASP/NAM
METTL14
WTAP
Zc3h13
KIAA1429
RBM15
FTO
ALKBH5
Merge
Merge
n
WCL
Cyto
NE
kDa
ASP/NAM
Zc3h13
KIAA1429
RBM15
FTO
ALKBH5
Lamin B1
Tubulin
170
170
95
72
55
72
55

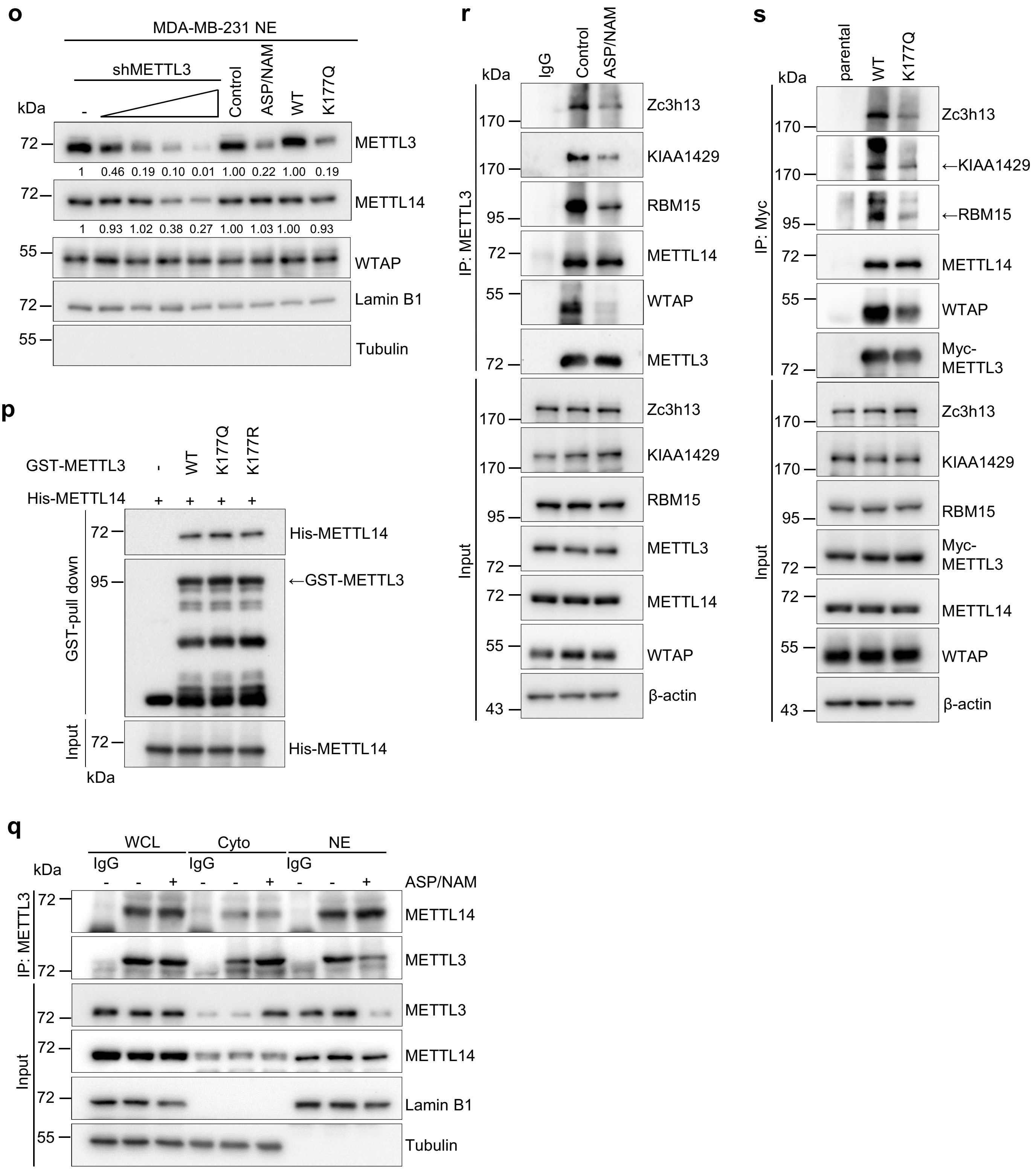
o
MDA-MB-231 NE
shMETTL3
kDa
-
Control
ASP/NAM
WT
K177Q
72
METTL3
1 0.46 0.19 0.10 0.01 1.00 0.22 1.00 0.19
72
METTL14
1 0.93 1.02 0.38 0.27 1.00 1.03 1.00 0.93
55
WTAP
72
Lamin B1
55
Tubulin
p
GST-METTL3 - WT K177Q K177R
His-METTL14 + + + +
GST-pull down
72
His-METTL14
95
←GST-METTL3
Input
72
His-METTL14
kDa
q
WCL
Cyto
NE
kDa
IgG
IgG
IgG
- - + - - + - - +
ASP/NAM
IP: METTL3
72
METTL14
72
METTL3
Input
72
METTL3
72
METTL14
72
Lamin B1
55
Tubulin
r
kDa
IgG
Control
ASP/NAM
IP: METTL3
170
Zc3h13
170
KIAA1429
95
RBM15
72
METTL14
55
WTAP
72
METTL3
Input
170
Zc3h13
170
KIAA1429
95
RBM15
72
METTL3
72
METTL14
55
WTAP
43
β-actin
s
kDa
parental
WT
K177Q
IP: Myc
170
Zc3h13
170
←KIAA1429
95
←RBM15
72
METTL14
55
WTAP
72
Myc-METTL3
Input
170
Zc3h13
170
KIAA1429
95
RBM15
72
Myc-METTL3
72
METTL14
55
WTAP
43
β-actin

Supplementary Fig. 3 ASP synergizes with SIRT1 inhibition in promoting METTL3 acetylation and cytosolic retention. a, Immunofluorescence with anti-METTL3 antibody (red) and DAPI staining (blue, cell nuclei) of HEK293T cells transfected with the indicated constructs (left panel) (n = 3 biologically independent experiments). Scale bars, 10 μm. Quantification of nuclear METTL3 percentage is presented as mean ± SD (right panel). *** P = 7.28e-07, ns P = 0.22 by two-sided t-test. **b,** Lysates of MDA-MB-231 cells infected with the indicated lentiviruses were subjected to IB analysis. **c,** Representative images for GFP and DAPI (blue, cell nuclei) in 293T cells transfected with the indicated METTL3 constructs (n = 3 independent experiments). Scale bars, 10 μm. **d,** IP and IB analysis of cytoplasmic and nuclear fractions of MCF-7 cells as indicated. **e,** Lysates of HEK293T cells transfected with the indicated constructs were subjected to IP and IB analysis. **f,** IB analysis of WCL, Cyto and NE fractions of MDA-MB-231 cells infected with the indicated lentiviruses. **g,** Incubation of recombinant METTL3 protein with ASP, followed by IB analysis of acetylation level of METTL3 with pan-Ac-K or Ac-K177 antibodies. **h,** Cells were treated with ASP-d_3 (5 mM)/NAM for 24 hours, and Myc-METTL3 was immunoprecipitated. The percentage of acetyl-d_3-K177 METTL3 (lower) and the representative K177 acetylated peptide of METTL3 with acetyl-d_3 (upper) were analyzed by Thermo Scientific Q Exactive HF Hybrid Quadrupole-Orbitrap mass spectrometer. y ion peaks, blue; b ion peaks, red. The mass difference between y3 and y2 ions is the mass of the acetyl-d_3-modified lysine residue. **i,** Cell viability of MDA-MB-231 cells treated with 3mM NAM plus varying amount of ASP were analyzed by MTT assay. Data are mean ± SEM from triplicates. **j,** HEK293T cells transfected with the indicated constructs were treated with ASP/NAM. Lysates were subjected to IP and IB analysis. **k,** IB analysis of WCL, Cyto and NE fractions from the indicated METTL3 reconstituting MDA-MB-231 cells treated with ASP/NAM**. l,** HEK293T cells transfected with the indicated constructs were treated with ASP/NAM. Lysates were subjected to IP and IB analysis. Asterisk indicates a non-specific band. **m,** Representative immunofluorescence for METTL14, WTAP, Zc3h13, KIAA1429, RBM15, FTO and ALKBH5 (red) in MDA-MB-231 cells treated with ASP/NAM (n = 3 independent experiments). DAPI (blue, cell nuclei), scale bars, 10 μm. **n,** IB analysis of WCL, Cyto and NE fractions from MDA-MB-231 cells treated with ASP/NAM. **o,** IB analysis of NE fractions from the indicated MDA-MB-231 cells subjected to the indicated treatment. Quantitative densitometry analysis was performed using Image Lab 5.2 software (Bio-Rad). **p,** Purified METTL14 recombinant proteins were incubated with GST or GST-METTL3. Proteins retained on sepharose were then blotted with the indicated antibodies. **q,** IP and IB analysis of WCL, Cyto and NE fractions from MDA-MB-231 cells treated with ASP/NAM. **r,** Lysates of MDA-MB-231 cells treated with ASP/NAM were subjected to IP and IB analysis. **s,** Lysates of METTL3 reconstituting MDA-MB-231 cells were subjected to IP and IB analysis.

In this figure, cells were treated with 5 mM ASP and/or 3 mM NAM for 24 hours, unless otherwise indicated. Source data are provided as a Source Data file.

Supplementary Figure 4

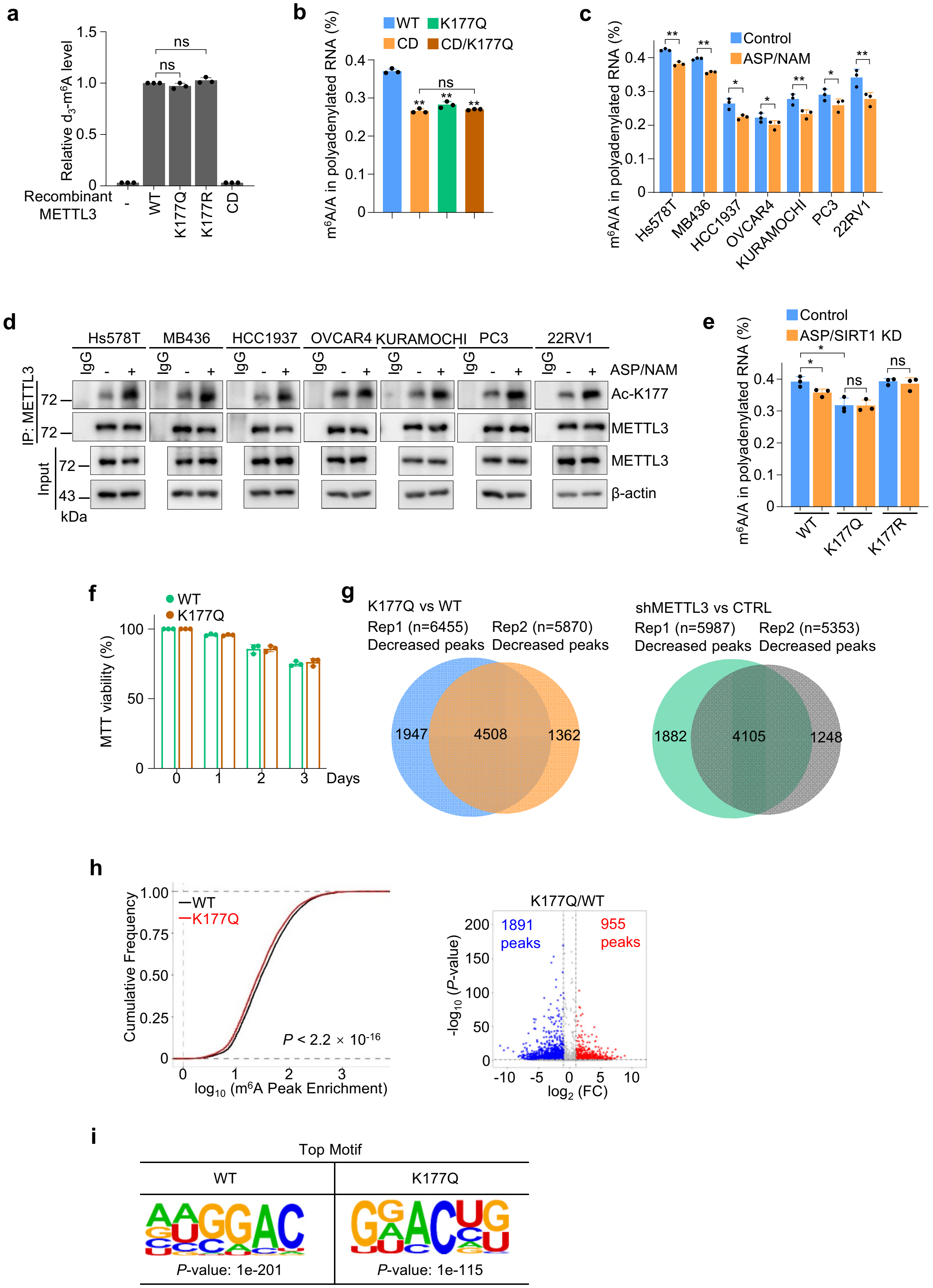

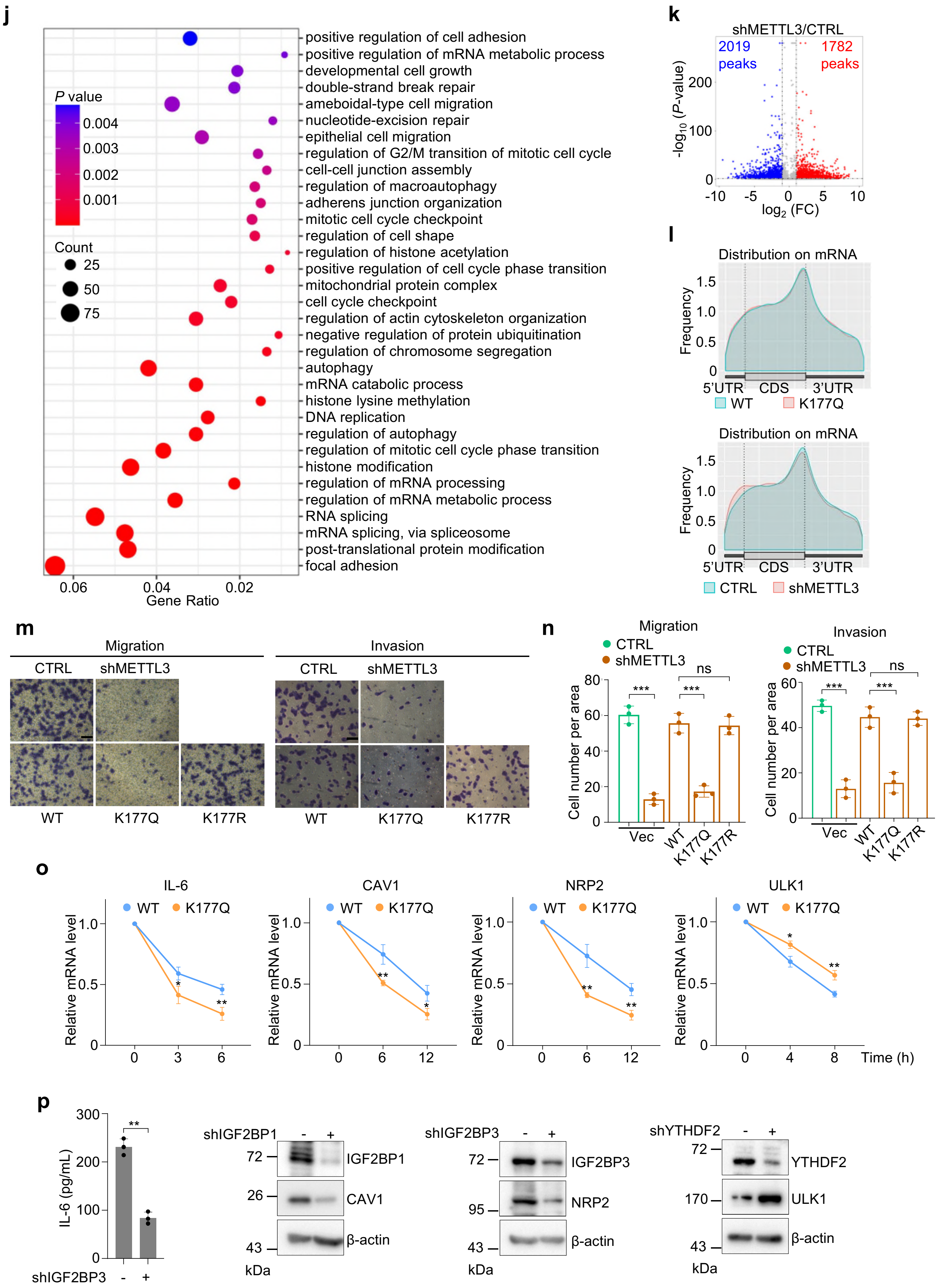

j
P value
0.004
0.003
0.002
0.001
Count
25
50
75
positive regulation of cell adhesion
positive regulation of mRNA metabolic process
developmental cell growth
double-strand break repair
ameboidal-type cell migration
nucleotide-excision repair
epithelial cell migration
regulation of G2/M transition of mitotic cell cycle
cell-cell junction assembly
regulation of macroautophagy
adherens junction organization
mitotic cell cycle checkpoint
regulation of cell shape
regulation of histone acetylation
positive regulation of cell cycle phase transition
mitochondrial protein complex
cell cycle checkpoint
regulation of actin cytoskeleton organization
negative regulation of protein ubiquitination
regulation of chromosome segregation
autophagy
mRNA catabolic process
histone lysine methylation
DNA replication
regulation of autophagy
regulation of mitotic cell cycle phase transition
histone modification
regulation of mRNA processing
regulation of mRNA metabolic process
RNA splicing
mRNA splicing, via spliceosome
post-translational protein modification
focal adhesion
0.06
0.04
0.02
Gene Ratio
k
shMETTL3/CTRL
2019 peaks
1782 peaks
-log10 (P-value)
log2 (FC)
l
Distribution on mRNA
Frequency
5'UTR
CDS
3'UTR
WT
K177Q
Distribution on mRNA
CTRL
shMETTL3
m
Migration
Invasion
CTRL
shMETTL3
WT
K177Q
K177R
n
Migration
Invasion
CTRL
shMETTL3
ns
***
Cell number per area
Vec
WT
K177Q
K177R
o
IL-6
CAV1
NRP2
ULK1
WT
K177Q
Relative mRNA level
Time (h)
p
IL-6 (pg/mL)
shIGF2BP3
shIGF2BP1
IGF2BP1
CAV1
β-actin
IGF2BP3
NRP2
shYTHDF2
YTHDF2
ULK1
kDa

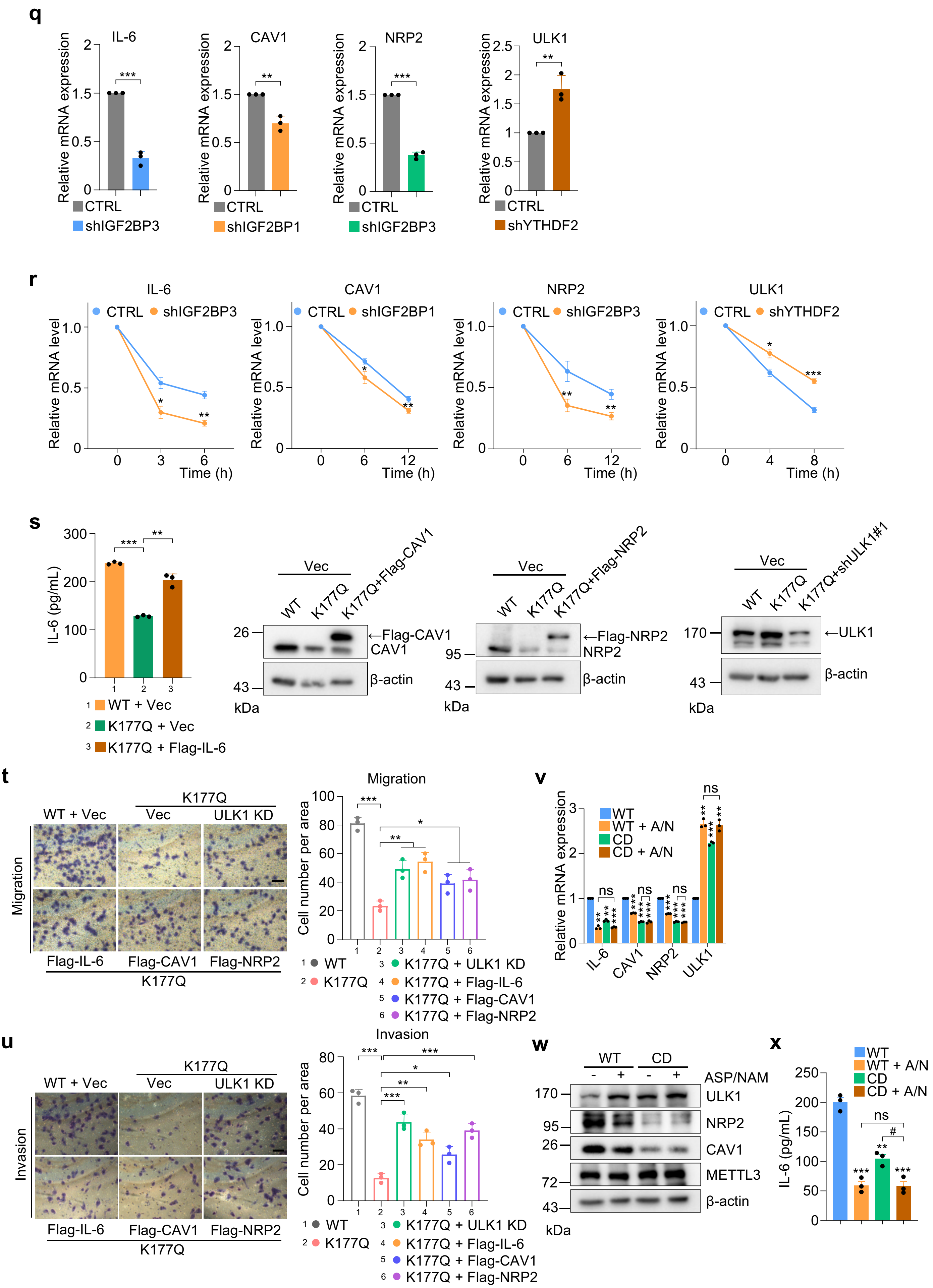

q
IL-6
CAV1
NRP2
ULK1
Relative mRNA expression
CTRL
shIGF2BP3
shIGF2BP1
shYTHDF2
r
Relative mRNA level
Time (h)
s
IL-6 (pg/mL)
WT + Vec
K177Q + Vec
K177Q + Flag-IL-6
Vec
WT
K177Q
K177Q+Flag-CAV1
K177Q+Flag-NRP2
K177Q+shULK1#1
←Flag-CAV1
CAV1
β-actin
←Flag-NRP2
NRP2
←ULK1
kDa
t
Migration
WT + Vec
K177Q
Vec
ULK1 KD
Flag-IL-6
Flag-CAV1
Flag-NRP2
Cell number per area
WT
K177Q
K177Q + ULK1 KD
K177Q + Flag-IL-6
K177Q + Flag-CAV1
K177Q + Flag-NRP2
v
WT
WT + A/N
CD
CD + A/N
ns
u
Invasion
w
ASP/NAM
METTL3
x

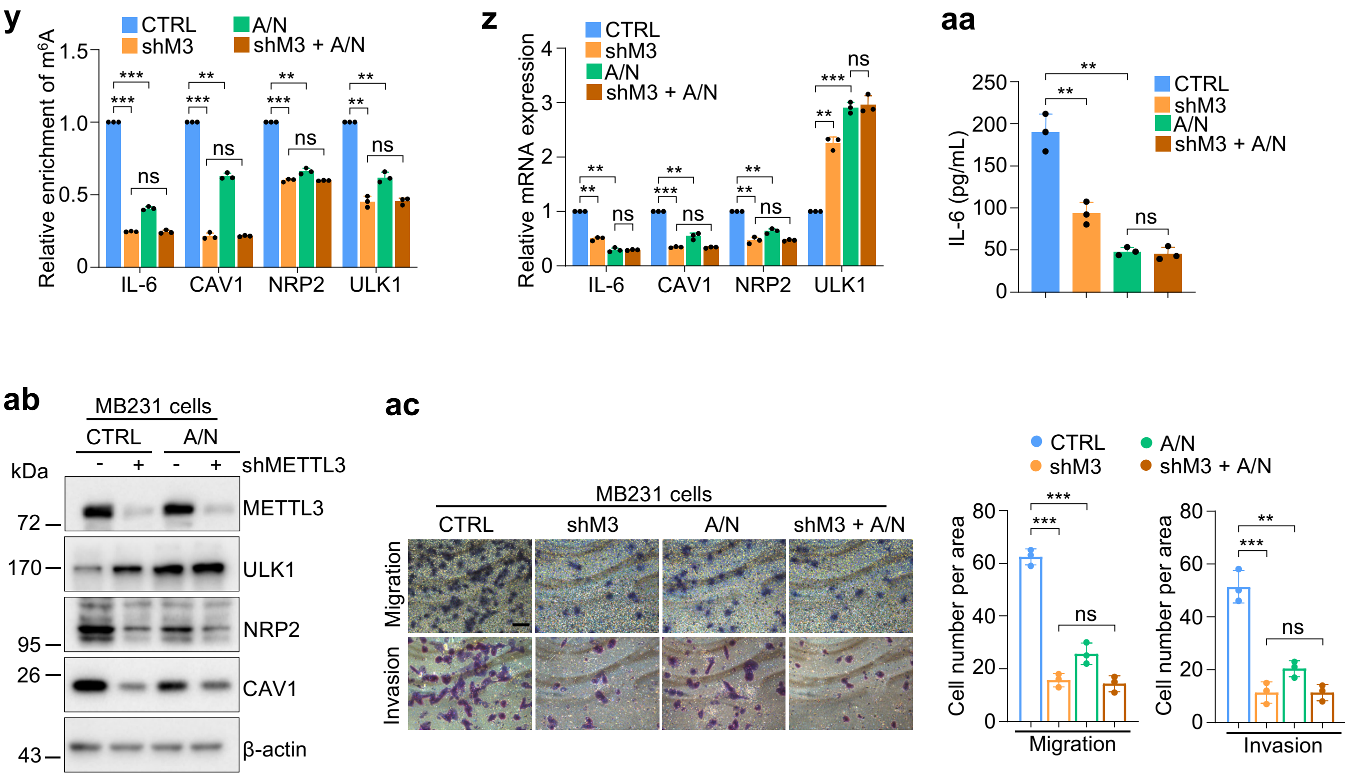

Supplementary Fig. 4 ASP/NAM represses METTL3-dependent m^{6}A modification via promoting METTL3 acetylation at K177. a, In vitro methylation activities of METTL3 or its K177 mutants were analyzed using a published RNA probe as described in the Methods with the consensus GGACU motif in the presence of isotope-labeled cofactor d_3-SAM. The methylation yields were calculated on the basis of the molar ratio of d_3-m^{6}A to specific probe, measured by LC-MS/MS (n = 3 biologically independent experiments). From left to right: ns P = 0.16, ns P = 0.19, respectively, by two-sided t-test. **b,** LC-MS/MS quantification of the m^{6}A/A ratio in polyadenylated RNA isolated from the indicated METTL3 reconstituting MDA-MB-231 cells (n = 3 biologically independent experiments). From left to right: ** P = 0.003, ** P = 0.006, ** P = 0.0012, ns P = 0.47, respectively, by two-sided t-test. **c,** LC-MS/MS quantification of the m^{6}A/A ratio in polyadenylated RNA isolated from the indicated tumor cell lines treated with ASP/NAM (n = 3 biologically independent experiments). From left to right: ** P = 0.002, ** P = 0.005, * P = 0.017, * P = 0.047, ** P = 0.003, * P = 0.035, ** P = 0.003, respectively, by two-sided t-test. **d,** Lysates of cell lines in (**c**) were subjected to IP and IB analysis. **e,** LC-MS/MS quantification of the m^{6}A/A ratio in polyadenylated RNA isolated from METTL3 reconstituting MDA-MB-231 cells infected with the indicated lentiviruses and treated with ASP (n = 3 biologically independent experiments). From left to right: * P = 0.022, * P = 0.015, ns P = 0.73, ns P = 0.32, respectively, by two-sided t-test. **f,** Cell viability of METTL3 reconstituting MDA-MB-231 cells treated with ASP/NAM at the indicated time window was analyzed by MTT assay. Error bars indicate mean ± SEM from triplicates. **g,** Venn diagram showing overlaps of m^{6}A peaks acquired from replicates indicated high reproducibility. **h,** Cumulative distribution of differential m^{6}A peak intensity in METTL3^{WT} and METTL3^{K177Q} reconstituting MDA-MB-231 cells (left panel). Volcano plot for METTL3^{K177Q} versus METTL3^{WT} reconstituting MDA-MB-231 cells, showing peaks with differential m^{6}A intensity. Fold change (FC) is the ratio of IP over input for METTL3^{K177Q} and METTL3^{WT} (right panel). **i,** Sequence motif identified among top 1000

m^{6}A peaks in METTL3 reconstituting MDA-MB-231 cells. **j,** Gene ontology enrichmentanalysis of differentially m^{6}A-modified mRNAs between METTL3^{WT} and METTL3^{K177Q} reconstituting MDA-MB-231 cells. **k,** Volcano plot for METTL3-deficient versus CTRL MDA-MB-231 cells, showing peaks with differential m^{6}A intensity. Fold change (FC) is the ratio of IP over input for METTL3-deficient and CTRL cells. **l,** Metagene plots showing the distribution of m^{6}A peaks identified across mRNAs in METTL3^{WT} and METTL3^{K177Q} reconstituting MDA-MB-231 cells (upper panel), the distribution of m^{6}A peaks identified across mRNAs in CTRL and METTL3-deficient MDA-MB-231 cells (lower panel). **m,n,** Migration and invasion assays were performed in the indicated MDA-MB-231 cells treated with ASP/NAM (**m**) (n = 3 biologically independent experiments). Scale bars, 50 μm. Quantifications are shown in **n**. From left to right for migration: *** $P = 0.0002$, *** $P = 0.0005$, ns $P = 0.77$; from left to right for invasion: *** $P = 0.0002$, *** $P = 0.0014$, ns $P = 0.84$, respectively, by two-sided t-test. **o,** Relative levels of the indicated mRNAs were measured by QRT-PCR in METTL3 reconstituting MDA-MB-231 cells treated with actinomycin D for the indicated times (n = 3 biologically independent experiments). IL-6: * $P = 0.026$, ** $P = 0.0072$; CAV1: ** $P = 0.0082$, * $P = 0.02$; NRP2: ** $P = 0.0045$, ** $P = 0.0049$; ULK1: * $P = 0.011$, ** $P = 0.0042$, respectively, by two-sided t-test. **p,** Measurement of IL-6 levels by ELISA and IB analysis of the indicated proteins in MDA-MB-231 cells infected with the indicated lentiviruses (n = 3 biologically independent experiments). IL-6: ** $P = 0.0043$ by two-sided t-test. **q,** QRT-PCR quantification of the indicated mRNAs in MDA-MB-231 cells infected with the indicated lentiviruses (n = 3 biologically independent experiments). IL-6: *** P = 7.56e-05; CAV1: ** $P = 0.0023$; NRP2: *** P = 7.66e-06; ULK1: ** $P = 0.0053$, respectively, by two-sided t-test. **r,** Relative levels of the indicated mRNAs measured by QRT-PCR in MDA-MB-231 cells infected with the indicated lentiviruses, followed by 5 μg/mL actinomycin D treatment for the indicated times (n = 3 biologically independent experiments). IL-6: * $P = 0.011$, ** $P = 0.0027$; CAV1: * $P = 0.013$, ** $P = 0.0061$; NRP2: ** $P = 0.0077$, ** $P = 0.0039$; ULK1: * $P = 0.012$, *** $P = 0.0007$, respectively, by two-sided t-test. **s,** IL-6 levels were measured by ELISA from the supernatants of the indicated METTL3 reconstituting MDA-MB-231 cells infected with the indicated lentiviruses (left panel) (n = 3 biologically independent experiments). *** P = 6.86e-07, ** $P = 0.0087$, respectively, by two-sided t-test. IB analysis of the indicated proteins in METTL3 reconstituting MDA-MB-231 cells infected with the indicated lentiviruses (right panel). **t,u,** Migration (**t**) and invasion (**u**) assays were conducted using METTL3 reconstituting MDA-MB-231 cells infected with the indicated lentiviruses (left panel) (n = 3 biologically independent experiments). Scale bars, 50 μm. Quantifications are shown on the right panel. From left to right for migration: *** P = 6.55e-05, ** $P = 0.0039$, ** $P = 0.002$, * $P = 0.022$, * $P = 0.019$; from left to right for invasion: *** P = 6.41e-05, *** $P = 0.0006$, ** $P = 0.0018$, * $P = 0.012$, *** $P = 0.0006$, respectively, by two-sided t-test. **v,** Relative mRNA levels of the indicated genes were measured by QRT-PCR analysis in METTL3 reconstituting MDA-MB-231 cells treated with ASP/NAM (n = 3 biologically independent experiments). From left to right: ** $P = 0.0012$, ** $P = 0.0011$, *** $P = 0.0001$, ns $P = 0.82$, *** $P = 0.0002$, *** $P = 0.0002$, *** $P = 0.0008$, ns $P = 0.92$, *** $P = 0.0001$, *** $P = 0.0003$, *** $P = 0.0001$, ns $P = 0.14$, ** $P = 0.0012$, *** $P = 0.0004$, *** $P = 0.0019$, ns $P = 0.43$, respectively, by two-sided t-test. **w,** IB analysis of the indicated proteins in METTL3 reconstituting MDA-MB-231 cells treated with ASP/NAM. **x,** Measurement of IL-6 levels by ELISA in METTL3 reconstituting MDA-MB-231 cells treated with ASP/NAM (n = 3 biologically independent experiments). From left to right: *** $P = 0.0004$, ** $P = 0.0017$, *** $P = 0.0008$, ns $P = 0.91$, $^{\#}$ $P = 0.024$, respectively, by

two-sided *t*-test. * compared to $METTL3^{WT}$. # compared to $METTL3^{CD}$. **y,** m^6A-MeRIP-qPCR analysis of the indicated m^6A substrates normalized to input in MDA-MB-231 cells subjected to the indicated treatment (*n* = 3 biologically independent experiments). From left to right: *** *P* = 1.92e-10, *** *P* = 0.0001, ns *P* = 0.81, *** *P* = 0.0003, ** *P* = 0.0011, ns *P* = 0.98, *** *P* = 0.0003, ** *P* = 0.0013, ns *P* = 0.87, ** *P* = 0.0016, ** *P* = 0.0029, ns *P* = 0.79, respectively, by two-sided *t*-test. **z,** QRT-PCR quantification of the indicated mRNAs in MDA-MB-231 cells subjected to the indicated treatment (*n* = 3 biologically independent experiments). From left to right: ** *P* = 0.0015, ** *P* = 0.0011, ns *P* = 0.96, *** *P* = 2.96e-08, ** *P* = 0.0058, ns *P* = 0.42, ** *P* = 0.0045, ** *P* = 0.0054, ns *P* = 0.98, ** *P* = 0.0028, *** *P* = 0.0009, ns *P* = 0.48, respectively, by two-sided *t*-test. **aa,** Measurement of IL-6 levels by ELISA in MDA-MB-231 cells subjected to the indicated treatment (*n* = 3 biologically independent experiments). From left to right: ** *P* = 0.0028, ** *P* = 0.0066, ns *P* = 0.73, respectively, by two-sided *t*-test. **ab,** IB analysis of the indicated proteins in MDA-MB-231 cells subjected to the indicated treatment. **ac,** Migration and invasion assays were conducted using MDA-MB-231 cells subjected to the indicated treatment (left panel) (*n* = 3 biologically independent experiments). Scale bars, 50 μm. Quantification is shown on the right panel. From left to right for migration: *** *P* = 3.40e-05, *** *P* = 0.0002, ns *P* = 0.59; from left to right for invasion: *** *P* = 0.0007, ** *P* = 0.0014, ns *P* = 0.99, respectively, by two-sided *t*-test.

In this figure, cells were treated with 5 mM ASP and/or 3 mM NAM for 72 hours. All data are represented as mean ± SD, unless specially defined. All *P* values were calculated by two-sided Student's *t*-test. Source data are provided as a Source Data file.

Supplementary Figure 5

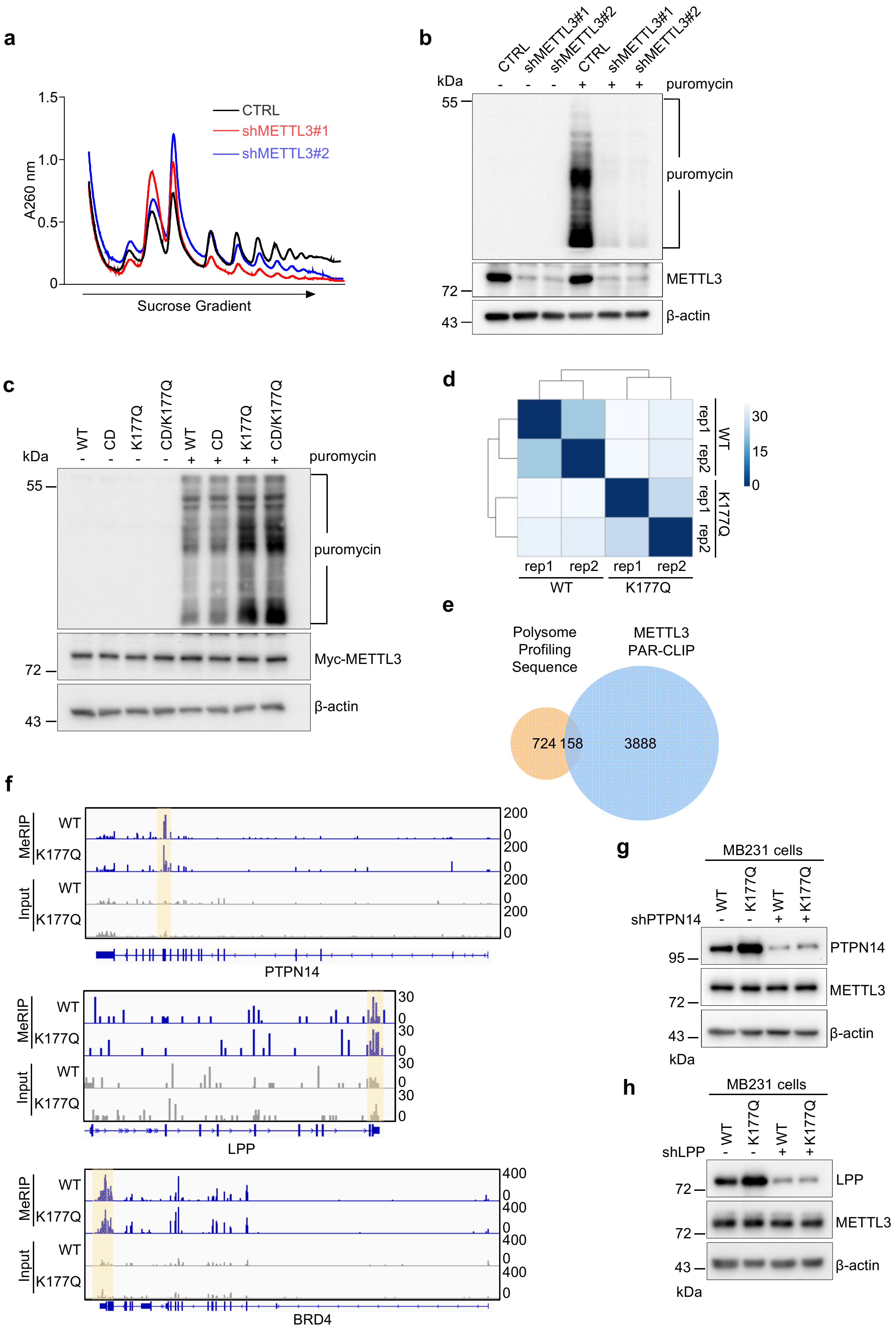

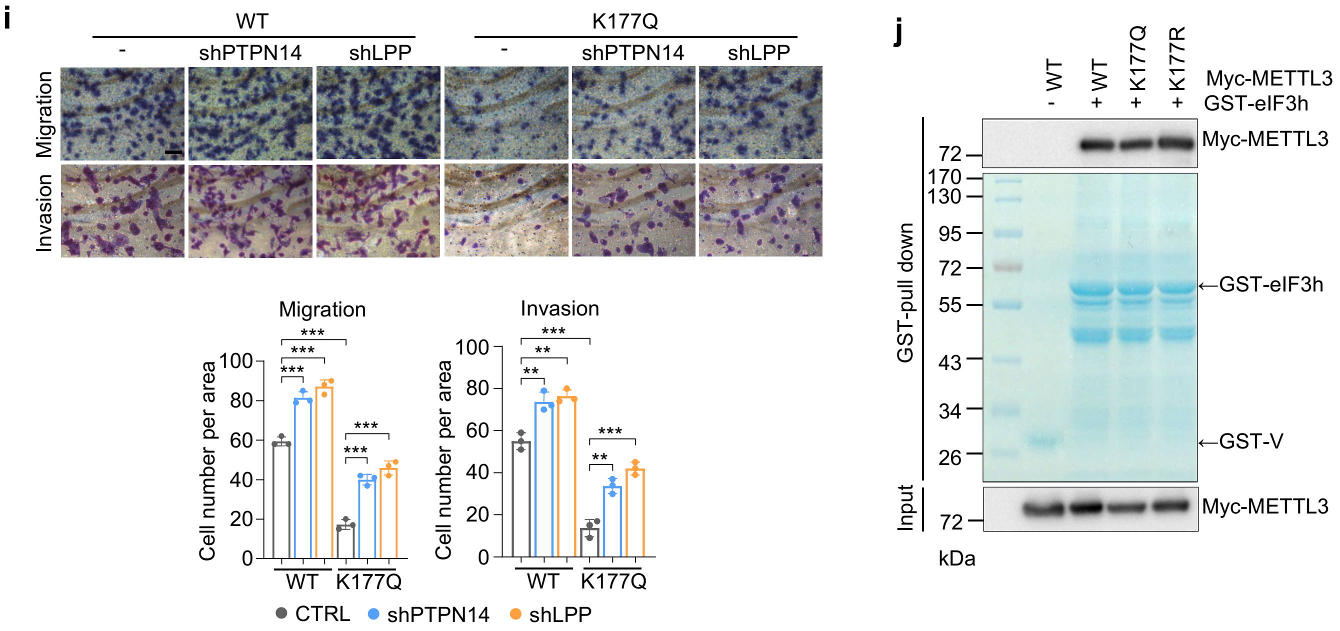

Supplementary Fig. 5 Acetylation-mimetic METTL3^{K177Q} reconstitution promotes protein translation in breast cancer cells. a, Sucrose gradient-based polysome profiling of MDA-MB-231 cells infected with the indicated lentiviruses. Plot is representative of two independently performed experiments with similar results. **b,** SUnSET assays show reduced protein production in METTL3-depleted MDA-MB-231 cells. Cell lysates were extracted and probed the indicated antibodies. **c,** SUnSET assays show protein production in the indicated METTL3 reconstituting MDA-MB-231 cells. Cell lysates were extracted and probed with the indicated antibodies. **d,** Distance matrix of the polysome profiling in replicates of METTL3^{WT} and METTL3^{K177Q} reconstituting MDA-MB-231 cells. **e,** Venn diagram showing mRNAs with a greater than 1.5-fold change in translation efficiency and with METTL3 PAR-CLIP data. **f,** Genomic visualization of the m^{6}A-MeRIP-seq normalized signal in METTL3^{WT} and METTL3^{K177Q} reconstituting MDA-MB-231 cells for the indicated mRNAs. **g,h,** IB analysis of the indicated proteins in METTL3 reconstituting MDA-MB-231 cells infected with the indicated lentiviruses. **i,** Migration and invasion assays were conducted using METTL3 reconstituting MDA-MB-231 cells infected with the indicated lentiviruses (upper panel) (n = 3 biologically independent experiments). Scale bars, 50 µm. Quantification is shown on the lower panel. From left to right for migration: *** $P = 0.0007$, *** $P = 0.0004$, *** $P = 2.87e-05$, *** $P = 0.0004$, *** $P = 0.0004$; from left to right for invasion: ** $P = 0.0064$, ** $P = 0.002$, *** $P = 0.0002$, ** $P = 0.0031$, *** $P = 0.0007$, respectively, by two-sided t-test. **j,** Lysates of HEK293T cells transfected with the indicated Myc-METTL3 constructs were incubated with recombinant GST-eIF3h. Proteins retained on sepharose were then blotted with the indicated antibodies. All data are represented as mean ± SD, unless specially defined. All P values were calculated by Student's t-test. Source data are provided as a Source Data file.

Supplementary Figure 6

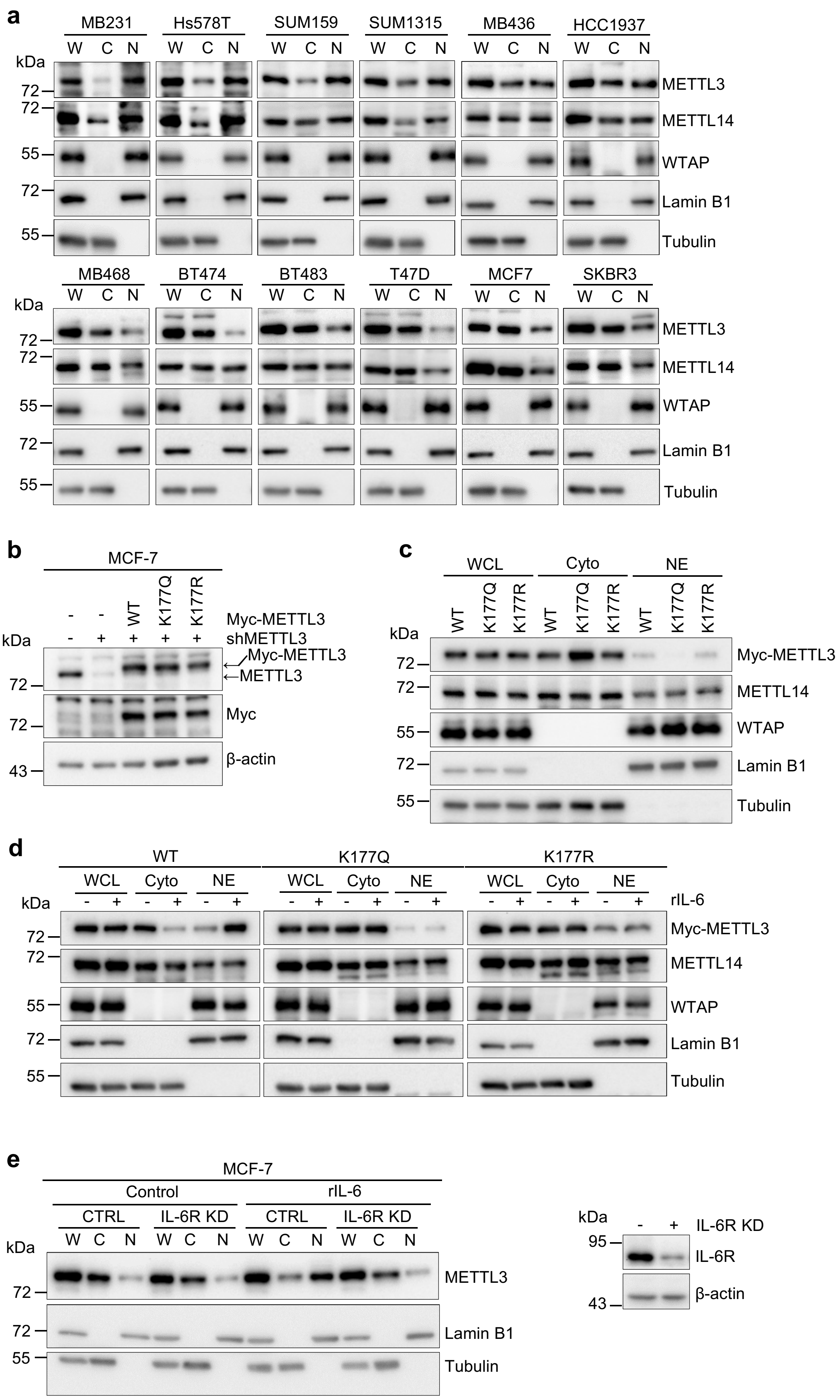

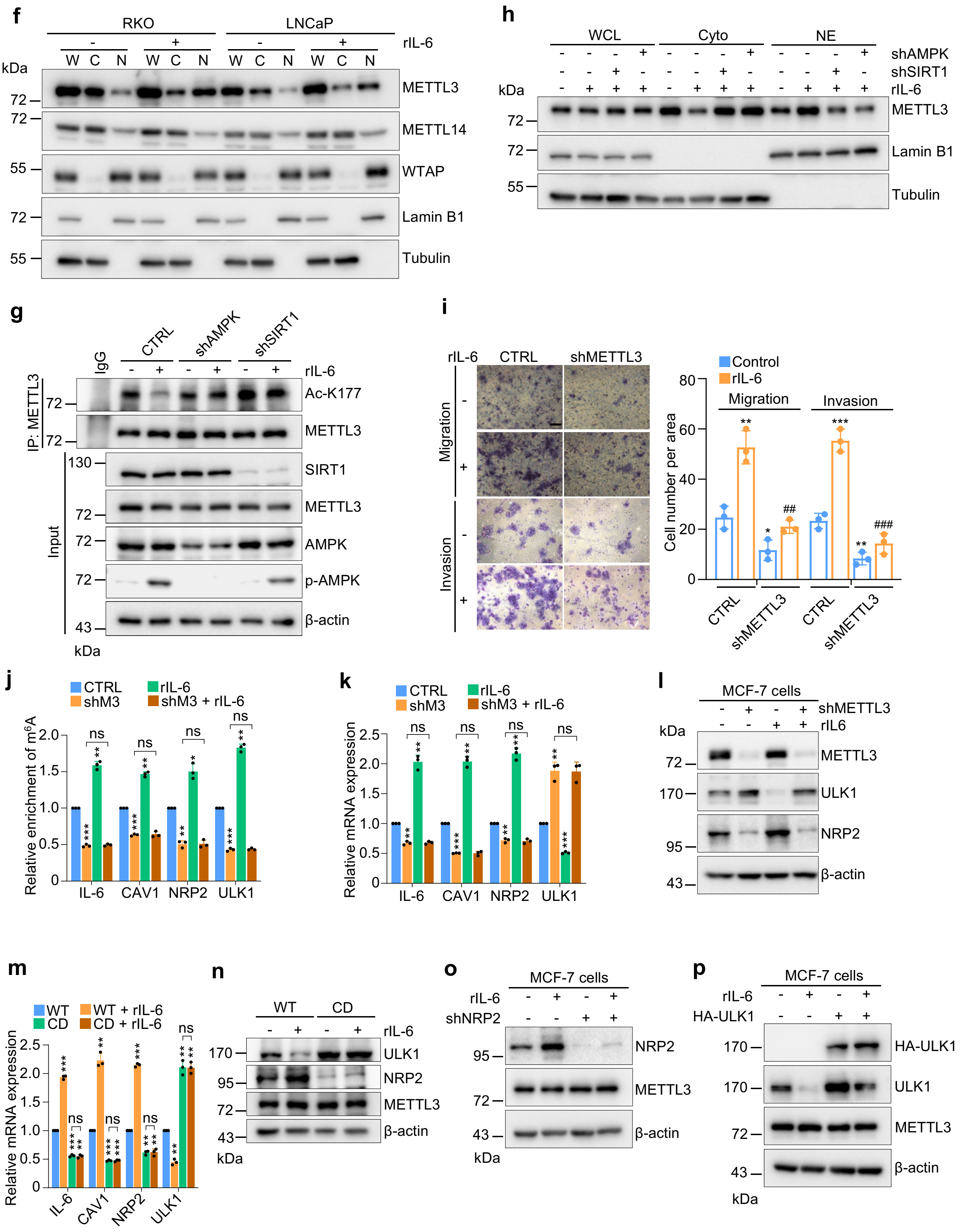

f
RKO
LNCaP
rIL-6
W C N W C N W C N W C N
kDa
METTL3
METTL14
WTAP
Lamin B1
Tubulin
h
WCL
Cyto
NE
shAMPK
shSIRT1
rIL-6
METTL3
Lamin B1
Tubulin
g
IgG
CTRL
shAMPK
shSIRT1
IP: METTL3
Input
Ac-K177
METTL3
SIRT1
AMPK
p-AMPK
β-actin
i
rIL-6
CTRL
shMETTL3
Migration
Invasion
Control
rIL-6
Cell number per area
j
CTRL
rIL-6
shM3
shM3 + rIL-6
Relative enrichment of m6A
IL-6
CAV1
NRP2
ULK1
k
Relative mRNA expression
l
MCF-7 cells
shMETTL3
rIL6
ULK1
NRP2
m
WT
WT + rIL-6
CD
CD + rIL-6
n
WT
CD
o
MCF-7 cells
shNRP2
p
HA-ULK1

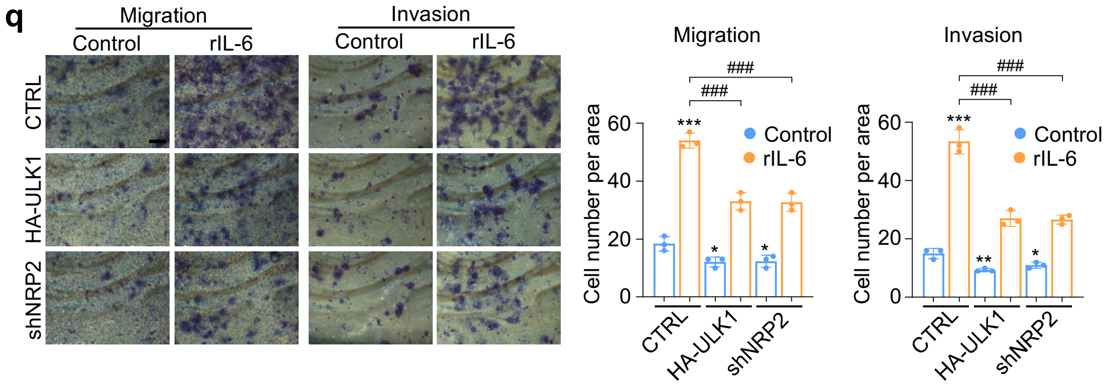

Supplementary Fig. 6 IL-6 facilitates METTL3 nuclear shift and m^{6}A modification through promoting METTL3 deacetylation. a, IB analysis of WCL, Cyto and NE fractions from the indicated tumor cell lines in Figure 6**c**. **b,** Lysates of MCF-7 cells infected with the indicated lentiviruses were subjected to IB analysis. **c,** IB analysis of whole cell lysate, cytoplasmic and nuclear fractions of METTL3 reconstituting MCF-7 cells. **d,** IB analysis of WCL, Cyto and NE fractions of METTL3 reconstituting MCF-7 cells treated with rIL-6 for 12 hours. **e,** IB analysis of WCL, Cyto and NE fractions of MCF-7 cells subjected to rIL-6 treatment following IL-6R knockdown (left panel). IL-6R knockdown efficiency is shown on the right panel. **f,** IB analysis of WCL, Cyto and NE fractions of RKO and LNCaP cells treated with rIL-6. **g,** MCF-7 cells infected with the indicated lentiviruses were treated with rIL-6 for 12 hours, followed by IP and IB analysis. **h,** IB analysis of WCL, Cyto and NE fractions of MCF-7 cells infected with the indicated lentiviruses, followed by rIL-6 treatment for 12 hours. **i,** Migration and invasion assays were conducted in MCF-7 cells infected with the indicated lentiviruses and treated with rIL-6 (left panel) (n = 3 biologically independent experiments). Scale bars, 50μm. Quantifications are shown on the right panel. From left to right for migration: ** P = 0.0038, * P = 0.021, $^{\#\#}$ P = 0.0016; from left to right for invasion: *** P = 0.0005, ** P = 0.0028, $^{\#\#\#}$ P = 0.0003, respectively, by two-sided t-test. **j,** m^{6}A-MeRIP-qPCR analysis of the indicated m^{6}A substrates normalized to input in MCF-7 cells subjected to the indicated treatment (n = 3 biologically independent experiments). IL-6: *** P = 0.0004, ** P = 0.0029, ns P = 0.55; CAV1: *** P = 0.0005, ** P = 0.0017, ns P = 0.79; NRP2: ** P = 0.0032, * P = 0.015, ns P = 0.96; ULK1: *** P = 0.0004, ** P = 0.0013, ns P = 0.75, respectively, by two-sided t-test. **k,** QRT-PCR quantification of the indicated mRNAs in MCF-7 cells subjected to the indicated treatment (n = 3 biologically independent experiments). IL-6: ** P = 0.0028, ** P = 0.0043, ns P = 0.79; CAV1: *** P = 1.52e-08, ** P = 0.0024, ns P = 0.94; NRP2: ** P = 0.0053, ** P = 0.0024, ns P = 0.51; ULK1: ** P = 0.0095, *** P = 0.0004, ns P = 0.42, respectively, by two-sided t-test. **l,** IB analysis of the indicated proteins in MCF-7 cells subjected to the indicated treatment. **m,** QRT-PCR quantification of the indicated mRNAs in METTL3 reconstituting MCF-7 cells treated with rIL-6 for 24 hours (n = 3 biologically independent experiments). IL-6: *** P = 0.0004, *** P = 0.0007, ** P = 0.0013, ns P = 0.55; CAV1: ** P = 0.0035, *** P = 0.0002, *** P = 0.0003, ns P = 0.96; NRP2: *** P = 0.0002, ** P = 0.0032, ** P = 0.0087, ns P = 0.65; ULK1: ** P = 0.0028, ** P = 0.0042, ** P = 0.0026, ns P = 0.91, respectively, by two-sided t-test. **n,** IB analysis of the indicated proteins in METTL3 reconstituting MCF-7 cells treated with rIL-6 for 24 hours. Results are shown as mean ± SD. **o,p,** IB analysis of the indicated proteins in MCF-7 cells subjected to the indicated treatment. **q,** Migration and invasion assays were conducted using MCF-7 cells subjected to the indicated treatment (left panel) (n = 3 biologically independent experiments). Scale bars, 50 μm. Quantification is shown on the right panel. From left to right for migration: *** P = 7.16e-05, * P = 0.023, * P = 0.034, $^{\#\#\#}$ P = 0.0008, $^{\#\#\#}$ P = 0.0008; from left to right for invasion: *** P = 0.0001, ** P = 0.0058, * P = 0.026, $^{\#\#\#}$ P = 0.0008, $^{\#\#\#}$ P = 0.0005, respectively, by two-sided t-test. All data are represented as mean ± SD. All P values were calculated by Student's t test. * compared to CTRL. $^{\#}$ compared to CTRL + rIL-6. Source data are provided as a Source Data file.

Supplementary Figure 7

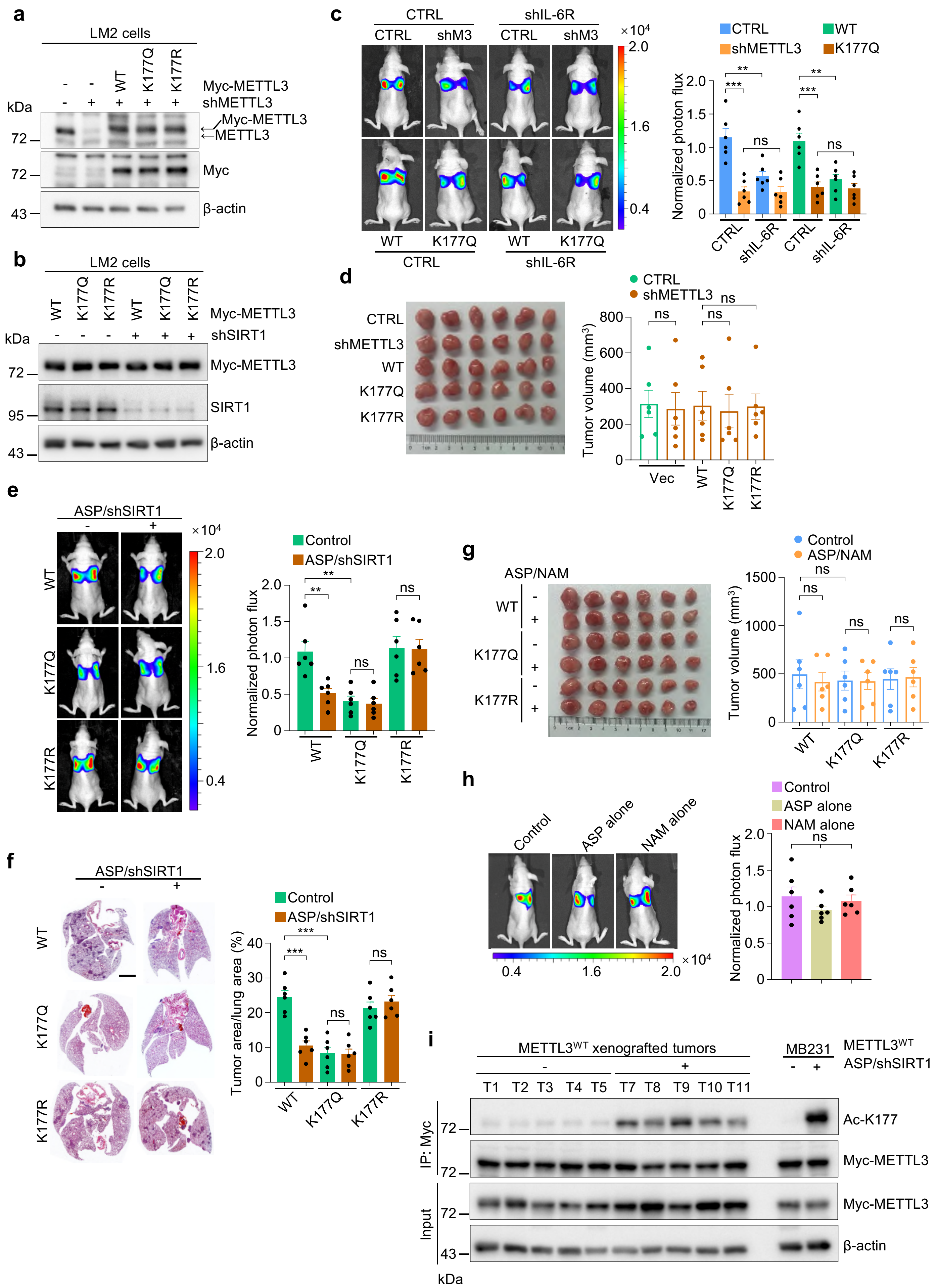

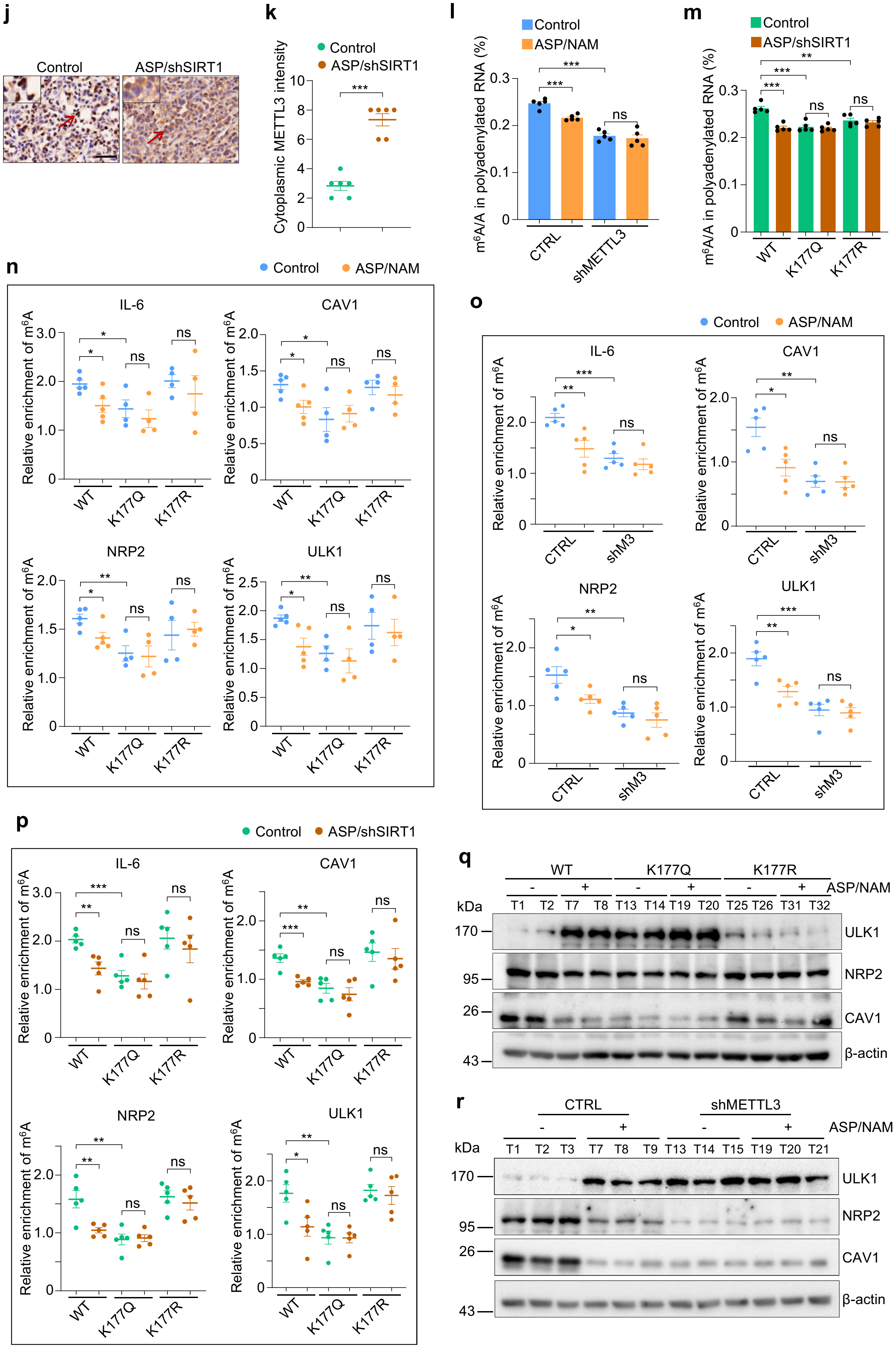

j
Control
ASP/shSIRT1
k
Control
ASP/shSIRT1
Cytoplasmic METTL3 intensity
***
l
Control
ASP/NAM
m6A/A in polyadenylated RNA (%)
***
***
ns
CTRL
shMETTL3
m
Control
ASP/shSIRT1
m6A/A in polyadenylated RNA (%)
**
***
***
ns
ns
WT
K177Q
K177R
n
Control
ASP/NAM
IL-6
CAV1
NRP2
ULK1
Relative enrichment of m6A
WT
K177Q
K177R
o
Control
ASP/NAM
IL-6
CAV1
NRP2
ULK1
Relative enrichment of m6A
CTRL
shM3
p
Control
ASP/shSIRT1
IL-6
CAV1
NRP2
ULK1
Relative enrichment of m6A
WT
K177Q
K177R
q
WT
K177Q
K177R
ASP/NAM
kDa
T1
T2
T7
T8
T13
T14
T19
T20
T25
T26
T31
T32
170
95
26
43
ULK1
NRP2
CAV1
β-actin
r
CTRL
shMETTL3
ASP/NAM
kDa
T1
T2
T3
T7
T8
T9
T13
T14
T15
T19
T20
T21
170
95
26
43
ULK1
NRP2
CAV1
β-actin

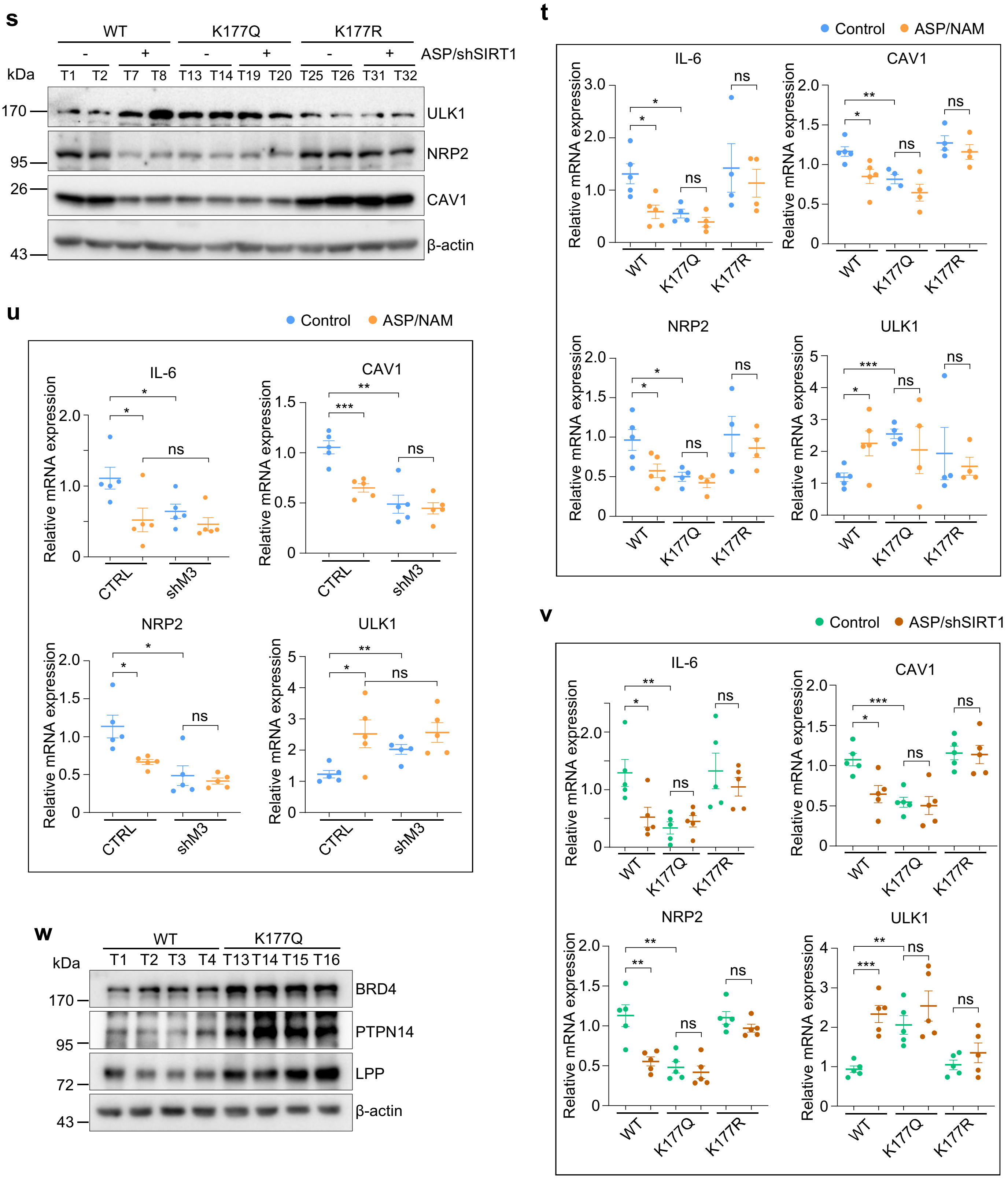
s
WT
K177Q
K177R
-
+
ASP/shSIRT1
kDa
T1 T2 T7 T8 T13 T14 T19 T20 T25 T26 T31 T32
170
95
26
43
ULK1
NRP2
CAV1
β-actin
t
Control
ASP/NAM
IL-6
CAV1
NRP2
ULK1
Relative mRNA expression
WT
K177Q
K177R
ns
u
Control
ASP/NAM
IL-6
CAV1
NRP2
ULK1
Relative mRNA expression
CTRL
shM3
ns
v
Control
ASP/shSIRT1
IL-6
CAV1
NRP2
ULK1
Relative mRNA expression
WT
K177Q
K177R
ns
w
WT
K177Q
kDa
T1 T2 T3 T4 T13 T14 T15 T16
170
95
72
43
BRD4
PTPN14
LPP
β-actin

Supplementary Fig. 7 ASP/NAM modulates expression levels of selected m^6A-modified transcripts and translational targets of METTL3. a, Lysates of LM2 cells infected with the indicated lentiviruses were subjected to IB analysis. **b,** IB analysis of the indicated proteins in METTL3 reconstituting LM2 cells subjected to the indicated treatment. **c,** The indicated LM2 cells were injected into the mammary fat pads of nude mice. Representative bioluminescent images (BLI) of mice with spontaneous lung metastasis (left panel), and quantification of BLI (right panel) are shown. From left to right: *** $P = 0.0004$, ** $P = 0.004$, ns $P = 0.98$, *** $P = 0.0007$, ** $P = 0.0026$, ns $P = 0.84$, respectively, by two-sided *t*-test. $n = 6$ mice per group. **d,** Images of xenogfated tumors in the indicated groups (left panel), and quantification of tumor volume are shown (right panel). From left to right: ns $P = 0.82$, ns $P = 0.80$, ns $P = 0.97$, respectively, by two-sided *t*-test. $n = 6$ mice per group. **e,** METTL3 reconstituting LM2 cells infected with the indicated lentiviruses were injected into the mammary fat pads of nude mice. Representative bioluminescent images (BLI) of mice with spontaneous lung metastasis following daily treatment with ASP for 30 days (left panel), and quantification of BLI (right panel) is shown. From left to right: ** $P = 0.0043$, ** $P = 0.0015$, ns $P = 0.74$, ns $P = 0.93$, respectively, by two-sided *t*-test. $n = 6$ mice per group. **f,** Representative H&E staining (left panel) and quantification (right panel) analysis of lung metastasis in **e**. $n = 6$ mice per group. Scale bars, 2 mm. From left to right: *** $P = 0.0001$, *** $P = 9.26\text{e-}05$, ns $P = 0.90$, ns $P = 0.48$, respectively, by two-sided *t*-test. **g,** Images of xenogfated tumors in the indicated groups (left panel), and quantification of tumor volume are shown (right panel). From left to right: ns $P = 0.67$, ns $P = 0.73$, ns $P = 0.97$, ns $P = 0.89$, respectively, by two-sided *t*-test. $n = 6$ mice per group. **h,** Representative BLI of mice treated as indicated with spontaneous lung metastasis (left panel) and quantification of BLI (right panel). From left to right: ns $P = 0.23$, ns $P = 0.73$, respectively, by two-sided *t*-test. $n = 6$ mice per group. **i,** IP and IB analysis of acetyl-K177 METTL3 levels in xenografted tumors. **j,k,** Representative IHC staining (**j**) and quantification (**k**) of cytoplasmic METTL3 in xenografted tumors. Scale bars, 25 μm. $n = 6$ mice per group. *** $P = 6.06\text{e-}06$ by two-sided *t*-test. **l,m,** LC-MS/MS quantification of the m^6A/A ratio in polyadenylated RNA isolated from xenografted tumors. $n = 5$ mice per group. From left to right **l**: *** $P = 0.0004$, *** $P = 6.29\text{e-}06$, ns $P = 0.57$, from left to right **m**: *** $P = 0.0001$, *** $P = 0.0003$, ** $P = 0.0058$, ns $P = 0.59$, ns $P = 0.51$, respectively, by two-sided *t*-test. **n,o and p,** m^6A-MeRIP-qPCR analysis of the indicated m^6A substrates normalized to input in xenografted tumors. From left to right **n**: IL-6: * $P = 0.025$, * $P = 0.028$, ns $P = 0.46$, ns $P = 0.53$, CAV1: * $P = 0.025$, * $P = 0.021$, ns $P = 0.70$, ns $P = 0.53$, NRP2: * $P = 0.028$, ** $P = 0.0047$, ns $P = 0.81$, ns $P = 0.73$, ULK1: * $P = 0.014$, ** $P = 0.0021$, ns $P = 0.61$, ns $P = 0.73$, from left to right **o**, IL-6: ** $P = 0.01$, *** $P = 0.0002$, ns $P = 0.41$, CAV1: * $P = 0.012$, ** $P = 0.0012$, ns $P = 0.96$, NRP2: * $P = 0.034$, ** $P = 0.0034$, ns $P = 0.41$, ULK1: ** $P = 0.0057$, *** $P = 0.0005$, ns $P = 0.72$, from left to right **p**: IL-6: ** $P = 0.0044$, *** $P = 0.0005$, ns $P = 0.57$, ns $P = 0.56$, CAV1: *** $P = 0.0009$, ** $P = 0.0017$, ns $P = 0.50$, ns $P = 0.65$, NRP2: ** $P = 0.0089$, ** $P = 0.0042$, ns $P = 0.83$, ns $P = 0.52$, ULK1: * $P = 0.032$, ** $P = 0.0037$, ns $P = 0.97$, ns $P = 0.63$, respectively, by two-sided *t*-test. **q,r and s,** IB analysis of the indicated proteins in xenografted tumors. **t,u and v,** QRT-PCR quantification of the indicated mRNAs in xenografted tumors. From left to right **t**: IL-6: * $P = 0.013$, * $P = 0.01$, ns $P = 0.2$, ns $P = 0.61$, CAV1: * $P = 0.02$, ** $P = 0.0049$, ns $P = 0.22$, ns $P = 0.41$, NRP2: * $P = 0.038$, * $P = 0.022$, ns $P = 0.40$, ns $P = 0.55$, ULK1: * $P = 0.034$, *** $P = 0.0003$, ns $P = 0.53$, ns $P = 0.66$, from left to right **u**, IL-6: * $P = 0.032$, * $P = 0.035$, ns $P = 0.76$, CAV1: *** $P = 0.0009$, ** $P = 0.001$, ns $P = 0.71$, NRP2: * $P = 0.015$, * $P = 0.011$, ns $P = 0.61$, ULK1: * $P = 0.024$, ** $P = 0.0037$, ns $P = 0.17$, from left to right **v**: IL-6: * $P = 0.029$, ** $P = 0.0059$, ns $P = 0.45$, ns $P = 0.45$, CAV1: * $P = 0.012$, *** $P = 0.0008$, ns $P = 0.75$, ns $P = 0.89$, NRP2: ** $P = 0.0048$, ** $P = 0.0031$, ns $P = 0.58$, ns $P = 0.20$, ULK1: *** $P = 0.0003$, ** $P = 0.002$, ns $P = 0.32$, ns $P = 0.31$, respectively, by two-sided *t*-test. **w,** IB analysis of the indicated proteins in xenografted tumors. All data are represented as mean ± SEM. All *P* values were calculated by Student's *t*-test. Source data are provided as a Source Data file.

Supplementary Figure 8

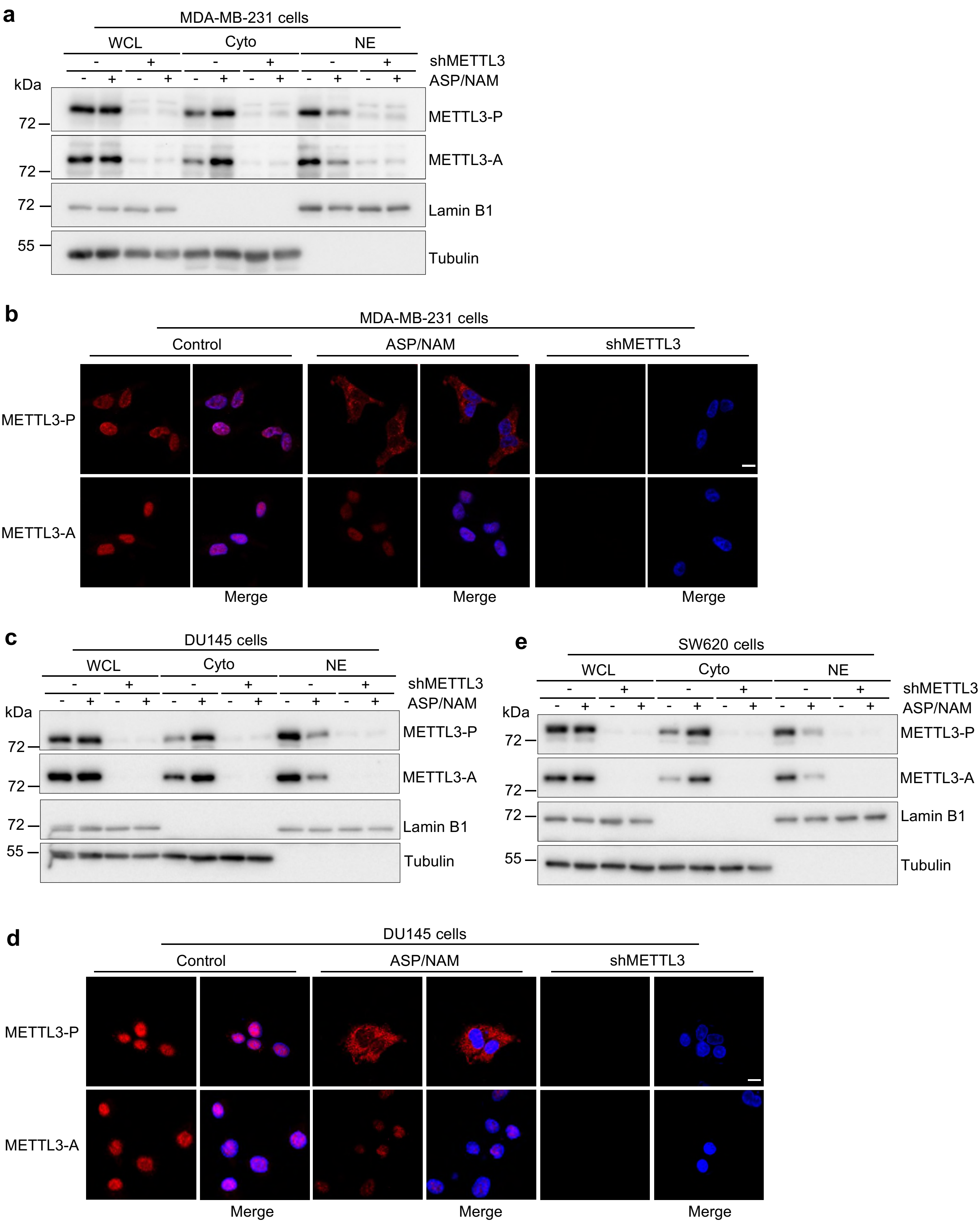

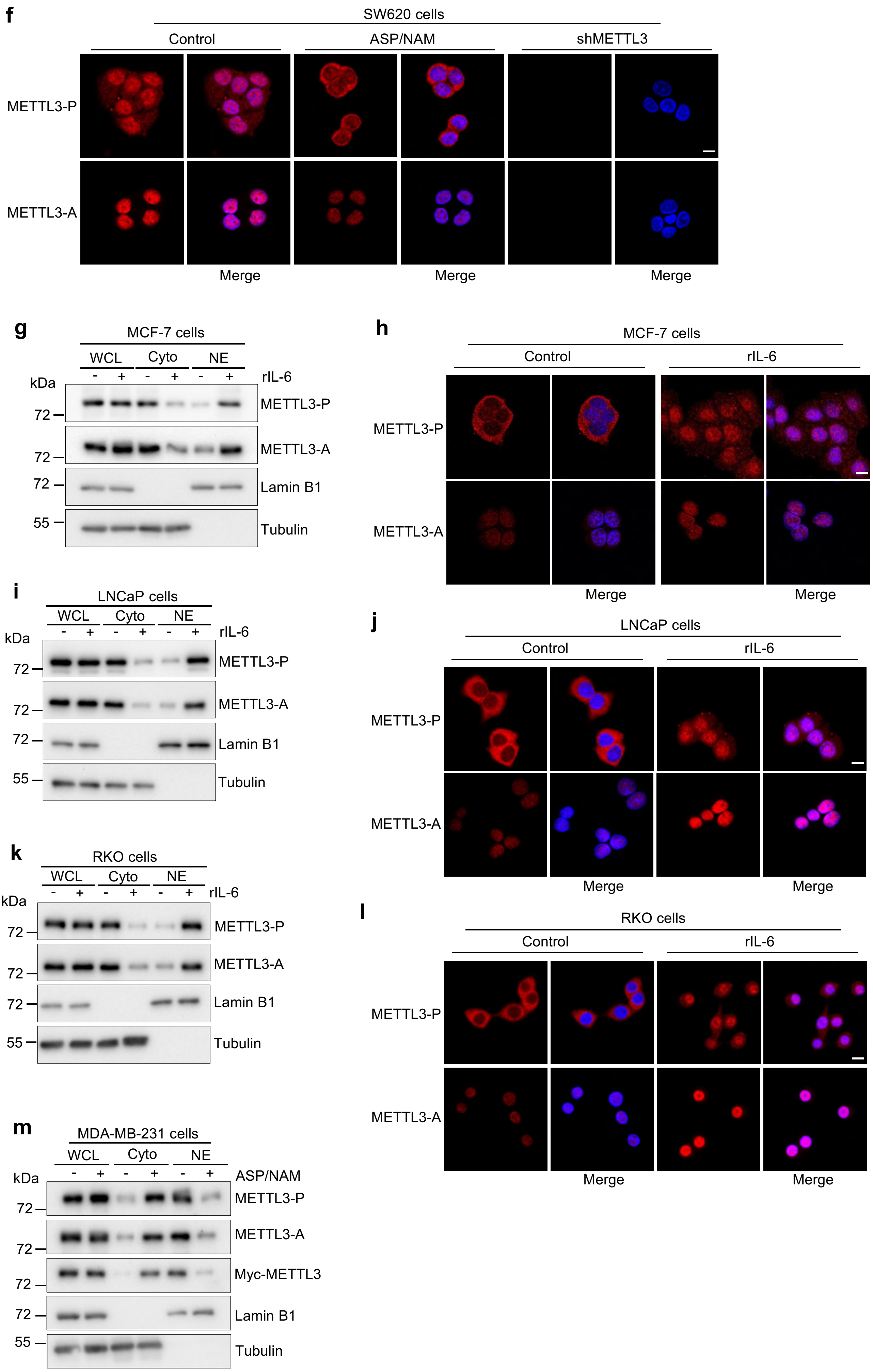
f
SW620 cells
Control
ASP/NAM
shMETTL3
METTL3-P
METTL3-A
Merge
Merge
Merge
g
MCF-7 cells
WCL
Cyto
NE
- + - + - +
rIL-6
kDa
72
72
72
55
METTL3-P
METTL3-A
Lamin B1
Tubulin
h
MCF-7 cells
Control
rIL-6
METTL3-P
METTL3-A
Merge
Merge
i
LNCaP cells
WCL
Cyto
NE
- + - + - +
rIL-6
kDa
72
72
72
55
METTL3-P
METTL3-A
Lamin B1
Tubulin
j
LNCaP cells
Control
rIL-6
METTL3-P
METTL3-A
Merge
Merge
k
RKO cells
WCL
Cyto
NE
- + - + - +
rIL-6
kDa
72
72
72
55
METTL3-P
METTL3-A
Lamin B1
Tubulin
l
RKO cells
Control
rIL-6
METTL3-P
METTL3-A
Merge
Merge
m
MDA-MB-231 cells
WCL
Cyto
NE
- + - + - +
ASP/NAM
kDa
72
72
72
72
55
METTL3-P
METTL3-A
Myc-METTL3
Lamin B1
Tubulin

n

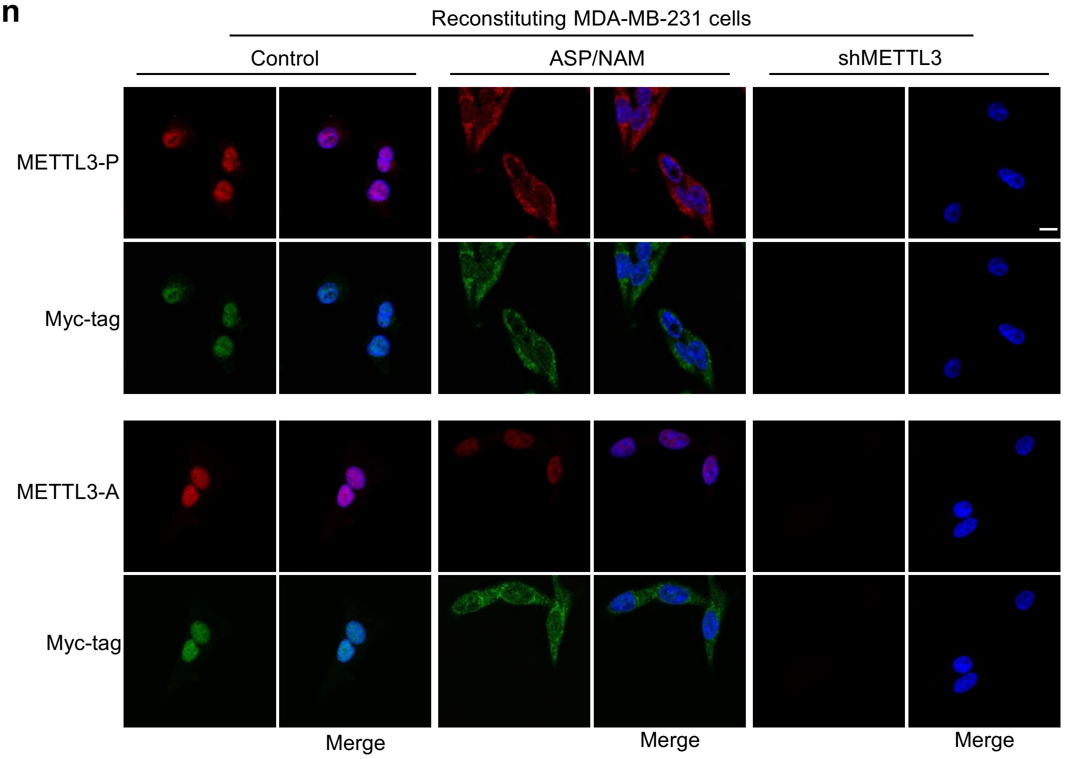

Reconstituting MDA-MB-231 cells
Control
ASP/NAM
shMETTL3
METTL3-P
Myc-tag
METTL3-A
Myc-tag
Merge
Merge
Merge

o

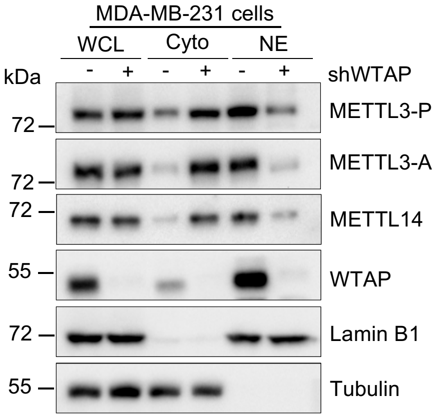

MDA-MB-231 cells
WCL
Cyto
NE
kDa
- + - + - +
shWTAP
72
METTL3-P
72
METTL3-A
72
METTL14
55
WTAP
72
Lamin B1
55
Tubulin

p

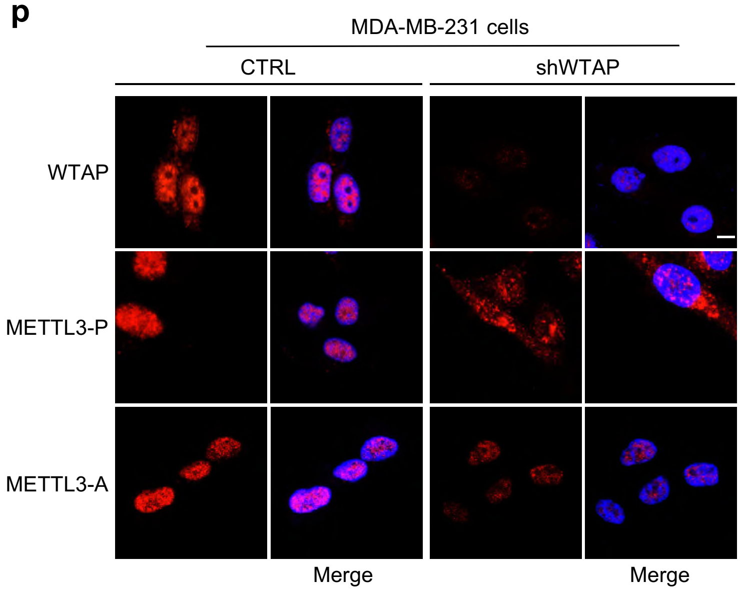

MDA-MB-231 cells
CTRL
shWTAP
WTAP
METTL3-P
METTL3-A
Merge
Merge

q

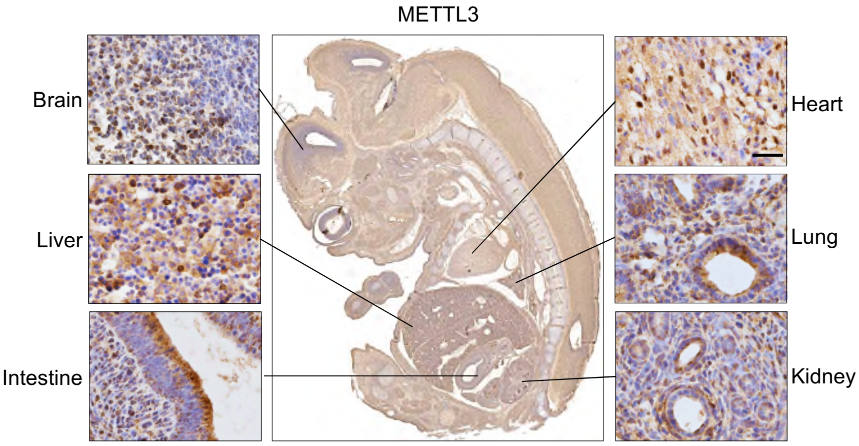

METTL3
Brain
Liver
Intestine
Heart
Lung
Kidney

Supplementary Fig. 8 Detection of cytosolic METTL3 by different antibodies. a, IB analysis of WCL, Cyto and NE fractions from MDA-MB-231 cells subjected to the indicated treatment. **b,** Representative immunofluorescence for METTL3 (red) and DAPI (blue, cell nuclei) in MDA-MB-231 cells subjected to the indicated treatment. Scale bars, 10 μm. $n = 3$ independent experiments. **c,** IB analysis of WCL, Cyto and NE fractions from DU145 cells subjected to the indicated treatment. **d,** Representative immunofluorescence for METTL3 (red) and DAPI (blue, cell nuclei) in DU145 cells subjected to the indicated treatment. Scale bars, 10 μm. $n = 3$ independent experiments. **e,** IB analysis of WCL, Cyto and NE fractions from SW620 cells subjected to the indicated treatment. **f,** Representative immunofluorescence for METTL3 (red) and DAPI (blue, cell nuclei) in SW620 cells subjected to the indicated treatment. Scale bars, 10 μm. $n = 3$ independent experiments. **g,** IB analysis of WCL, Cyto and NE fractions from MCF-7 cells treated with rIL-6. **h,** Representative immunofluorescence for METTL3 (red) and DAPI (blue, cell nuclei) in MCF-7 cells treated with rIL-6. Scale bars, 10 μm. $n = 3$ independent experiments. **i,** IB analysis of WCL, Cyto and NE fractions from LNCaP cells treated with rIL-6. **j,** Representative immunofluorescence for METTL3 (red) and DAPI (blue, cell nuclei) in LNCaP cells treated with rIL-6. Scale bars, 10 μm. $n = 3$ independent experiments. **k,** IB analysis of WCL, Cyto and NE fractions from RKO cells treated with rIL-6. **l,** Representative immunofluorescence for METTL3 (red) and DAPI (blue, cell nuclei) in RKO cells treated with rIL-6. Scale bars, 10 μm. $n = 3$ independent experiments. **m,** IB analysis of WCL, Cyto and NE fractions from METTL3 reconstituting MDA-MB-231 cells treated with ASP/NAM. **n,** Representative immunofluorescence for METTL3 (red), Myc-tag (green) and DAPI (blue, cell nuclei) in METTL3 reconstituting MDA-MB-231 cells treated with ASP/NAM. Scale bars, 10 μm. $n = 3$ independent experiments. **o,** IB analysis of WCL, Cyto and NE fractions from MDA-MB-231 cells infected with shRNA lentiviruses targeting WTAP. **p,** Representative immunofluorescence for METTL3 (red), WTAP (red) and DAPI (blue, cell nuclei) in MDA-MB-231 cells infected with shRNA lentiviruses targeting WTAP. Scale bars, 10 μm. Secondary antibody info: Myc/mouse; M3-P/rabbit; M3-A/rabbit. All primary antibodies for IF staining were used at dilutions of 1:200. $n = 3$ independent experiments. **q,** Representative images of METTL3 IHC staining in E15.5 mouse embryo. Mouse embryos: $n = 6$. Scale bars, 1 mm for low magnification (1 ×), and 25 μm for high magnification (40 ×). Source data are provided as a Source Data file.

Supplementary Table 1. Gene sets from Human Cancer Metastasis Database (HCMDB) utilized in GSEA analysis.

Gene Set Utilized	Gene Name
**HALLMARK_EPITHELIAL MESENCHYMAL TRANSITION**	CD44, VCAN, DAB2, FOXC2, GJA1, CXCL1, IGFBP2, IL6, CXCL8, ITGAV, ITGB3, JUN, LOX, LOXL2, MMP1, MMP2, MMP3, MMP14, TNFRSF11B, SERPINE1, PLAUR, HTRA1, PTHLH, CXCL12, SNAI2, SPP1, TAGLN, TGFB1, TGM2, TIMP3, VCAM1, VEGFA, VEGFC, VIM, WNT5A, ADAM12, SLIT2, CADM1
**HALLMARK_TNFA SIGNALING VIA NFKB**	BIRC2, ATF3, BMP2, BTG1, CD44, CEBPB, KLF6, HBEGF, EGR1, F3, CXCL1, CXCL2, CXCL3, ICAM1, IL6, JUN, SMAD3, MYC, NFKB1, SERPINE1, PER1, PLAU, PLAUR, PTGS2, CCL5, STAT5A, TAP1, TNF, VEGFA, FOSL1, IRS2, SPHK1, KLF4, TRIP10, ACKR3
**HALLMARK_IL6 JAK STAT3 SIGNALING**	BAK1, CBL, CD44, CXCL1, CXCL3, HMOX1, IL2RG, IL6, ITGB3, JUN, LEPR, STAT1, STAT3, TGFB1, TNF, TYK2
**HALLMARK_TGF BETA SIGNALING**	XIAP, RHOA, BMP2, CDH1, CTNNB1, ID1, SMAD3, SERPINE1, SKIL, TGFB1, ARID4B
**GO_Positive Regulation of Cell Motility**	AAMP, ACTN4, AKT1, AKT2, ANXA1, ANXA3, ARF6, RHOA, RHOC, BMP2, BMP4, BMP7, C1QBP, CAV1, CD74, CD151, CCR7, DAB2, DOCK1, HBEGF, EGFR, F2R, F3, PTK2B, FER, FGFR1, VEGFD, FOXC2, FLT4, GATA3, GCNT2, GRN, HGF, HIF1A, HMGB1, HMOX1, HSPA5, HSPB1, ICAM1, IGF1R, IGFBP5, IL6, CXCL8, ITGA6, ITGAV, ITGB3, KDR, LGALS3, LYN, SMAD3, MDK, MMP9, MMP14, NEDD9, DDR2, SERPINE1, PAK1, PDGFB, PFN1, PIK3CG, PLAU, MAPK1, MAPK3, PTGS2, PTK2, RAC1, RAC2, RET, RPS6KB1, S100A7, CCL5, SDCBP, CXCL12, SELE, SNAI2, SNAI1, SOX9, SP1, SRC, STAT3, STAT5A, TAC1, TACR1, TERT, TGFB1, TGFBR2, TNF, TWIST1, VEGFA, VEGFC, WNT5A, CXCR4, IRS2, FADD, NRP2, CCN4, SPHK1, CLDN1, ARHGEF2, CXCL14, BCAR1, IQSEC1, AKT3, GPNMB, SEMA4D, ACKR3, RAB25, PREX1, FERMT3, STK11, SLIT2, ANGPT2, ATP6V1C1, AXL, BMP5, CBL, CBLB, CDC42, EFNB1, ERBB2, ERBB4, EFEMP1, FGFR4, FOXC1, FOXO1, GSK3B, NRG1, IGFBP2, LEP, LOX, MMP2, MST1, MST1R, NTRK1, ENPP1, PIK3CA, PLAUR, PRL, PRLR, PTEN, PTPN3, PXN, ROBO1, SDC2, SHC1, SYK, TSC2, VAV2, YES1, WASL, ADIPOQ, ELMO1, VAV3, AGR2, TXNIP, GIT1, CCDC88A, EPHA10, XIAP, BMP6, RUNX2, CCNA2, CD44, CREB1, CTNNB1, E2F1, EGR1, GDF2, IBSP, ID1, IL10, JUN, SMAD4, MYC, PPARG, HTRA1, SKIL, CLDN5, TP53, VCAM1, KLF4, PPARGC1A, F11R, DLL4, HTRA3

**GO_Chemotaxis**	AGTR1, ALCAM, ANGPT2, ANXA1, RHOA, BMP4, BMP7, BSG, C1QBP, CD74, CCR5, CCR7, CREB1, HBEGF, EFNB1, ERBB2, F3, PTK2B, FER, FGFR1, VEGFD, GATA3, CXCL1, CXCL2, CXCL3, HGF, NRG1, HMGB1, HOXB9, HSPB1, IL6, CXCL8, IL10, ITGAV, ITGB3, KDR, L1CAM, LGALS3, LOX, LYN, SMAD3, SMAD4, MDK, MIF, MST1, NOTCH3, NTRK1, SERPINE1, PDGFB, PIK3CA, PIK3CG, PLAU, PLAUR, MAPK1, MAPK3, PTK2, RAC1, RAC2, RET, ROBO1, S100A7, S100A8, S100A9, SCN1B, CCL5, CCL14, CCL18, CXCL12, SHC1, SRC, SYK, TGFB1, TSC2, VCAM1, VEGFA, VEGFC, EZR, VLDLR, WNT5A, CXCR4, FOSL1, TNFSF11, IRS2, TNFRSF11A, NRP2, SLIT2, CXCL14, BCAR1, VAV3, GPNMB, SEMA4D, EVL, ENAH, ACKR3, PREX1, WNT3A, EPHA10, AKT1, BMP5, CAV1, CD44, CDC42, CEACAM5, HMOX1, ICAM1, ITGA6, LEP, EPCAM, MMP1, MMP9, MMP14, CD200, SDC2, SELE, TNF, YES1, FADD, WASL, F11R
**GO_Regulation of Cell Adhesion**	ACTN4, AKT1, ANGPT2, ANXA1, RHOA, ARHGDIA, ARHGDIB, BMP2, BMP4, BMP6, BMP7, C1QBP, CAV1, CBLB, CD44, CD74, CDC42, CDH1, CDKN2A, CEBPB, CCR7, DOCK1, EFNB1, ERBB2, PTK2B, FOXC2, GATA3, GCNT2, GSK3B, HMGB1, IBSP, ICAM1, IGFBP2, IL6, CXCL8, IL10, ILK, IDO1, IRAK1, ITGA6, ITGAV, JAK1, KDR, LEP, LGALS3, LYN, EPCAM, SMAD3, MDK, MMP14, MUC1, MYB, NEDD9, SERPINE1, PAK1, PDGFB, PIK3CA, PIK3CG, PLAU, PLAUR, PTEN, PTK2, RAC1, RAC2, RAC3, RET, CCL5, CXCL12, SELE, SLC9A1, SNAI2, SOX9, SRC, SYK, TGFB1, TGFBR2, TGM2, TNF, UTRN, VCAM1, VEGFA, WNT5A, YES1, CXCR4, TNFSF11, FADD, DNAJA3, KLF4, ADIPOQ, DLC1, VAV3, GPNMB, SEMA4D, AGR2, F11R, FOXP3, PREX1, PEAK1, CD276, FERMT3, WNT3A, S100A8, S100A9, EZR, AHR, AXL, BAK1, BST2, CD151, KLF6, EGR1, EZH2, LEPR, MIF, NTRK1, PRKDC, RAG2, STAT3, STK11, TAC1, TLR4, TP53, TNFRSF4, IRS2, CADM1, TLR9, DLL4, FER, GRN, HMOX1, IL13RA2, MMP8, CD200, SPHK1, IL1RL1, ADGRF5, CRP, ACE, MAPK1, MAPK3
**GO_Vasculature Development**	AAMP, ADM, AGTR1, AHR, AKT1, ANGPT2, ANXA1, ANXA2, ANXA3, RHOA, BAK1, BMP4, BMP7, BRCA1, BSG, BTG1, CAV1, CDC42, CTNNB1, EGR1, ERBB2, F3, PTK2B, VEGFD, FOXC1, FOXC2, FLT4, GDF2, GRN, HGF, HIF1A, HK2, HMGB1, HMOX1, HSPB1, ID1, IL6, CXCL8, IL10, ITGAV, ITGB3, JAK1, JUN, KDR, LEP, LEPR, LOX, LOXL2, MDK, MMP2, MMP14, NFATC1, SERPINE1, PDGFB, PIK3CA, PIK3CG, PPARG, MAPK1, PRL, PTEN, PTGS2,

	PTK2, PTK7, ROBO1, S100A7, SHC1, SIX1, SP1, STAT1, STK11, SYK, TERT, TGFB1, TGFBR2, CLDN5, TNF, TWIST1, VAV2, VEGFA, VEGFC, WNT5A, CXCR4, ADAM12, FOSL1, HMGA2, RECK, NRP2, SPHK1, KLF4, SLIT2, AKT3, PAK4, YAP1, VAV3, GPNMB, HTATIP2, DLL4, FOXJ2, ACKR3, SOX17, ADGRF5, BMP2, BMP5, BMP6, BRCA2, CEBPB, EGFR, ESR1, FGFR1, GATA3, GJA1, GLI1, SFN, IGFBP5, SMAD3, MST1, MYC, NME1, PGR, SERPINB5, PRKDC, HTRA1, RB1, CXCL12, SNAI2, SOX9, STAT5A, TLR4, TNFSF11, CLDN1, ODAM, WNT3A, RICTOR, ARF6, ARHGDIB, C1QBP, DOCK1, HBEGF, EFNB1, ERBB4, FER, ITGB4, MMP9, DDR2, PAK1, PFN1, PXN, RAC1, RET, SRC, TAC1, PDLIM1, MTA2, BCAR1, IQSEC1, SEMA4D, PTP4A3, EVL, RAB25

Supplementary Table 2. Transcriptionally reduced 26 genes with decreased m^6A peaks overlapped with HCMDB.

**Gene Name**				
BRCA2	GATA3	ITGB4	SDC2	TSC2
CAV1	GSK3B	KIF3C	SETD1A	UBE2V1
CD82	HMGB1	MAPK3	SIX1	
CXCL3	IGF1R	NEDD9	SKIL	
EGR1	IL6	NRP2	SLC9A1	
ENPP1	IRS2	PAK1	TAZ	

Supplementary Table 3. 60 genes with increased translation efficiency overlapped with METTL3 PAR-CLIP.

Gene Name				
ABL2	CPS1	GYS1	MCTP1	SIPA1L3
ADAMTS6	CWF19L2	HECW1	MTMR3	STOM
ANO7	DEPDC5	HECW2	NFX1	TRPC3
ARHGAP23	DET1	HLCS	NHSL1	TSEN2
ASCC3	ERC1	HMBOX1	PAPPA	TTC28
ATP9A	FBXO41	IKBKG	PDE10A	TXNDC16
ATXN1L	FGF8	KDM4C	PDE4D	UPF2
BACH2	FLYWCH1	KIF1B	PLCB1	VPS8
BCR	FNBP1L	KIF26B	PTPN14	WNK1
CBL	FZD1	LARP1	RAPGEF4	ZFHX4
CDH1	GIGYF2	LPP	RC3H1	ZNF12
COL27A1	GREB1L	MAST4	SCAF11	ZNF81

Supplementary Table 4. Primers for QRT-PCR.

Target	Forward primer (5'—3;)	Reverse primer (5'—3')
IL-6	TACATCCTCGACGGCATCTC	AGCTCTGGCTTGTTCCTCAC
CAV1	TACGTAGACTCGGAGGGACAT	TCTGCCATGGCCTTGTTGTT
NRP2	GTGAGAATTTTAAAGTGGACATCCC	GCTCCAGTCCACCTCGTATTC
ULK1	GTTCCAAACACCTCGGTCCT	CCAACTTGAGGAGATGGCGT
BRD4	GACATGAGCACAATCAAGTC	GAACACATCCTGGAGCTTGC
PTPN14	GGGGCCATGTAAGCAGCTAT	TGGCCTTAGCAGTTTCGTGA
LPP	CCCTGCCGAAGTTTCACTTT	CAAAGTTCCCACCTTGCAGC
GAPDH	CCTGACCTGCCGTCTAGAAA	CCCTGTTGCTGTAGCCAAAT

Supplementary Table 5. Primers for MeRIP QRT-PCR.

Target	Forward primer (5'—3')	Reverse primer (5'—3')
IL-6	ACAGGGAGAGGGAGCGATA	CCAGTCCTCTTTGTTGGGGAT
CAV1	TCCCCAAAGGCAGAATCACA	TGAGTCGTACAGAAAGCTGCC
NRP2	GAGGGGCTGGGGAAGATTAC	TCCTGCGACTCGCTTTTCTG
ULK1	CTGTGTGCTGGCTGGACTC	GCTCCAGATGTGCTGGTTCT

Supplementary Table 6. Information regarding the human cohort used in this study.

Breast cancer tissue microarrays:

**Histologic grade (Fig. 1a,c, Fig. S1a,b,c,e)**		
Age		
	Mean (SEM)	52.46 (0.67)
Sex		
	Female	*n* = 291 (100%)
Histologic grade		
	I	*n* = 93 (32%)
	II	*n* = 153 (53%)
	III	*n* = 45 (15%)
N classification		
	N0	*n* = 160 (55%)
	N1-3	*n* = 131 (45%)
Adjacent normal tissues		*n* = 41

**Breast cancer subtypes (Fig. 1b,c)**		
Age		
	Mean (SEM)	48.84 (1.00)
Sex		
	Female	*n* = 70 (100%)
Subtype		
	Luminal	*n* = 36 (51%)
	HER2+	*n* = 20 (29%)
	Basal like	*n* = 14 (20%)
N classification		
	N0	*n* = 28 (40%)
	N1-3	*n* = 42 (60%)

Paired primary and lymphatic metastatic breast cancer tissues (Fig. S1f)	
Age	
Mean (SEM)	50.75 (1.78)
Sex	
Female	*n* = 32 (100%)
Histologic grade	
II	*n* = 32 (100%)

Tumor samples along with their adjacent tissues (Fig. S1d)	
Age	
Mean (SEM)	50.4 (1.61)
Sex	
Female	*n* = 60 (100%)
Histologic grade	
I	*n* = 13 (22%)
II	*n* = 38 (63%)
III	*n* = 9 (15%)
N classification	
N0	*n* = 30 (50%)
N1-3	*n* = 30 (50%)

Frozen breast tumor samples (Fig. 7k):	
Age	
Mean (SEM)	54.36 (3.30)
Sex	
Female	*n* = 11 (100%)
N classification	
N0	*n* = 5 (45%)
N1-3	*n* = 6 (55%)

Prostate cancer tissue microarrays (Fig. S1g,h):

Age	
Mean (SEM)	70.75 (0.61)
Sex	
Male	*n* = 102 (100%)
Gleason grade	
G6-7	*n* = 48 (47%)
G8-10	*n* = 54 (53%)
N classification	
N0	*n* = 86 (84%)
N1-3	*n* = 16 (16%)
Adjacent normal tissues	*n* = 43

Colon cancer tissue microarrays (Fig. S1i,j):

Age	
Mean (SEM)	59.73 (1.07)
Sex	
Female	*n* = 86 (57%)
Male	*n* = 65 (43%)
N classification	
N0	*n* = 60 (40%)
N1-3	*n* = 91 (60%)
Adjacent normal tissues	*n* = 89
